# Phenylpyridyl‐Fused Boroles: A Unique Coordination Mode and Weak B−N Coordination‐Induced Dual Fluorescence

**DOI:** 10.1002/anie.202013692

**Published:** 2021-01-14

**Authors:** Jiang He, Florian Rauch, Alexandra Friedrich, Johannes Krebs, Ivo Krummenacher, Rüdiger Bertermann, Jörn Nitsch, Holger Braunschweig, Maik Finze, Todd B. Marder

**Affiliations:** ^1^ Institute for Inorganic Chemistry and Institute for Sustainable Chemistry & Catalysis with Boron (ICB) Julius-Maximilians-Universität Würzburg Am Hubland 97074 Würzburg Germany

**Keywords:** boroles, dual fluorescence, equilibrium, tetramers, weak intermolecular coordination

## Abstract

Using 4‐phenylpyridine or 2‐phenylpyridine in place of biphenyl, two electron‐poor phenylpyridyl‐fused boroles, **[TipPBB1]_4_** and **TipPBB2** were prepared. **[TipPBB1]_4_** adopts a unique coordination mode and forms a tetramer with a cavity in both the solid state and solution. The boron center of **TipPBB2** is 4‐coordinate in the solid state but the system dissociates in solution, leading to 3‐coordinate borole species. Compared to its borafluorene analogues, the electron‐accepting ability of **TipPBB2** is largely enhanced by the pyridyl group. **TipPBB2** exhibits dual fluorescence in solution due to an equilibrium between free **TipPBB2** and a weak intermolecular coordination adduct with a second molecule. This equilibrium was further investigated by low‐temperature NMR spectroscopy and photophysical studies. Theoretical studies indicate that the highest occupied molecular orbital (HOMO) of **TipPBB2** localizes at the Tip group, in contrast to its borafluorene derivatives, wherein the HOMOs are localized on the borafluorene cores.

## Graphical abstract

Phenylpyridyl‐fused boroles **[TipPBB1]_4_** and **TipPBB2** were synthesized and their properties investigated. **[TipPBB1]_4_** forms a tetramer in both the solid state and solution. **TipPBB2** contains a 4‐coordinate boron atom in the solid state but dissociates to give a 3‐coordinate boron species in solution. **TipPBB2** shows interesting temperature‐dependent dual fluorescence in solution because of the equilibrium between 3‐ and 4‐coordinate boron species due to weak N⋅⋅⋅B intermolecular coordination.

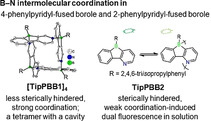

## Introduction

Triarylboranes are of current interest due to their potential applications in various fields, such as non‐linear optics,^[1]^ live cell imaging,[^2a^, ^2b^, ^2c^, ^2d^] DNA/RNA/protein binding,[^2e^, ^2f^] anion sensors,^[3]^ frustrated Lewis pairs (FLPs),^[4]^ etc.,^[5]^ with their properties resulting from the vacant p_z_ orbital on the 3‐coordinate boron atom. This vacant p_z_ orbital can be attacked by nucleophiles, thus 3‐coordinate boranes have been widely used as Lewis acids. It can also act as a π‐acceptor (A). To enhance boron's electron‐accepting ability, Marder^[6]^ and others^[7]^ introduced electron‐withdrawing fluoromesityl (2,4,6‐(CF_3_)_3_C_6_H_2_, ^F^Mes) and related groups at boron. The ^F^Mes group is able to protect the boron center, and thus generate air‐ and moisture‐stable compounds. Another way to enhance the electron‐accepting ability of boron is by embedding it into a 5‐membered diene ring, in compounds known as boroles.^[8]^

In terms of Hückel's rule, boroles are antiaromatic. While the first borole derivative, 1,2,3,4,5‐pentaphenylborole, was reported by Eisch and co‐workers 50 years ago,^[9]^ its single‐crystal structure was determined only recently.^[10]^ Due to the antiaromaticity and strain of the 5‐membered borole ring, the electron‐accepting ability of boron is enhanced and boroles are highly reactive. By benzannulation, the antiaromaticity can be decreased and the stability of boroles improved, which can result in air‐ and moisture‐stable dibenzoboroles.^[11]^ One exception is the family of electron‐rich heteroarene (e.g., thiophene)‐fused boroles, which were reported by Yamaguchi^[12]^ and others.^[13]^ Instead of decreasing antiaromaticity, it was increased, and thus these compounds are air‐ and moisture‐sensitive. The drawback of decreasing the antiaromaticity by benzannulation is that the electron‐accepting ability is also decreased. Fusing electron‐withdrawing aromatic systems onto boroles is another efficient way to increase their electron‐accepting ability. Marder and others enhanced the electron‐accepting ability of boroles by introducing CF_3_ groups at either their *exo*‐aryl^[14]^ moiety or at both the biphenyl core and the *exo*‐aryl moiety of a dibenzoborole.^[15]^

**Scheme 1:**
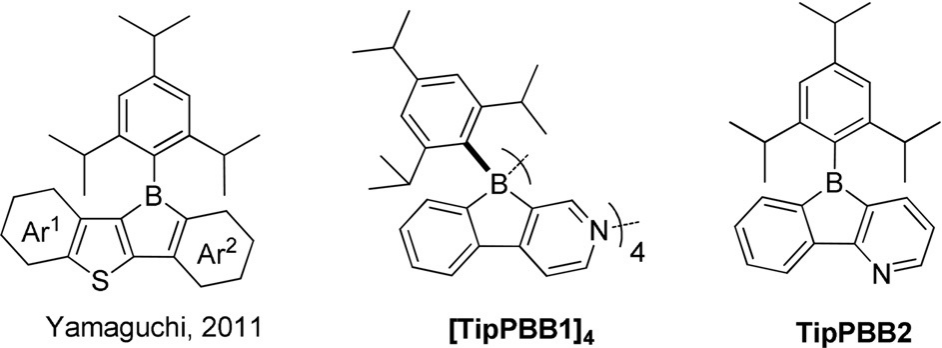
Electron‐rich thiophene‐fused boroles^[12]^ and electron‐poor pyridyl‐fused boroles in this study.

Compared to benzene, pyridine is electron withdrawing due to the higher electronegativity of N vs. C. Using pyridine as one of the substituents in triarylboranes enhances the acceptor strength of boron, as demonstrated by the reduction potentials of pyridine‐containing triarylboranes,^[16]^ 9‐borylated acridine^[17]^ and boron‐doped polycyclic aromatic hydrocarbons (PAHs) with pyridine bound to boron.^[18]^ The coordination ability of pyridine can be used for further functionalization. Thus, triarylborane‐functionalized N,C‐chelate or N,N‐chelate Pt^[19]^ and N,C‐chelate Ir complexes^[20]^ were studied and applied in optoelectronic devices. Wang and co‐workers also found that triarylborane‐functionalized N,C‐chelated tetrahedral boron centers display reversible photo‐thermochromic properties.^[21]^ Liu and co‐workers reported an example of a pyridine‐substituted monobenzofused 1,4‐azaborine, which forms a dimer.^[22]^ In the present study, we synthesized two isomers of phenylpyridyl‐fused boroles, **[TipPBB1]_4_** and **TipPBB2** (Scheme 1), and investigated their structural, photophysical and electrochemical properties.

## Synthesis and crystal structures

**Scheme 2:**
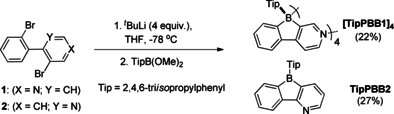
Synthesis of **[TipPBB1]_4_** and **TipPBB2**.

**[TipPBB1]_4_** and **TipPBB2** were synthesized from the corresponding precursors **1** or **2** in a single step (Scheme 2).^[23]^ Both derivatives are moderately air‐stable, decomposing slowly when exposed to air. **[TipPBB1]_4_** is a white solid with a ^11^B{^1^H} NMR signal at 2.9 ppm in CDCl_3_, which indicates that the boron is 4‐coordinate in solution.

**Figure 1:**
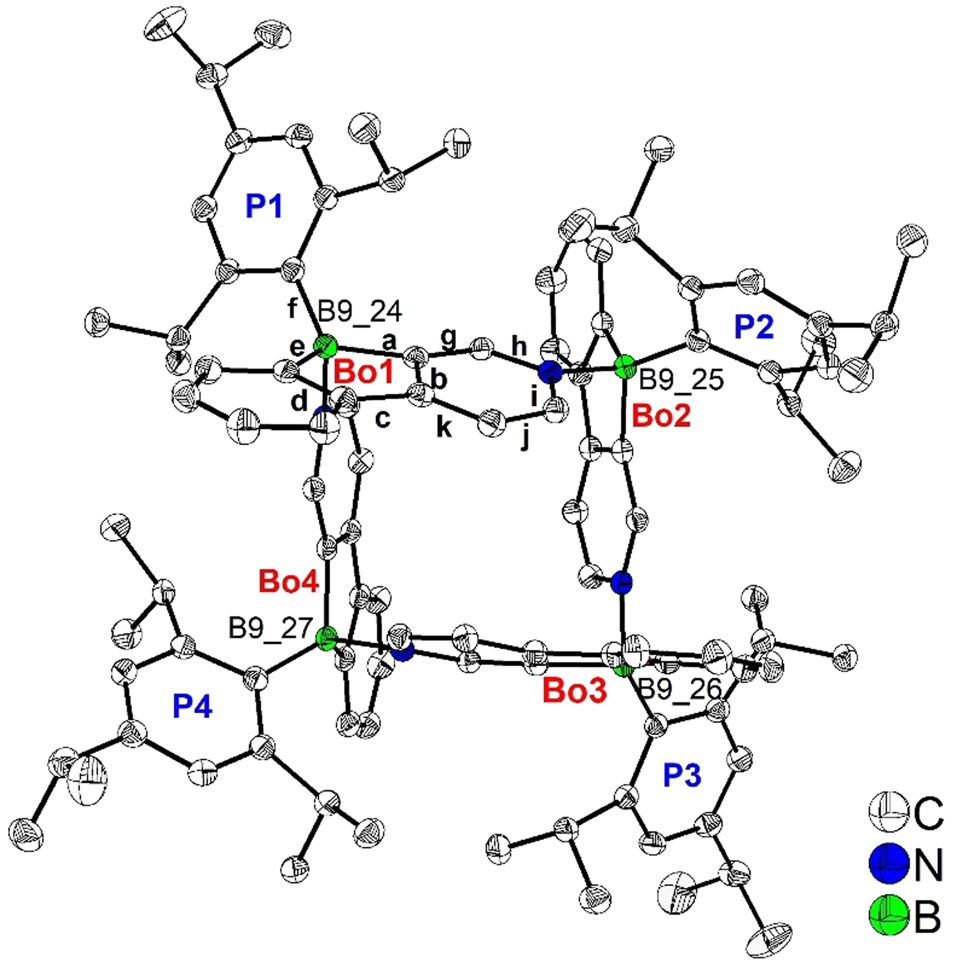
Molecular structure of **[TipPBB1]_4_** in the solid state at 100 K. Atomic displacement ellipsoids are drawn at the 50 % probability level, and H atoms as well as C_6_D_6_ solvent molecules are omitted for clarity. “Bo” and “P” denote the planes of the phenylpyridyl‐fused borole and the Tip phenyl groups, respectively. Selected B−C and C−C bond distances are labeled with letters.

Colorless crystals of **[TipPBB1]_4_** suitable for X‐ray diffraction were grown by evaporation of a C_6_D_6_ solution at room temperature (Figure 1). The pyridine moiety from one molecule coordinates to the boron atom of the neighboring molecule such that four monomers form a tetramer with an empty central cavity. The square‐like (B‐C‐C‐N‐)_4_ coordination mode is similar to that in dimethyl(3‐pyridyl)borane and diethyl(3‐pyridyl)borane.^[24]^ The four 2,4,6‐tri*iso*propylphenyl (Tip) groups of the tetramer are oriented towards the outside of the cavity. The phenylpyridyl‐fused borole core is nearly planar. The torsion angles between the planes of these core moieties and the Tip groups attached to boron range from 57.80(4)–67.53(4)° (Table 1). Adjacent borole planes are approximately perpendicular to one another with torsion angles of 78.44(2)–89.70(2)°. The opposing borole planes have angles of 33.29(3) and 33.21(2)° between the respective pairs of planes. This configuration is similar to the two‐fold rotational symmetry of the opposing planes in diethyl(3‐pyridyl)borane, while the opposing planes in dimethyl(3‐pyridyl)borane exhibit an inverted orientation. The torsion angles between the phenylpyridyl‐fused borole and the plane of the tetramer spanned by the four boron atoms range from 71.94(2)–74.87(2)°, hence, exhibiting a similar configuration to that of the pyridine rings in diethyl(3‐pyridyl)borane. In the 5‐membered borole rings of the tetramer, the B−C bond distances to the pyridyl rings (1.643(2)–1.662(2) Å) are significantly longer than those to the phenyl rings (1.621(2)–1.633(2) Å, Table 1), as observed for other heteroarene‐fused boroles.^[12b]^ The shorter B−C bond distances are in the same range as those in dimethyl(3‐pyridyl)borane (1.612(4)–1.638(3) Å) and in diethyl(3‐pyridyl)borane (1.608(6)–1.642(5) Å).^[24]^ The B−N bond distances (1.644(2)–1.655(2) Å) are in the same range as those found in pentaphenylborole⋅2,6‐lutidine (1.6567(3) Å)^[25]^ and sterically hindered dibenzoborole⋅pyridine (1.638(3) Å).^[26]^ Interestingly, in contrast to those adducts which dissociate in solution at room temperature, the tetramer of **TipPBB1** persists in C_6_D_6_ even at +50 °C. The C−C bonds of the borole moiety (bonds b and d in Figure 1) which are shared with the pyridine and the phenyl ring have lengths typical for aromatic bonds, while the connecting C−C bond (c) is significantly longer, comparable to a C*sp* ^2^−C*sp* ^2^ single bond. The distances between two adjacent boron atoms are 5.365(2)–5.420(2) Å, slightly longer than the corresponding distances in dimethyl(3‐pyridyl)borane (5.267(4) and 5.286(4) Å) and diethyl(3‐pyridyl)borane (5.124(6) and 5.313(5) Å).^[24]^

**Table 1:** Selected bond lengths [Å] and angles [°] of **[TipPBB1]_4_** (according to Figure 1).

	Bo1	Bo2	Bo3	Bo4
**a**: B−C10_Bo_	1.656(2)	1.643(2)	1.662(2)	1.655(2)
**e**: B−C13_Bo_	1.630(2)	1.621(2)	1.633(2)	1.628(2)
**f**: B−C1_Tip_	1.639(2)	1.628(2)	1.645(2)	1.639(2)
**b**: C10−C11 (Bo)	1.409(2)	1.412(2)	1.413(2)	1.408(2)
**c**: C11−C12 (Bo)	1.467(2)	1.474(2)	1.467(2)	1.472(2)
**d**: C12−C13 (Bo)	1.410(2)	1.413(2)	1.412(2)	1.412(2)
B−N	B_Bo1_−N_Bo4_ 1.655(2)	B_Bo2_−N_Bo1_ 1.652(2)	B_Bo3_−N_Bo2_ 1.644(2)	B_Bo4_−N_Bo3_ 1.644(2)
B−B	B_Bo1_−B_Bo2_ 5.420(2)	B_Bo2_−B_Bo3_ 5.365(2)	B_Bo3_−B_Bo4_ 5.407(2)	B_Bo4_−B_Bo1_ 5.402(2)
Bo−P	65.64(3)	57.80(4)	67.53(4)	60.14(3)
Bo−Bo ⊥	Bo1−Bo2 89.70(2)	Bo2−Bo3 81.78(2)	Bo3−Bo4 89.33(2)	Bo4−Bo1 78.44(2)
Bo−Bo ∥	Bo1−Bo3 33.29(3)	Bo2−Bo4 33.21(2)		
Bo−B_4_ plane	72.08(3)	71.94(2)	74.73(3)	74.87(2)

To investigate the coordination mode in solution, we performed a diffusion‐ordered spectroscopy (^1^H DOSY) study in C_6_D_6_. All signals appeared on the same horizontal axis with log(*D* (diffusion constant))=−9.35 (log(m^2^ s^−1^)) at +25 °C. Even at +50 °C, the signals are still on the same horizontal axis with log(*D*)=−9.18 (log(m^2^ s^−1^); Supporting Information, Figures S5 and S6), which indicates that only one species is present in solution. Using the diffusion constants, the van der Waals radius, *r*≈7.63 at +25 °C and 7.78 Å at +50 °C, of **[TipPBB1]_4_** was calculated using the Stokes‐Einstein equation *r*=$\frac{k{}_{B}T}{6\pi \eta D}$ (*k_B_* is the Boltzmann constant, *T* is temperature (K), *η* is the dynamic viscosity (ca. 0.64 and 0.46 MPa s of C_6_D_6_ at +25 and +50 °C, respectively) and *r* is the van der Waals radius).^[27]^ The molecular volume of **[TipPBB1]_4_** as a tetramer was calculated to be 1351.79(15) Å^3^ in the solid state using the Olex2 program package (the following element radii were used: C=1.7 Å; H=1.09 Å; B=2 Å; N=1.55 Å).^[28]^ The van der Waals radius was estimated to be approximately 6.86 Å using the equation *V*=$\frac{4}{3}$ π*r* ^3^, which nicely matches the volume derived from the DOSY study, and thus the tetramer persists in solution.

As it seemed clear that the position of the nitrogen atom in the annulated pyridine ring is crucial for the unique coordination mode of **[TipPBB1]_4_**, we switched the position of N, to hinder intermolecular coordination, and obtained **TipPBB2**. In CDCl_3_, the ^11^B{^1^H} NMR signal of **TipPBB2** was observed at 72.8 ppm, which is in the typical range of 3‐coordinate boron. All attempts to obtain crystals of **TipPBB2** using different solvents were unsuccessful. The solid‐state ^11^B{^1^H} RSHE/MAS NMR study (128 MHz) resulted in a signal with an isotropic chemical shift of 4.4 ppm, which shows that the boron center of **TipPBB2** is 4‐coordinate in the solid state, most likely a result of oligomerization or polymerization via B−N bonds. It is unlikely that a different donor is attached to boron.

## Electrochemical properties

**Figure 2:**
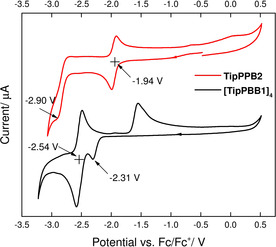
Cyclic voltammetry diagrams of **[TipPBB1]_4_** (black line) and **TipPBB2** (red line). Measured in THF in the presence of 0.1 M TBAPF_6_; scan rates of 250 mV s^−1^ with potentials given vs. the Fc/Fc^+^ couple.

The cyclic voltammogram of **[TipPBB1]_4_** exhibits two reduction potentials (Figure 2). The first reduction (*E*_pc_=−2.31 V) is irreversible and is comparable to that of the reported phenylpyridyl core chelate 4‐coordinate organoboron compounds, and the reduction is attributed to the phenylpyridyl core.^[29]^ The second reduction is partially reversible with a half‐wave reduction potential of *E*_1/2_=−2.54 V. The ratio of the integrals of the first and the second reductions is ca. 1 to 3. After reduction, the integration ratio between the corresponding first and second oxidations is ca. 2 to 2 (the second oxidation peak may constitute two or more overlapping peaks). We suggest that the two reduction processes arise from different phenylpyridyl cores.

**TipPBB2** also exhibits two reduction potentials. The first reduction is reversible with a half‐wave reduction potential of *E*_1/2_^red^=−1.94 V which can be attributed to the borole unit. The first reduction potential of **TipPBB2** is comparable to that of 9‐^F^Mes‐borafluorene (^**F**^ **MesBf**, *E*_1/2_^red^=−1.93 V), and is ca. 0.4 V more positive than that of 9‐Tip‐borafluorene (**TipBf**, *E*_1/2_^red^=−2.36 V).[^11a^, ^14b^] The second reduction is irreversible, with the peak at *E*_pc_=−2.90 V.[^11b^, ^12b^] The two reduction processes indicate the generation of a stable radical anion and the irreversible generation of a dianion. From the first reduction potential, we conclude that the effect of the pyridyl group in boroles on their reducibility is comparable to that of the *exo*‐^F^Mes group, and both of them greatly enhance the electron‐accepting ability of boron.

## Photophysical properties

**Figure 3:**
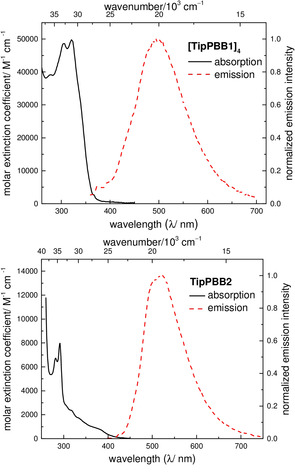
UV/Vis absorption (black solid line) and emission (red dashed line) spectra of **[TipPBB1]_4_** (top) and **TipPBB2** (bottom) in THF.

One of the interesting photophysical properties of fused‐boroles is their weakly allowed lowest energy absorption,^[30]^ which extends into the visible region of the spectrum. This absorption is attributed to p_π_–π* conjugation through the vacant p‐orbital of boron. The intermolecular coordination mode of **[TipPBB1]_4_** interrupts this conjugation, so **[TipPBB1]_4_** does not exhibit this weakly allowed transition (Figure 3, top, and Table 2). This is in line with previously reported 4‐coordinate dibenzoboroles.[^11a^, ^14b^, ^15^] The lowest energy absorption of **[TipPBB1]_4_** is located at 322 nm (ϵ=49 600 cm^−1^ M^−1^) in THF and emission occurs at 495 nm, which is slightly red shifted compared to that in a hexane solution (λ_em_=446 nm). The quantum yield of **[TipPBB1]_4_** is ca. Φ_F_=0.1 and the lifetime is ca. 6 ns in both THF and hexane. It was previously reported by Marder and co‐workers^[15]^ and Rupar and co‐workers^[31]^ that dissociation of a 4‐coordinate borole to a 3‐coordinate borole in the excited state leads to a very similar long lifetime as for the 3‐coordinate borole. Thus, the short lifetime of **[TipPBB1]_4_** suggests that the coordination persists in solution even in the excited state.

**Table 2:** Photophysical data for **[TipPBB1]_4_** and **TipPBB2** at room temperature.

	Solvent	λ_abs_^[a]^ [nm] (*ϵ* [10^3^ cm^−1^ M^−1]^)	λ_em_ [nm]	Φ_F_^[b]^	τ_F_ [ns] (rel.%)^[c]^	Stokes shift [cm^−1^]
**[TipPBB1]_4_**	hexane	304 323	446	0.11	5.7	8540
	THF	306 (46.7); 322 (49.6)	495	0.12	6.3	10 850
**TipPBB2**	hexane	317; 375	514	0.34	5.7 (2.5 %); 165 (97.5 %)	6180
	CH_2_Cl_2_	319; 375	516	0.26	7.3 (6.6 %); 145 (93.4 %)	7290
	THF	320 (2.3); 375 (0.9)	520	0.27	5.6 (5.0 %); 151 (95.0 %)	7440
	MeCN	318; 375	528	0.21	5.8 (3.9 %); 146 (96.1 %)	7730
	solid state	385^c^	520	0.05	6.1 (61.6 %); 87.7 (38.4 %)	6740

[a] Lowest energy absorption maximum. [b] Absolute fluorescence quantum yields measured using an integrating sphere. [c] Lowest energy emission maximum. Percentages in brackets were obtained at the emission wavelength of 520 nm with excitation at 377 nm. The ratios of the lifetimes are dependent on the emission wavelength.

**TipPBB2** presents a small extinction coefficient (ca. ϵ=900 cm^−1^ M^−1^) for the lowest energy absorption at ca. 375 nm and tails to ca. 425 nm in THF, which is similar to those of previously reported dibenzoboroles.[^12a^, ^14b^] The emission of **TipPBB2** has a maximum at 520 nm with a quantum yield of ca. Φ_F_=0.27 in THF, which is much higher than those of **TipBf** and most reported dibenzoboroles.[^12a^, ^14b^] In MeCN, the lowest energy absorption and emission maxima of **TipPBB2** are comparable to the ones in THF, which suggests that MeCN does not coordinate to **TipPBB2**.[^11a^, ^15^] In MeCN, the quantum yield of **TipPBB2** is still Φ_F_=0.21. In the solid state, **TipPBB2** has a low quantum yield of 0.05. Interestingly, **TipPBB2** shows two fluorescence lifetimes at 520 nm in solution at room temperature, e.g., in CH_2_Cl_2_, one long (145 ns (93.4 %)), which is in agreement with the weakly allowed lowest energy transition (Strickler‐Berg relation),^[32]^ and one short (7.3 ns (6.6 %)).

The two lifetimes of **TipPBB2** indicate two radiative decay processes. The purity was confirmed by NMR spectroscopy and elemental analysis and, in addition, several independently synthesized samples showed the same phenomenon, thus ruling out impurities as being responsible for the dual fluorescence. Due to the bulky Tip group and the fact that almost no red shift of the emission was observed with increasing polarity of the solvent (from hexane to MeCN, Table 2), the two decay processes are unlikely to be caused by dual emission from twisted intramolecular charge transfer or planar intramolecular charge transfer.^[33]^ Due to the low concentration (ca. 2×10^−5^ M) at which the measurement was performed, we can rule out the formation of nanoparticle‐induced emission (also termed aggregation‐induced emission (AIE)).^[34]^

**Figure 4:**
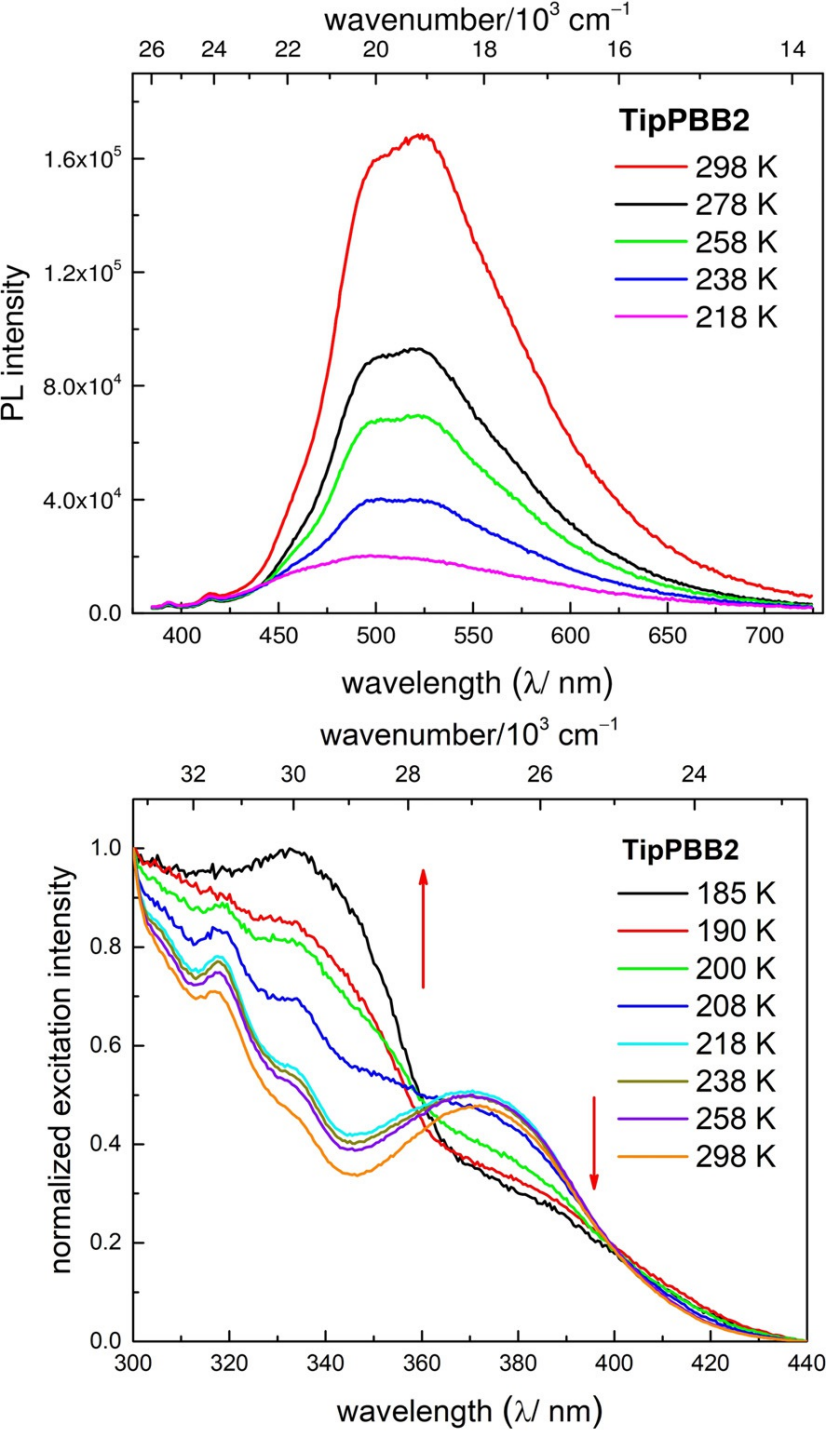
Temperature dependence of the emission spectra in 2‐MeTHF (top) and temperature dependence of the excitation spectra (bottom) with emission at 520 nm in CH_2_Cl_2_.

To gain further insight into those processes, we measured the temperature dependence of the emission spectra and lifetime in 2‐MeTHF (Figure 4, top). The intensity of the emission decreases when the temperature is lowered and, at the same time, the relative percentage of the short lifetime (%_ST_) increases until the long lifetime (%_LT_) at 520 nm disappears completely at ca. 168 K. The decrease of the fluorescence intensity with temperature is unusual, as non‐radiative decay processes are impeded at lower temperature. We suggest that the two lifetimes are caused by the dual fluorescence originating from 3‐coordinate **TipPBB2** and a weak intermolecular adduct of **TipPBB2**, and that the emission spectrum is an overlay of two independent emission bands. This is supported by the emission wavelength dependence of the ratio of the two lifetimes, as well as the change of the shape of the emission with decreasing temperature (Figure S29), and broadness (ca. 400–700 nm) of the emission band. As the ratio of %_ST_ to %_LT_ is dependent on the emission wavelength this does not correlate directly with the ratio of the species in the sample. The intermolecular adduct is generated by weak coordination via a B−N bond, which is favored at lower temperatures. A similar phenomenon was observed for the coordination of THF to a carbene‐stabilized borafluorenium cation.^[35]^ When the temperature is decreased, the equilibrium shifts towards the adduct of **TipPBB2** and, thus, the emission becomes weaker. To support our hypothesis, fluorescence lifetime measurements at 520 nm were performed at different concentrations (2×10^−5^, 1×10^−4^ and 5×10^−4^ M in CH_2_Cl_2_, Figure S30). At 298 K, the ratios of %_ST_ to %_LT_ show negligible change with concentration (Supporting Information, Table S3) but, at 213 K, the more concentrated samples show an increase of %_ST_. Thus, at lower temperatures, adduct formation increases due to the *T*Δ*S* term, which leads to a stronger effect of the concentration on the relative lifetime ratios. Furthermore, temperature dependent excitation spectra in CH_2_Cl_2_ were recorded (Figure 4, bottom). The excitation spectra do not show a large difference until the temperature is decreased to 218 K. When the temperature is decreased further, the intensity of the band at ca. 380 nm decreases and, at the same time, the intensity of the band at ca. 340 nm increases. The change of the shape of the excitation spectra indicates that the observed phenomenon is due to a change in the ground state. This is different from previously reported examples of dual fluorescence arising from photodissociation of the B−N bond in the excited state.^[36]^ Furthermore, we recorded the low‐temperature ^1^H and ^11^B{^1^H} NMR spectra from +25 °C to −90 °C in CD_2_Cl_2_. In the ^1^H NMR spectra, new signals start to appear when the temperature is decreased to −50 °C, but 3‐coordinate **TipPBB2** remains the major species even when the temperature is decreased to −90 °C (Figure S10). Among these new signals, are three new characteristic peaks at *δ* (ppm) 0.72 (d, *J=*7 Hz), 0.36 (d, *J=*7 Hz) and 0.11 (d, *J=*7 Hz) (Figure S10, red box), which can be assigned to the methyl groups of the *ortho*‐*iso*propyl moieties. This phenomenon is concentration dependent. At lower concentrations, at low temperature, no new signals were observed (Figure S13). This excludes the new peaks arising from conformers. The upfield shift of the peaks is likely a result of intermolecular coordination leading to a shielding effect by ring‐current effects of the 4‐coordinate borole core.^[12a]^ The same effect was also observed for **[TipPBB1]_4_**, as three methyl groups of the *ortho*‐*iso*propyl moieties resonate in the same range (*δ* (ppm) 0.95 (d, *J=*6 Hz), 0.77 (d, *J=*6 Hz), 0.30 (d, *J=*6 Hz)). In the ^11^B{^1^H} NMR spectra, the signal from the 3‐coordinate boron becomes very broad at ca. −50 °C, but no 4‐coordinate boron was observed, even when the temperature was decreased to −90 °C (Figure S11). As the geometry around boron will be distorted from tetrahedral due to the constrained nature of the ring system, and the system does not have high symmetry, it is possible that the signal corresponding to the 4‐coordinate species is also broad, similar to that observed for **[TipPBB1]_4_**. To simulate the 4‐coordinate species, 4‐dimethylaminopyridine (DMAP) was added to **TipPBB2** (adduct formation was confirmed by in situ ^1^H and ^11^B{^1^H} NMR spectra in CDCl_3_, Figures S14 and S15). Upon addition of excess of DMAP to a solution of **TipPBB2** in THF, the emission maximum blue shifts to 490 nm (Figure S27) with only a single lifetime of τ_F_=6.8 ns; no long lifetime component was observed. The lifetime of the DMAP‐**TipPBB2** adduct is comparable to the short lifetime component of **TipPBB2** in THF. Interestingly, a linear relationship of the ratio of %_LT_ to %_ST_ vs. temperature of **TipPBB2** in 2‐MeTHF was observed (Figure S32, Table S4).

## Theoretical studies

**Figure 5:**
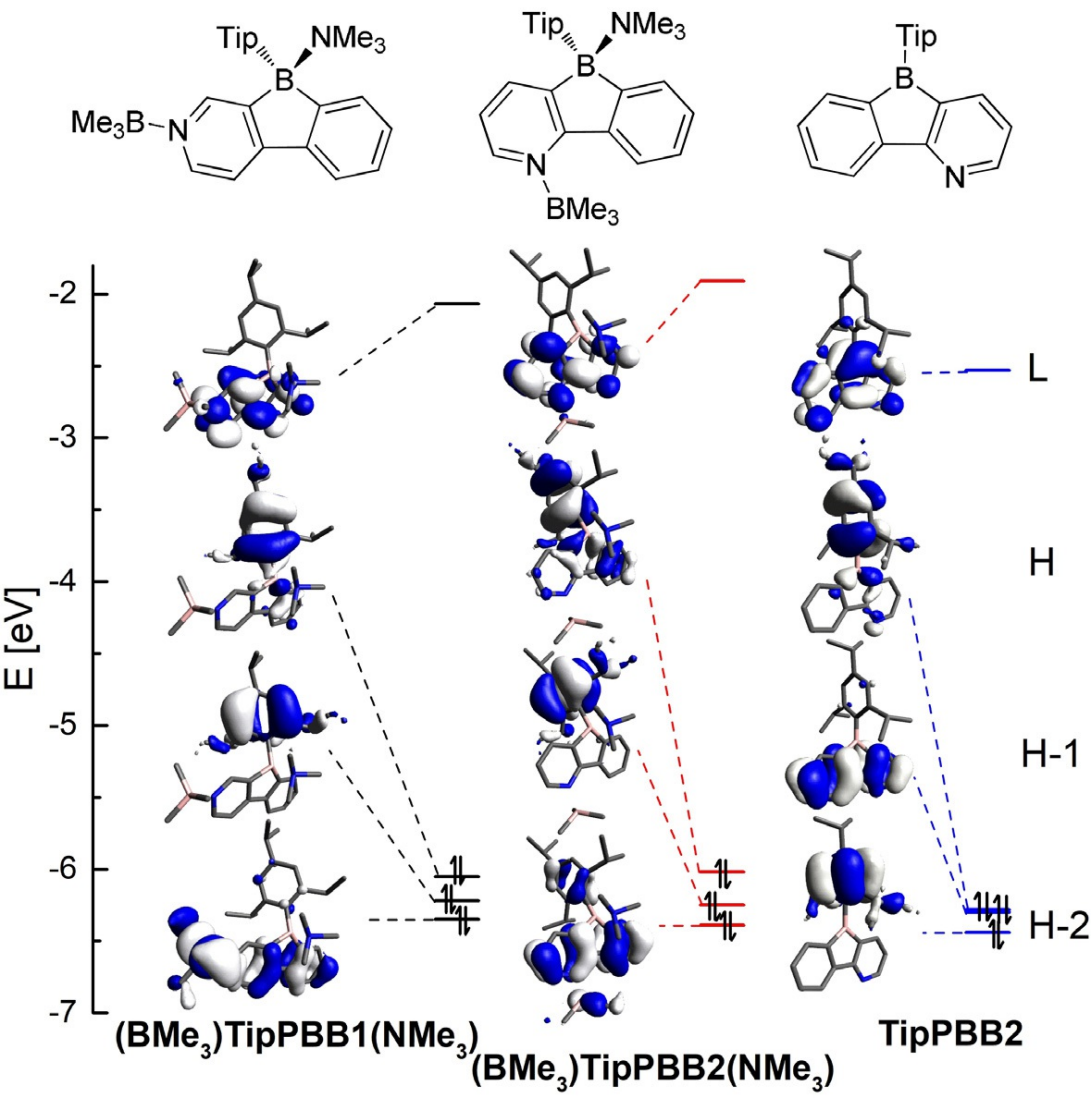
Depiction of the LUMO (L), HOMO (H), HOMO−1 (H‐1) and HOMO−2 (H‐2) of **(BMe_3_)TipPBB1(NMe_3_)**, **(BMe_3_)TipPBB2(NMe_3_)** and **TipPBB2**.

To gain deeper insight into the electronic properties, TD‐DFT and DFT calculations were carried out on **TipPBB2**. In order to reduce the calculation costs, two models, (**(BMe_3_)TipPBB1(NMe_3_)** and **(BMe_3_)TipPBB2(NMe_3_)**), which utilize a BMe_3_ group as the Lewis acid coordinated to pyridine and an NMe_3_ group as the Lewis base coordinated to the boron center of the borole, were used to model **[TipPBB1]_4_** and 4‐coordinate **TipPBB2**, respectively (Figure 5; for computational details, see the Supporting Information).^[37]^ In addition, the influence of *exo*‐aryl groups and the isomers of **TipPBB2** were also examined (Supporting Information).

The orbitals associated with **(BMe_3_)TipPBB1(NMe_3_)** and **(BMe_3_)TipPBB2(NMe_3_)** are quite similar. In both compounds, the NMe_3_ group coordinates to the boron atom and interrupts the p_π_(B)–π* conjugation; therefore, the LUMOs of **(BMe_3_)TipPBB1(NMe_3_)** and **(BMe_3_)TipPBB2(NMe_3_)** (−2.07 eV and −1.91 eV, respectively) are delocalized over the phenylpyridyl core. Moreover, the HOMOs of **(BMe_3_)TipPBB1(NMe_3_)** and of **(BMe_3_)TipPBB2(NMe_3_)** (−6.05 eV and −6.02 eV, respectively) are mainly located at the Tip group with a small contribution from the phenyl group of the phenylpyridyl core. The HOMO−1 is located at the Tip group for both models. The HOMO−2 of **(BMe_3_)TipPBB1(NMe_3_)** (−6.35 eV) delocalizes over the phenylpyridyl core with a large contribution from the BMe_3_ group. However, the HOMO−2 associated with **(BMe_3_)TipPBB2(NMe_3_)** (−6.39 eV) is delocalized over the phenylpyridyl core with some contribution from the Tip and the BMe_3_ groups. In the gas phase, the HOMO and HOMO−1 to LUMO transitions associated with **(BMe_3_)TipPBB1(NMe_3_)** correspond to the S_1_←S_0_ and S_2_←S_0_ transitions, which are weakly allowed with small oscillator strengths (Table 3, *f*=0.0195 and 0.0108, respectively). The HOMO−2 to LUMO transition (S_3_←S_0_) is allowed (*f*=0.1167) with an energy gap of 3.77 eV (*λ*=329 nm), which fits the UV/Vis absorption spectrum of **[TipPBB1]_4_** well. For **(BMe_3_)TipPBB2(NMe_3_)**, the HOMO to LUMO transition corresponds to S_1_←S_0_ which is weakly allowed (*f*=0.016) with an energy of 3.47 eV (*λ*=357 nm).

**Table 3:** Transitions of (BMe_3_)TipPBB1(NMe_3_), (BMe_3_)TipPBB2(NMe_3_) and TipPBB2.

Compound	Transition	*E* [eV]	*Λ* [nm]	*f*	Major contributions	Λ
**(BMe_3_)TipPBB1(NMe_3_)**	S_1_←S_0_ S_2_←S_0_ S_3_←S_0_	3.36 3.62 3.77	358 343 329	0.0195 0.0108 0.2167	H→L (99 %) H‐1→L (99 %) H‐2→L (96 %)	0.31 0.20 0.63
**(BMe_3_)TipPBB2(NMe_3_)**	S_1_←S_0_ S_2_←S_0_ S_3_←S_0_	3.47 3.81 3.80	357 326 318	0.016 0.008 0.0039	H→L (98 %) H‐1→L (99 %) H‐3→L (97 %)	0.36 0.20 0.18
**TipPBB2**	S_1_←S_0_ S_2_←S_0_ S_3_←S_0_	2.92 2.95 3.25	424 420 382	0.0 0.0002 0.0004	H→L (98 %) H‐1→L (98 %) H‐2→L (99 %)	0.27 0.65 0.21

The LUMO (−2.53 eV) of **TipPBB2** delocalizes over the phenylpyridyl core with a large contribution from boron, which leads to it being 0.62 eV lower in energy than the LUMO of **(BMe_3_)TipPBB2(NMe_3_)**. The LUMO of **TipPBB2** is similar to the LUMO of **TipBf** which delocalizes over the biphenyl core with a large contribution from the boron.[^11a^, ^14b^] The HOMO (−6.28 eV) and HOMO−1 (−6.30 eV) have almost the same energy, but the orbital distributions are quite different. The HOMO of **TipPBB2** is mainly localized on the Tip group and is different from previously reported **TipBf**,[^11a^, ^14b^] but similar to ***p*** **‐NMe_2_‐^F^Xyl^F^Bf**, which possesses a strongly donating amine group at the *para*‐position of the *exo*‐aryl moiety.^[15]^ HOMO−1 of **TipPBB2** is similar to the HOMO of **TipBf** which is distributed over the phenylpyridyl‐fused borole core. Thus, by changing the biphenyl core to the phenylpyridyl core, the Tip group becomes a donating group, not just a protecting group. The HOMO−2 (−6.44 eV) of **TipPBB2** is also located on the Tip group. In the gas phase, for **TipPBB2**, the S_1_←S_0_ transition is attributed to a HOMO to LUMO transition which is forbidden. The S_2_←S_0_ and S_3_←S_0_ transitions of **TipPBB2** are weakly allowed with very small oscillator strengths (*f*=0.0002 and 0.0004, respectively), which is in line with the small extinction coefficient observed in the UV/Vis spectrum in the corresponding range (above 375 nm).

## Conclusion

Two boroles with a phenylpyridyl core were synthesized. **[TipPBB1]_4_** was obtained as a tetramer with a central cavity both in the solid state and in solution. The cavity formed by the coordination may potentially be enlarged by expanding the fused system. **TipPBB2** was obtained as a 4‐coordinate species in the solid state but dissociates to a 3‐coordinate species in solution. To the best of our knowledge, **TipPBB2** is the only example of an electron‐poor 3‐coordinate heteroarene‐fused borole. Due to the electron‐withdrawing property of the pyridyl group, the electron‐accepting ability of **TipPBB2** (*E*_1/2_^red^=−1.94 V) is largely enhanced, as demonstrated by cyclic voltammetry. Interestingly, **TipPBB2** shows dual fluorescence in solution. We suggest that the dual fluorescence is caused by an equilibrium between 3‐coordinate **TipPBB2** and a weak intermolecular adduct formed by B−N coordination, which was investigated by photophysical and low‐temperature NMR studies. Theoretical studies indicate that the HOMO of **TipPBB2** is located on the Tip group which is different from that of **TipBf** for which the HOMO is located at the biphenyl main core.

## Conflict of interest

The authors declare no conflict of interest.

## Supporting information

As a service to our authors and readers, this journal provides supporting information supplied by the authors. Such materials are peer reviewed and may be re‐organized for online delivery, but are not copy‐edited or typeset. Technical support issues arising from supporting information (other than missing files) should be addressed to the authors.

Supplementary
