# Supplementary material for: Phenylpyridyl‐Fused Boroles: A Unique Coordination Mode and Weak B−N Coordination‐Induced Dual Fluorescence
Source: Angew Chem Int Ed Engl. 2021 Jan 14;60(9):4833–40. doi: 10.1002/anie.202013692 (PMC7986225; doi:10.1002/anie.202013692)
Supplement: Supplementary file 1 — Supplementary [file ANIE-60-4833-s001.pdf]

Angewandte Chemie
Eine Zeitschrift der Gesellschaft Deutscher Chemiker

Supporting Information

Phenylpyridyl-Fused Boroles: A Unique Coordination Mode and Weak B−N Coordination-Induced Dual Fluorescence

Jiang He, Florian Rauch, Alexandra Friedrich, Johannes Krebs, Ivo Krummenacher, Rüdiger Bertermann, Jörn Nitsch, Holger Braunschweig, Maik Finze, and Todd B. Marder**

anie_202013692_sm_miscellaneous_information.pdf

Table of contents

General information S1

Synthesis S5

NMR spectra S7

Single-crystal X-ray diffraction S15

Photophysical properties S17

Cyclic voltammetry S22

TD-DFT Calculations S24

References S58

General information

All starting materials were purchased from commercial sources and used without further purification or prepared following reported procedures. All solvents for synthetic reactions and for photophysical and electrochemical measurements were HPLC grade, further treated to remove trace water using an Innovative Technology Inc. Pure-Solv Solvent Purification System and deoxygenated using the freeze-pump-thaw method. All synthetic reactions were performed in an Innovative Technology Inc. glovebox or under an argon atmosphere using standard Schlenk techniques.

Reactions were monitored using thin layer chromatography (TLC) plates pre-coated with a layer of silica (Polygram® Sil G/UV254) with fluorescent indicator UV254. Column chromatography was performed using aluminiumoxide 90 neutral as the stationary phase and the solvent system indicated.

General characterization

^{1}H, $^{13}C\{^{1}H\}$ and $^{11}B\{^{1}H\}$ NMR and diffusion-ordered spectroscopy (DOSY) spectra were measured at 298 K using a Bruker Avance I 500 MHz (^{1}H, 500 MHz; ^{13}C, 125 MHz; ^{11}B, 160 MHz) or Bruker Avance III 400 MHz (^{1}H, 400 MHz; ^{13}C, 101 MHz; ^{11}B, 128 MHz) NMR spectrometer or Bruker Avance Neo 400 MHz (^{1}H, 400 MHz; ^{1}H DOSY NMR only (298 and 323 K)). Chemical shifts (*δ*) were referenced to solvent peaks as follows: ^{1}H NMR spectra were referenced to residual protonated solvent in $CDCl_3$ (7.26 ppm); $^{13}C\{^{1}H\}$ spectra were referenced to $CDCl_3$ (77.26 ppm); and $^{11}B\{^{1}H\}$ NMR signals were referenced to external $BF_3{\cdot}OEt_2$. DOSY spectra were measured in C_6D_6 (7.16). Solid-state magic angle spinning (MAS) NMR spectra were recorded using a Bruker Avance Neo WB 400 solid-state NMR spectrometer using 4 mm (o. d.) ZrO_2 rotor (^{11}B: 128 MHz). Elemental analyses were performed on an Elementar vario MICRO cube elemental analyzer in our Institute. Carbon analysis of **TipPBB2** was up to 1.8% below the calculated value, while hydrogen and nitrogen were satisfactory. This may be ascribed to the formation of boron carbide.[1] High resolution mass spectrometry (HRMS) and LIFDI was performed with a Thermo Fisher Scientific Exactive Plus

Orbitrap MS System with atmospheric pressure chemical ionization (APCI) or electrospray ionization (ESI).

Single-crystal X-ray diffraction

Crystals suitable for single-crystal X-ray diffraction were selected, coated in polybutyl oil (**[TipPBB1]$_4$**) mounted on a polyimide microloop (MicroMounts from MiTeGen) and transferred to a stream of cold nitrogen (Oxford Cryostream 800). Diffraction data for **[TipPBB1]$_4$** were collected on a Rigaku Oxford Diffraction XtaLAB Synergy diffractometer with a semiconductor HPA-detector (HyPix-6000) and multi-layer mirror monochromated Cu-K$_\alpha$ radiation at 100 K. The images were processed and corrected for Lorentz-polarization effects and absorption as implemented in the CrysAlis^{Pro} software from Rigaku Oxford Diffraction. The structures were solved using the intrinsic phasing method (SHELXT)[2] and Fourier expansion technique. All non-hydrogen atoms were refined in anisotropic approximation, with hydrogen atoms "riding" in idealized positions, by full-matrix least squares against F^2 of all data, using SHELXL[3] software and the SHELXLE graphical user interface.[4] Diamond[5] software was used for graphical representation.[6] Crystal data and experimental details are listed in Table S1; full structural information has been deposited with Cambridge Crystallographic Data Centre. CCDC-2013331 (**[TipPBB1]$_4$**).

Photophysical measurements

All measurements were performed under an argon atmosphere using standard quartz cuvettes (1 cm × 1 cm cross-section). UV-visible absorption spectra were recorded using Agilent 8453 (**[TipPBB1]$_4$**) or LAMBDA 465 (**TipPBB2**) diode array UV-visible spectrophotometers. Molar extinction coefficients were calculated from three independently prepared samples. Excitation and emission spectra were recorded using an Edinburgh Instruments FLSP920 spectrometer equipped with a double monochromator for both excitation and emission, operating in right-angle geometry mode, and all spectra were fully corrected for the spectral response of the instrument.

The concentration of **[TipPBB1]$_4$** was lower than 10^{-5} M to minimize inner filter effects during fluorescence measurements. Due to the weak absorption of **TipPBB2**, the solutions for fluorescence measurements were measured at a concentration of ca. $2x10^{-5}$ M.

Fluorescence quantum yield measurements

Fluorescence quantum yields were measured using a calibrated integrating sphere (150 mm inner diameter) from Edinburgh Instruments combined with the FLSP920 spectrometer described above. For solution-state measurements, the longest wavelength absorption maximum of the compound in the respective solvent was chosen for excitation.

Lifetime measurements

Fluorescence lifetimes were recorded via the time-correlated single photon counting (TCSPC) method using an Edinburgh Instruments FLS920 spectrometer equipped with a high-speed photomultiplier tube positioned after a single emission monochromator. Measurements were made in right-angle geometry mode, and the emission was collected through a polarizer set to the magic angle. Both compounds were excited with either pulsed diode lasers at wavelengths of 316 nm or 377 nm at repetition rates of 10 or 0.5 MHz, respectively. The full-width-at-half-maximum (FWHM) of the pulse from the diode laser was ca. 90 ps with an instrument response function (IRF) of ca. 1 ns FWHM, respectively. The IRFs were measured from the scatter from pure solvent. Decays were recorded to 10000 counts in the peak channel with a record length of at least 1000 channels. The band pass of the emission monochromator and a variable neutral density filter on the excitation side were adjusted to give a signal count rate of <60 kHz. Iterative reconvolution of the IRF with one decay function and non-linear least-squares analysis were used to analyze the data. The quality of decay fits was judged to be satisfactory based on the calculated values of the reduced χ^2 and Durbin-Watson parameters and visual inspection of the weighted residuals.

Electrochemical measurements

Cyclic voltammetry experiments were performed using a Gamry Instruments Reference 600 potentiostat. A standard three-electrode cell configuration was employed using a platinum disk working electrode, a platinum wire counter electrode, and a silver wire, separated by a *Vycor* tip, serving as the reference electrode. Formal redox potentials are referenced to the ferrocene/ferrocenium redox couple. Tetra-*n*-butylammonium hexafluorophosphate ([*n*-Bu_4N][PF_6]) was employed as the supporting electrolyte. Compensation for resistive losses (*iR* drop) was employed for all measurements.

Theoretical studies

All calculations (DFT and TD-DFT) were carried out with the Gaussian 09 (9.E.01)[7] program package and were performed on a parallel cluster system. GaussView (6.0.16), Avogadro (1.2.0)[8] and multiwfn[9] were used to visualize the results, to measure calculated structural parameters, and to plot orbital surfaces (isovalue: ± 0.030 $[eÅ^{-3}]^{1/2}$). The ground-state geometries were optimized using the B3LYP functional[10] in combination with the 6-31G basis set.[11] The D3 dispersion correction of Grimme and co-workers was used.[12] The polarizable continuum model (PCM) was used to include solvent effects for the ground state structures. The ultrafine integration grid and symmetry constraints were used for all molecules. Frequency calculations were performed on the optimized structures to confirm them to be local minima showing no negative (imaginary) frequencies. Based on these optimized structures, the lowest-energy vertical transitions (gas-phase) were calculated (singlets, 25 states) by TD-DFT, using B3LYP in combination with the 6-31+G(d) basis set.[11]

Synthesis

[9-(2,4,6-tri*iso*propylphenyl)-9H-benzo[4,5]borolo[2,3-c]pyridine]$_4$ ([TipPBB1]$_4$)

To a solution of 3-bromo-4-(2-bromophenyl)pyridine[13] (310 mg, 1.0 mmol) in THF (15.0 mL) was added ^{t}BuLi (4.0 mmol) at –78 °C. The resulting orange solution was stirred at –78 °C for 1 h. Dimethyl (2,4,6-tri*iso*propylphenyl)boronate (TipB(OMe)$_2$, 1 mmol, 276 mg) in THF (2.0 mL) was added dropwise and then the reaction was slowly warmed to r.t. and stirred at r.t. overnight. The volatiles were removed under vacuum, and the product was purified by rapid column chromatography on Al_2O_3 in air eluting with CH_2Cl_2/MeOH = 20/1. The light-yellow solid was then dissolved in CH_2Cl_2, crystalized by slow diffusion of hexane under an argon atmosphere as it slowly decomposes in the air. **[TipPBB1]$_4$** was obtained in 22% yield (81 mg) as a white solid. ^{1}H NMR (400 MHz, $CDCl_3$, ppm): δ 7.78-7.72 (m, 1H), 7.60-7.56 (m, 1H), 7.56-7.52 (m, 1H), 7.39 (dt, J = 7, 1 Hz, 1H), 7.34 (dt, J = 7, 1 Hz, 1H), 7.23 (s, 1H), 7.15-7.10 (m, 1H), 6.99 (d, J = 2 Hz, 1H), 6.83 (d, J = 2 Hz, 1H), 2.84 (sept, J = 7 Hz, 1H), 2.62 (sept, J = 6 Hz, 1H), 2.38 (sept, J = 6 Hz, 1H), 1.26 (d, J = 7 Hz, 3H), 1.25 (d, J = 7 Hz, 3H), 1.18 (d, J = 6 Hz, 3H), 0.95 (d, J = 6 Hz, 3H), 0.77 (d, J = 6 Hz, 3H), 0.30 (d, J = 6 Hz, 3H); ^{13}C{^{1}H} NMR (125 MHz, $CDCl_3$, ppm): δ 159.8, 158.4, 157.2, 154.0, 153.1, 145.8, 144.0, 143.3, 140.6, 139.3, 133.4, 131.5, 128.2, 122.4, 121.6, 120.3, 115.2, 33.7, 32.7, 32.5, 28.3, 26.3, 24.2, 24.1, 23.8, 23.8; ^{11}B{^{1}H} NMR (160 MHz, $CDCl_3$, ppm): δ 2.9 (br). LIFDI (ESI$^+$): m/z calcd for $[C_{104}H_{120}B_4N_4]^+$: 1468.9880; Found: 1468.9920 $[M]^+$; Elem. Anal. Calcd (%) for $C_{26}H_{30}BN$: C, 85.01; H, 8.23; N, 3.81; Found: C, 84.87; H, 8.25; N, 3.98.

5-(2,4,6-tri*iso*propylphenyl)-5H-benzo[4,5]borolo[3,2-b]pyridine (TipPBB2)

To a solution of 3-bromo-2-(2-bromophenyl)pyridine[13] (310 mg, 1.0 mmol) in THF (15.0 mL) was added ^{t}BuLi (4.0 mmol) at –78 °C. The resulting green solution was stirred at –78 °C for 1 h. Dimethyl (2,4,6-tri*iso*propylphenyl)boronate (TipB(OMe)$_2$, 1 mmol, 276 mg) in THF (2.0 mL) was added dropwise and then the reaction was slowly warmed to r.t. and stirred at r.t. overnight. The volatiles were removed under vacuum,

and the product was purified by rapid column chromatography on Al_2O_3 in air eluting with CH_2Cl_2/MeOH = 20/1. The yellow solid was then dissolved in CH_2Cl_2 and precipitated with hexane under an argon atmosphere as it slowly decomposes in the air. The light yellow solid was collected by fractions in 27% yield (99 mg). ^{1}H NMR (500 MHz, $CDCl_3$, ppm): δ 8.43 (dd, J = 5, 2 Hz, 1H), 7.78-7.73 (m, 1H), 7.64 (dd, J = 7, 2 Hz, 1H), 7.48-7.42 (m, 2H), 7.24-7.19 (m, 1H), 7.03 (s, 2H), 6.97 (dd, J = 7, 5 Hz, 1H), 2.94 (sept, J = 7 Hz, 1H), 2.43 (sept, J = 7 Hz, 2H), 1.31 (d, J = 7 Hz, 6H), 1.14 (d, J = 7 Hz, 6H), 1.13 (d, J = 7 Hz, 6H); ^{13}C{^{1}H}MR (125 MHz, $CDCl_3$, ppm): δ 172.7, 153.4, 151.9, 150.0, 149.5, 143.6 (br), 141.3, 137.2 (br), 135.2, 135.0, 133.8 (br), 130.5, 123.2, 120.8, 120.4, 36.2, 34.5, 24.9, 24.8, 24.2; ^{11}B{^{1}H} NMR (160 MHz, $CDCl_3$, ppm): δ 72.8 (br). HRMS (APCI$^+$): m/z calcd for $[C_{26}H_{30}BN+H]^+$: 368.2544; Found: 368.2535 $[M+H]^+$; Elem. Anal. Calcd (%) for $C_{26}H_{30}BN$: C, 85.01; H, 8.23; N, 3.81; Found: C, 83.20; H, 8.09; N, 3.93;

NMR spectra

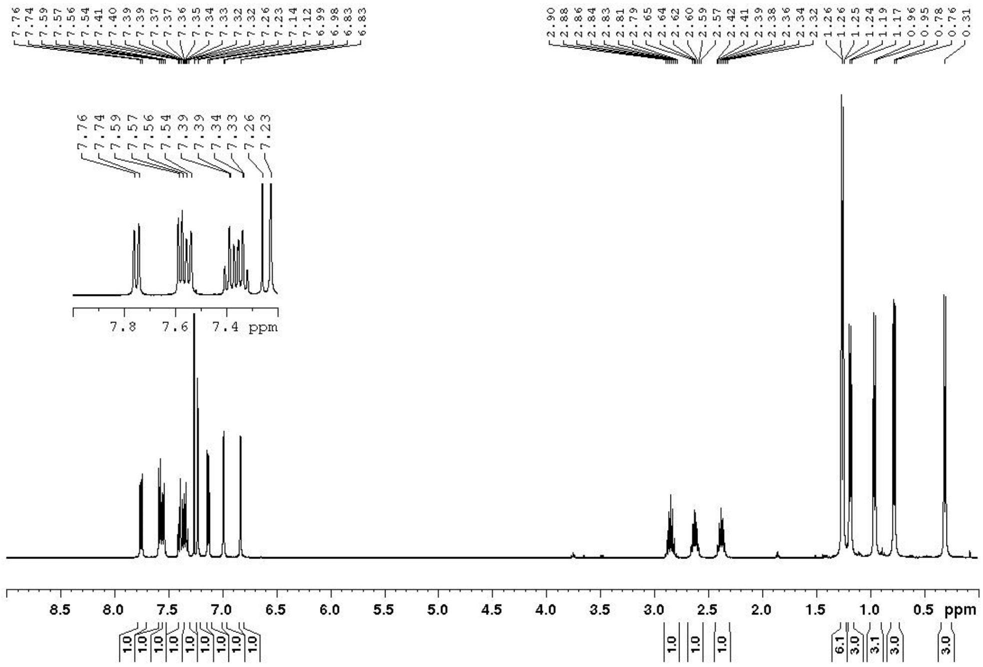

Figure S1. ^{1}H NMR spectrum of **[TipPBB1]$_4$** in $CDCl_3$ at 400 MHz.

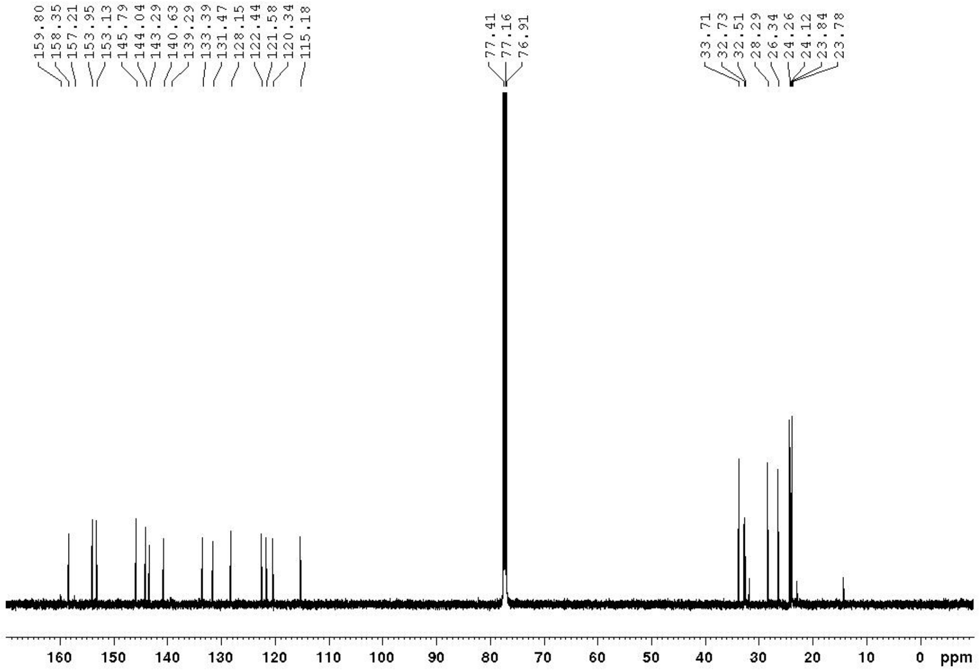

Figure S2. $^{13}C\{^{1}H\}$ NMR spectrum of **[TipPBB1]$_4$** in $CDCl_3$ at 125 MHz.

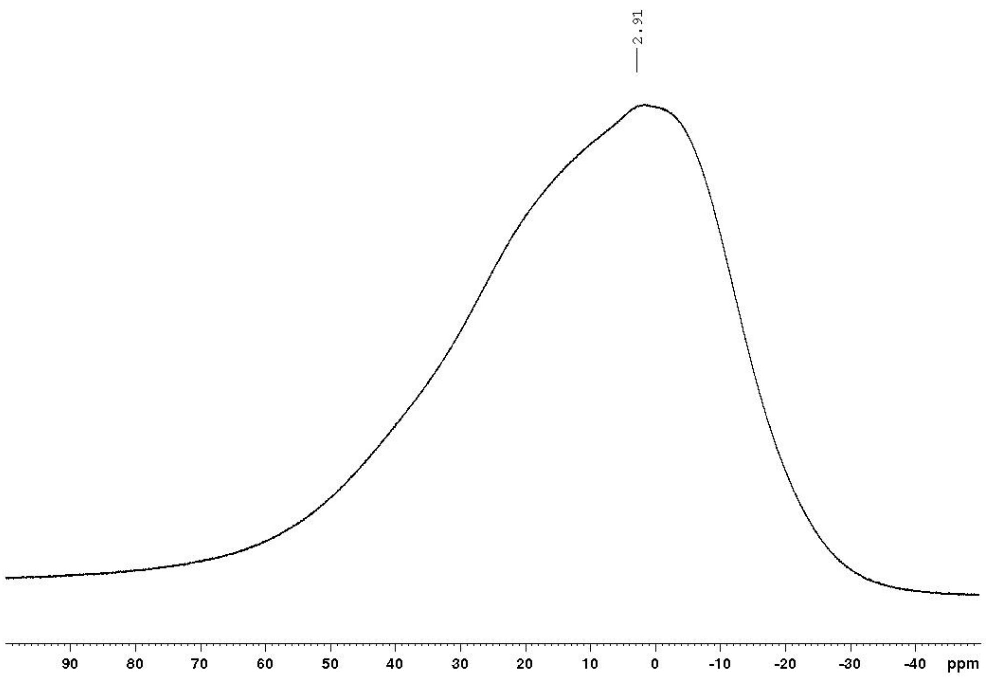

Figure S3. $^{11}B\{^1H\}$ NMR spectrum of **[TipPBB1]$_4$** in $CDCl_3$ at 160 MHz.

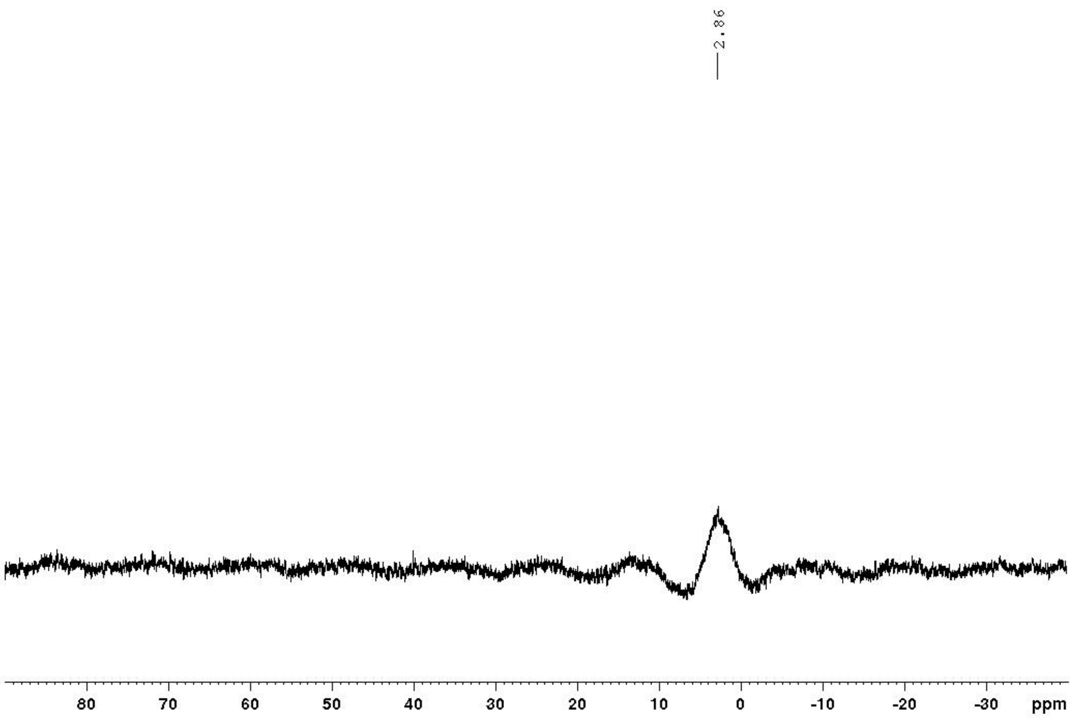

Figure S4. $^{11}B\{^1H\}$ NMR spectrum of **[TipPBB1]$_4$** in $CDCl_3$ at 160 MHz with background removed by linear backwards prediction.

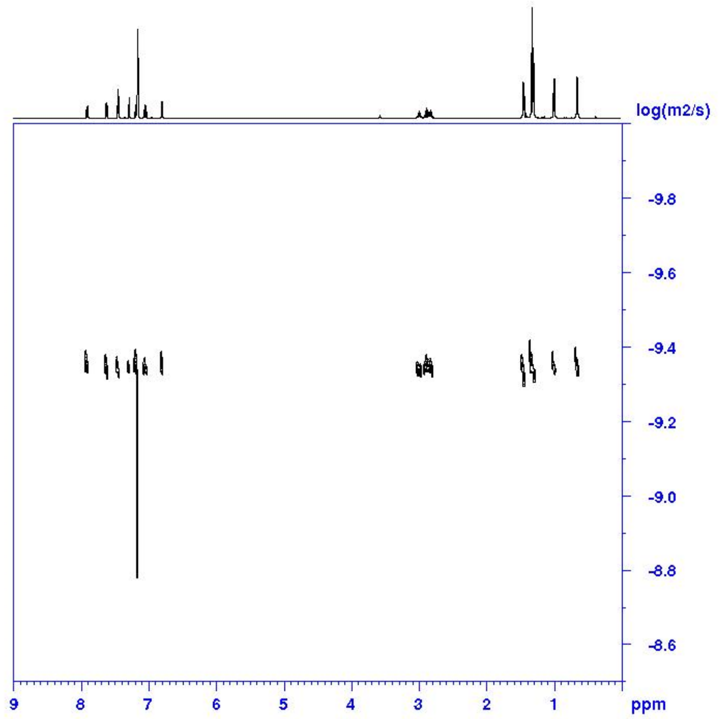

Figure S5. ^{1}H DOSY spectrum of **[TipPBB1]$_4$** in C_6D_6 at +25 °C.

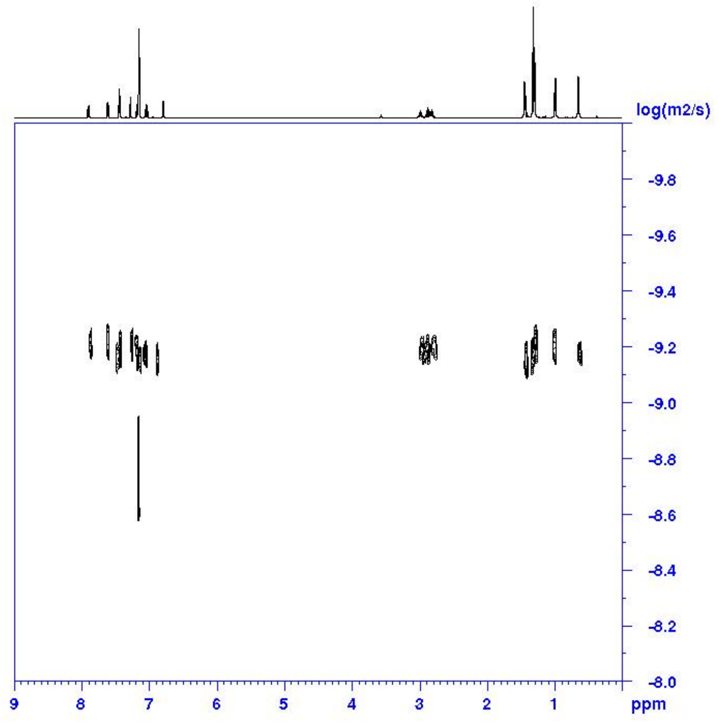

Figure S6. ^{1}H DOSY spectrum of **[TipPBB1]$_4$** in C_6D_6 at +50 °C.

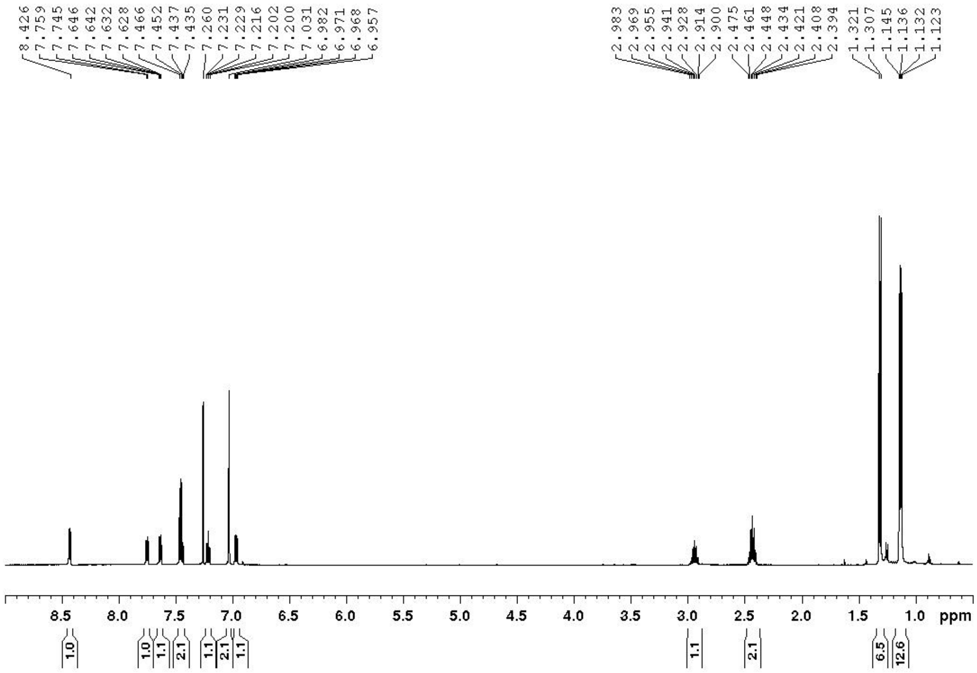

Figure S7. ^{1}H NMR spectrum of **TipPBB2** in $CDCl_3$ at 500 MHz.

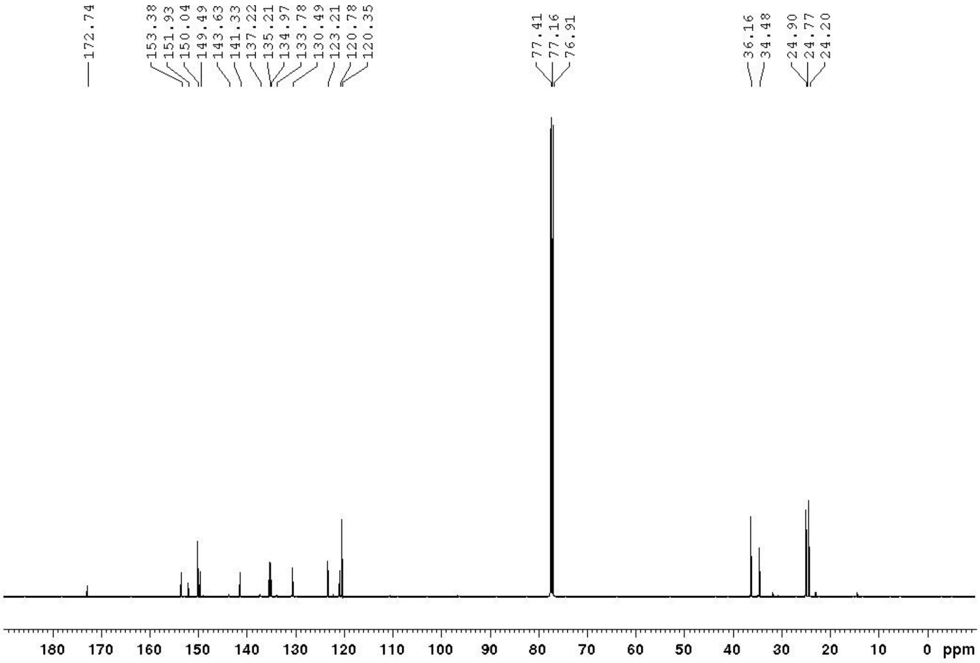

Figure S8. $^{13}C\{^{1}H\}$ NMR spectrum of **TipPBB2** in $CDCl_3$ at 125 MHz.

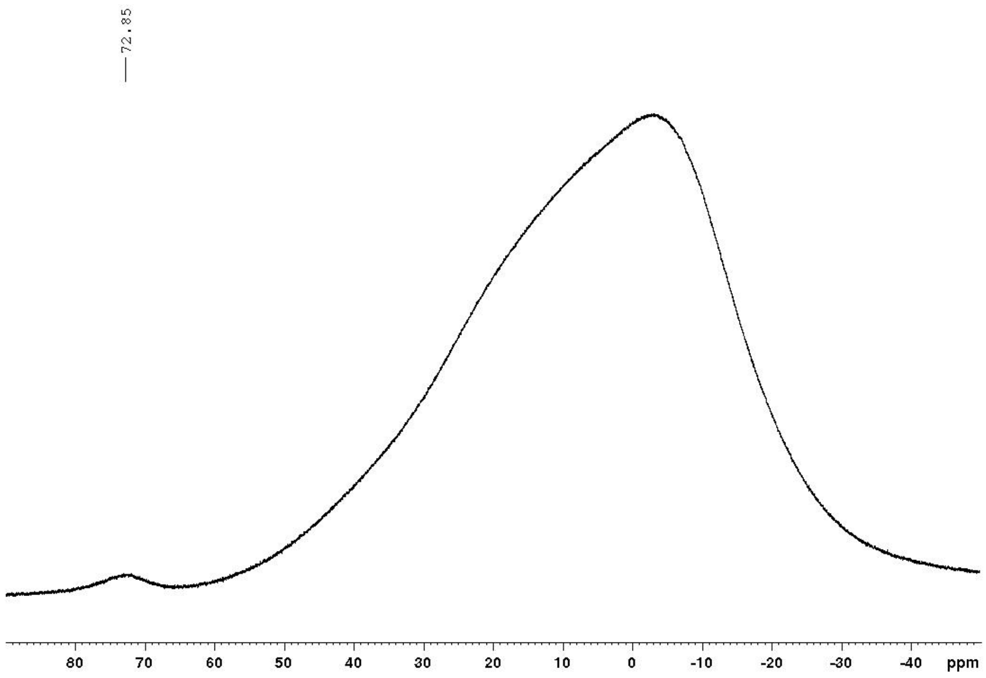

Figure S9. $^{11}B\{^{1}H\}$ NMR spectrum of **TipPBB2** in $CDCl_3$ at 160MHz.

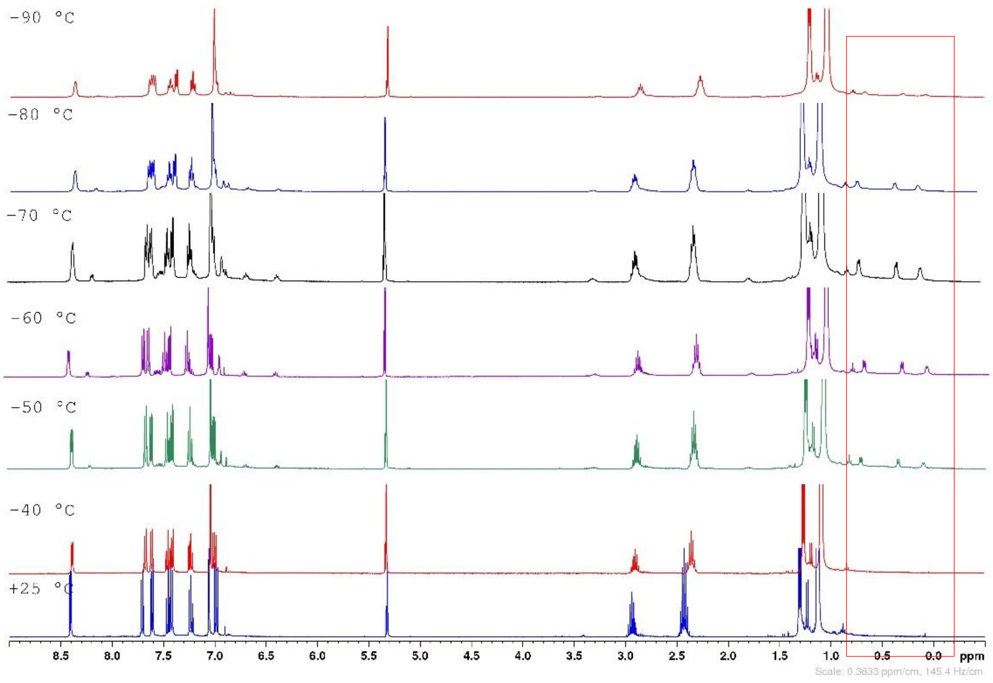

Figure S10. Low temperature ^{1}H NMR spectra of **TipPBB2** in CD_2Cl_2 at 400 MHz.

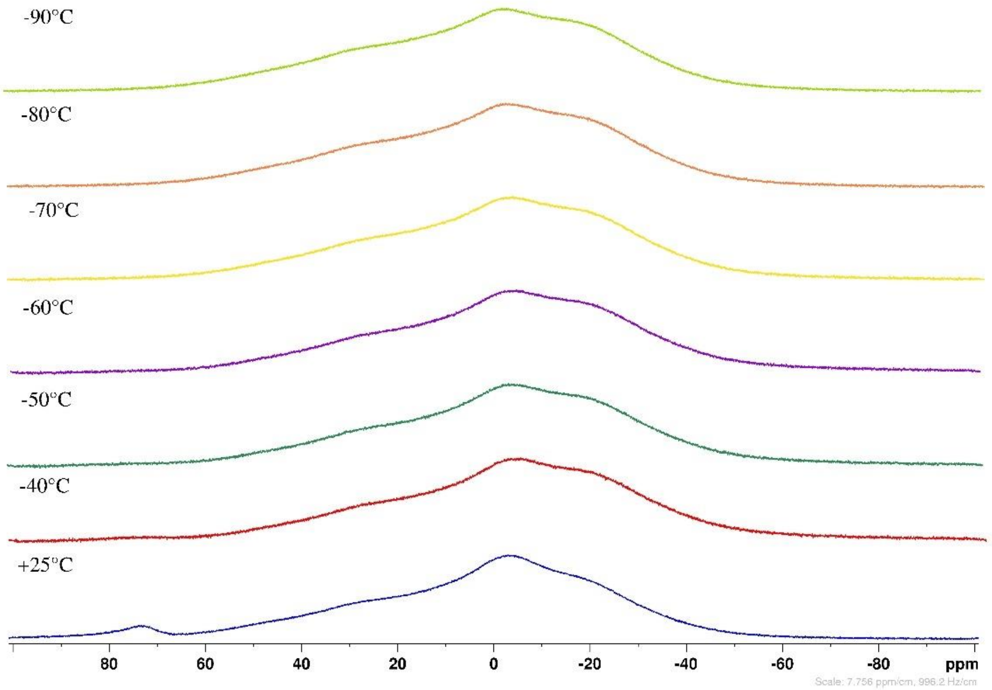

Figure S11. Low temperature $^{11}B\{^{1}H\}$ NMR spectra of **TipPBB2** in CD_2Cl_2 at 128MHz.

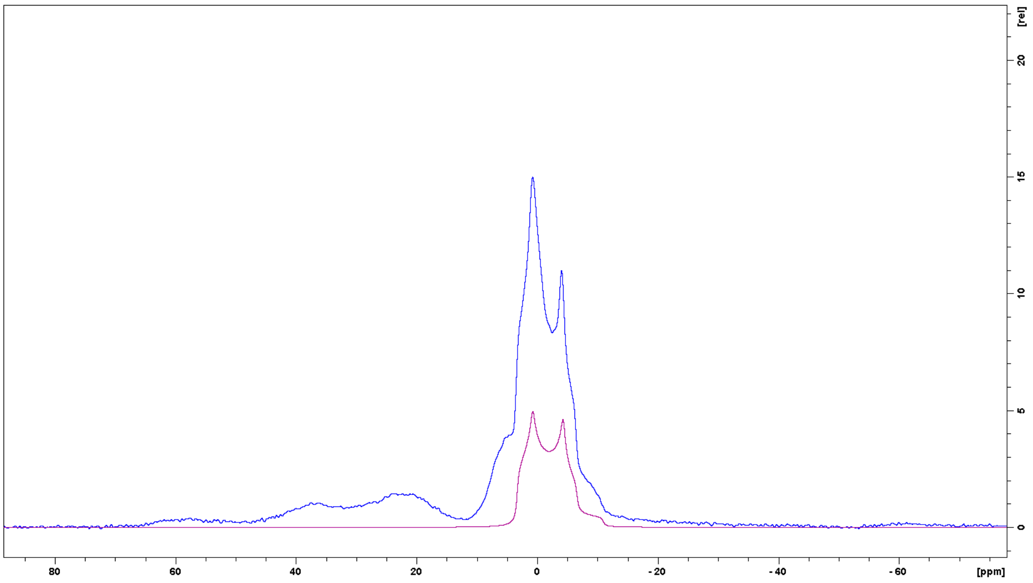

Figure S12. $^{11}B\{^{1}H\}$-RSHE/MAS (rotor synchronized Hahn-echo/ magic angle spinning) solid-state NMR spectrum (128 MHz, 293 K) of **TipPBB2** (top, blue) and simulated spectrum (bottom, violet).

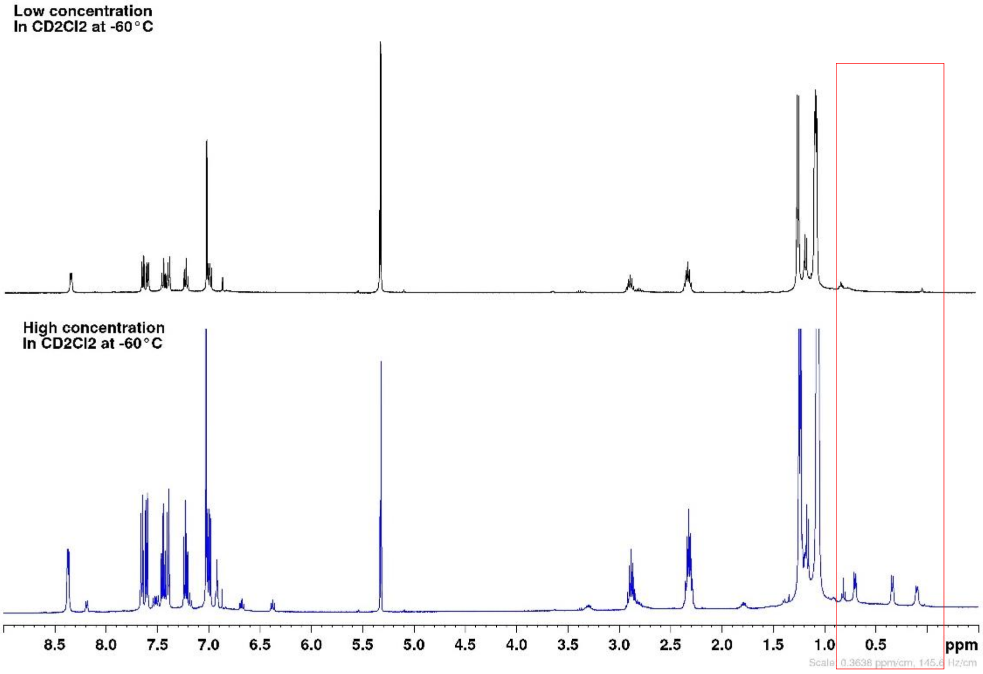

Figure S13. ^{1}H NMR spectra of **TipPBB2** in CD_2Cl_2 at –60 ºC at 400 MHz. The concentration of the sample of the bottom spectrum is ca. 4 times as concentrated as the sample of the top spectrum. No new peaks (red box) were observed at lower concentration which rules out the conformers as being responsible for the news peaks, at the same time, suggestes a concentration dependent equilibrium.

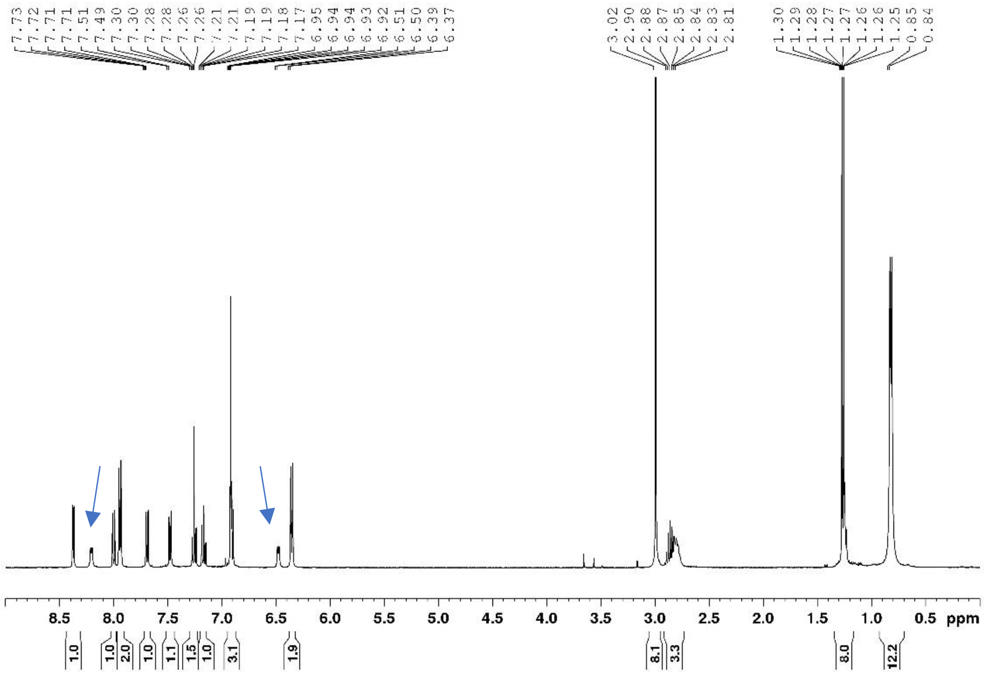

Figure S14. ^{1}H NMR spectrum of **TipPBB2** + DMAP in $CDCl_3$ at 400 MHz. The peaks marked with arrows are excess of DMAP.

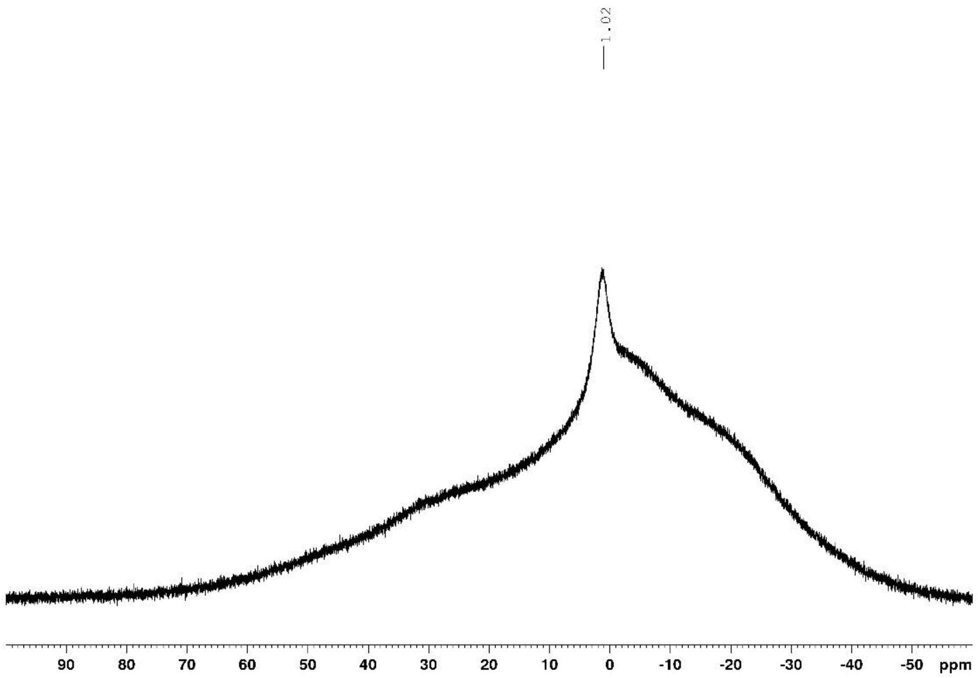

Figure S15. $^{11}B\{^1H\}$ NMR spectrum of **TipPBB2** + DMAP in $CDCl_3$ at 400 MHz.

Single-crystal X-ray diffraction

Table S1. Single-crystal X-ray diffraction data and structure refinements of **[TipPBB1]$_4$**.

Data	**[TipPBB1]$_4$**
CCDC number	2013331
Empirical formula	$C_{104}H_{120}B_4N_4 \cdot 5.115(C_6D_6)$
Formula weight / g·mol^{-1}	1868.79
T / K	100(2)
Radiation, λ / Å,	Cu-K$_\alpha$ 1.54184
Crystal color, habit	Colorless block
Crystal size / mm^3	0.357 × 0.353 × 0.153
Crystal system	Triclinic
Space group	$P\,\bar{1}$
Unit cell dimensions	
a (Å)	14.53964(16)
b (Å)	17.2926(2)
c (Å)	26.1078(3)
α (°)	103.9023(10)
β (°)	96.1424(9)
γ (°)	114.6288(11)
Volume (Å^3)	5630.42(11)
Z	2
ρ_{calc} / g·cm^{-3}	1.102
μ / mm^{-1}	0.464
F(000)	2014
θ range / °	2.94 to 77.53°
Reflections collected	118762
Unique reflections	23515
Parameters / restraints	1457 / 469 /
Goodness-of-fit on F^2	1.043
R_1 [I>2σ(I)]	0.0457
wR^2	0.1197
Max./min. residual electron density (e·Å^{-3})	0.328 / –0.283

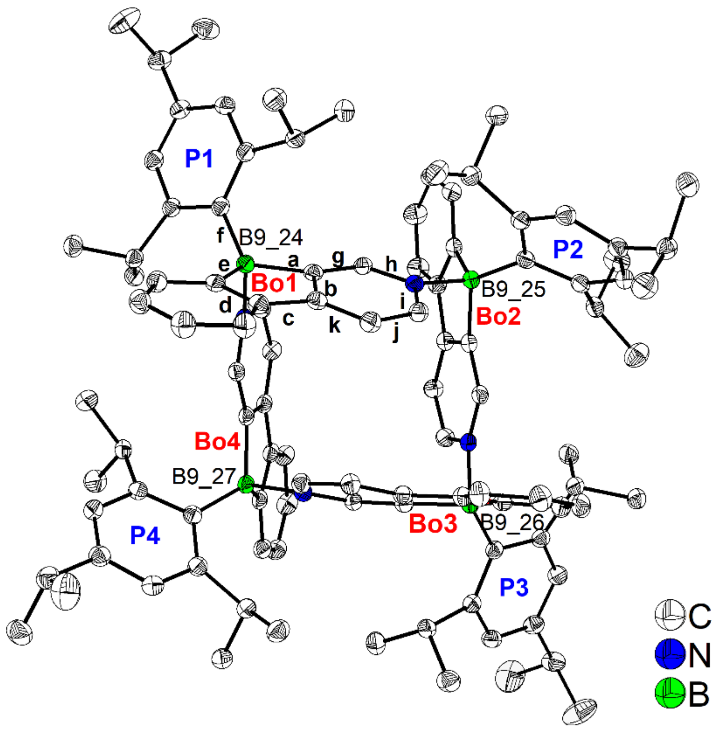

Figure S16. Molecular structure of **[TipPBB1]₄** in the solid state at 100 K. Atomic displacement ellipsoids are drawn at the 50% probability level, and H atoms as well as C_6D_6 solvent molecules are omitted for clarity. 'Bo' and 'P' denote the planes of the phenylpyridyl-fused borole and the Tip phenyl groups, respectively. Selected B–C and C–C bond distances are labelled with letters.

Table S2. Selected bond lengths (Å) and angles (°) (according to Figure S16) of **[TipPBB1]$_4$**.

	Bo1	Bo2	Bo3	Bo4
**g**: C10–C1 (Bo)	1.381(2)	1.379 (2)	1.380(2)	1.383(2)
**h**: C1–N2 (Bo)	1.357(2)	1.359(2)	1.358(2)	1.361(2)
**i**: N2–C3 (Bo)	1.351(2)	1.350(2)	1.352(2)	1.347(2)
**j**: C3–C4 (Bo)	1.378(2)	1.383(2)	1.381(2)	1.385(2)
**k**: C4–C11 (Bo)	1.392(2)	1.395(2)	1.391(2)	1.396(2)
$\angle C10_{Bo}–B–C13_{Bo}$	97.49(9)	98.00(9)	97.24(9)	97.80(9)
$\angle C10_{Bo}–B–C1_{Tip}$	123.90(10)	119.58(10)	124.46(10)	119.20(9)
$\angle C13_{Bo}–B–C1_{Tip}$	116.16(10)	121.69(10)	115.32(10)	119.67(10)
$\angle C1_{Tip}–B–N$	111.50(9)	108.33(9)	110.48(9)	109.10(9)

Photophysical properties

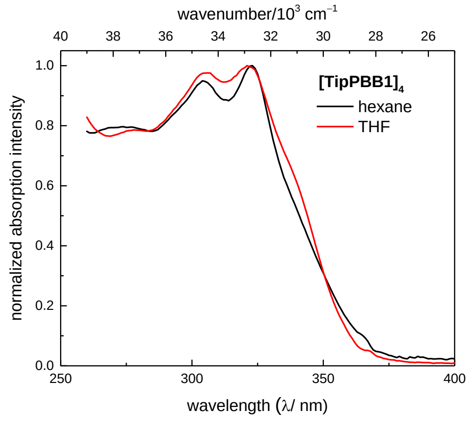

Figure S17. UV-vis absorption spectra of **[TipPBB1]$_4$** in hexane and THF.

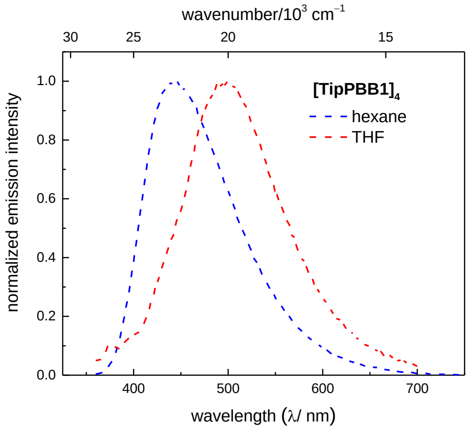

Figure S18. Emission spectra of **[TipPBB1]$_4$** in hexane and THF.

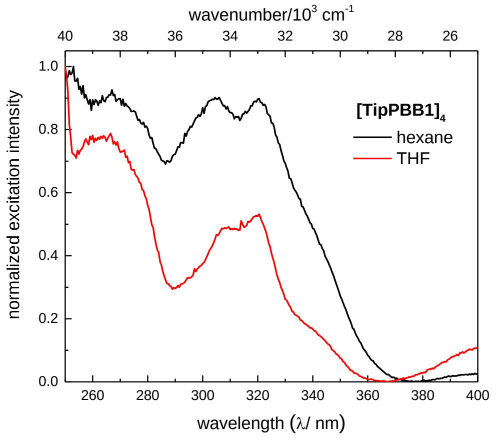

Figure S19. Excitation spectra of **[TipPBB1]$_4$** in hexane and THF.

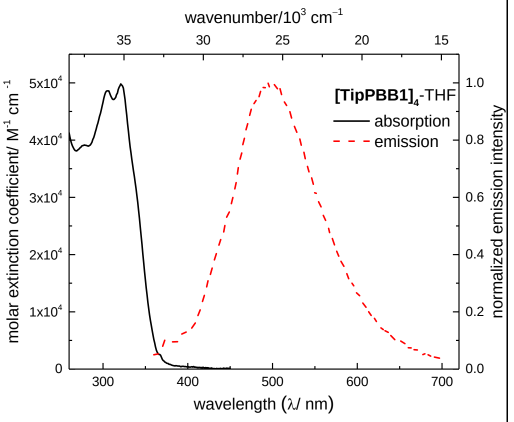

Figure S20. UV-vis absorption and emission spectra of **[TipPBB1]$_4$** in THF.

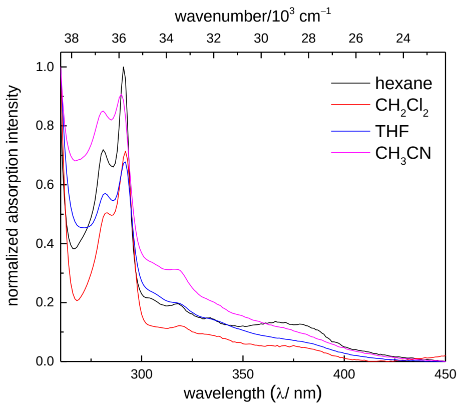

Figure S21. UV-vis absorption spectra of **TipPBB2** in different solvents.

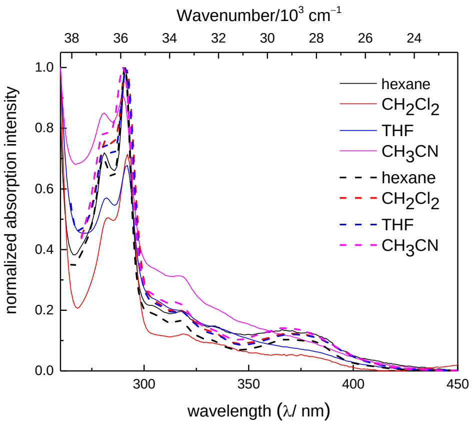

Figure S22. UV-vis absorption (solid line) and excitation spectra (dashed line) of **TipPBB2** in different solvents.

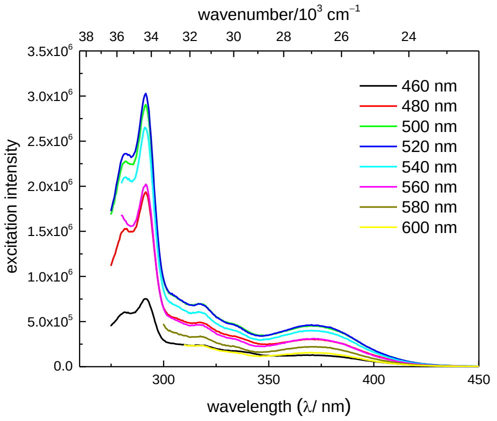

Figure S23. Excitation spectra of **TipPBB2** in CH_2Cl_2 at different emission wavelengths.

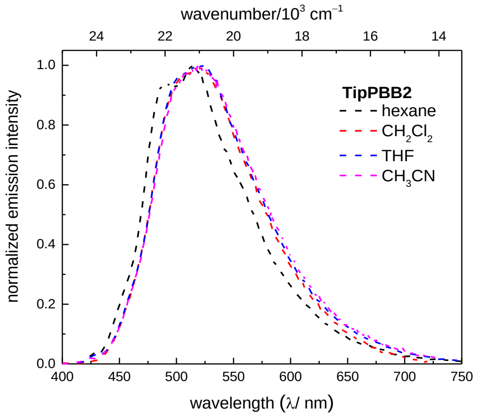

Figure S24. Emission spectra of **TipPBB2** in different solvents.

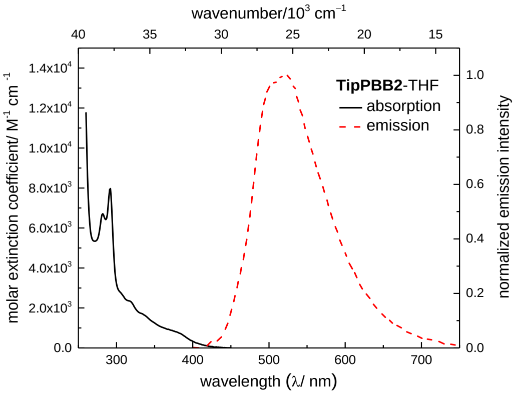

Figure S25. UV-vis absorption and emission spectra of **TipPBB2** in THF.

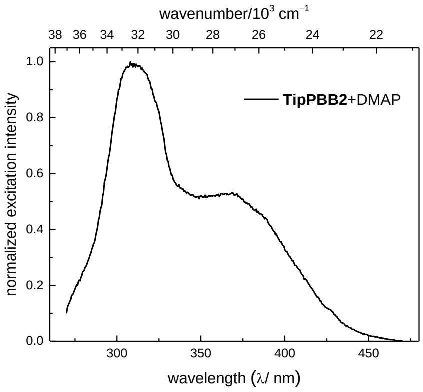

Figure S26. Excitation spectrum of **TipPBB2** + DMAP in THF.

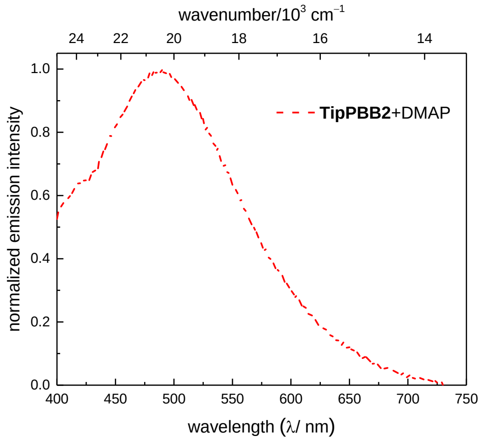

Figure S27. Emission spectrum of **TipPBB2** + DMAP in THF.

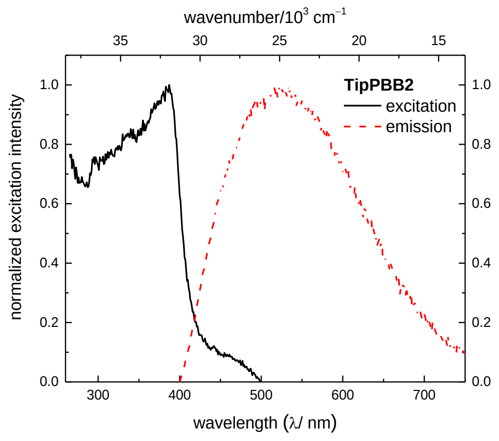

Figure S28. Excitation and emission spectra of **TipPBB2** in the solid state.

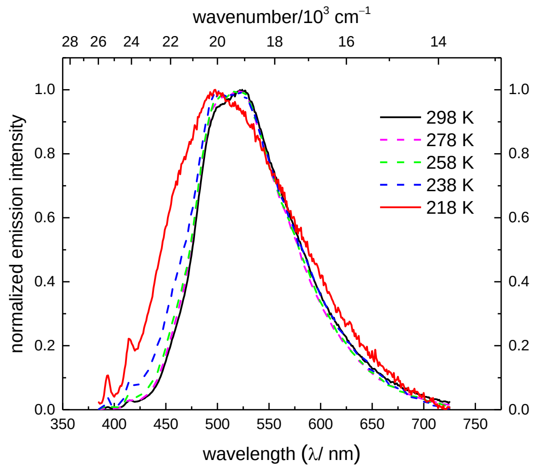

Figure S29. Normalized emission spectra of **Figure 4**.

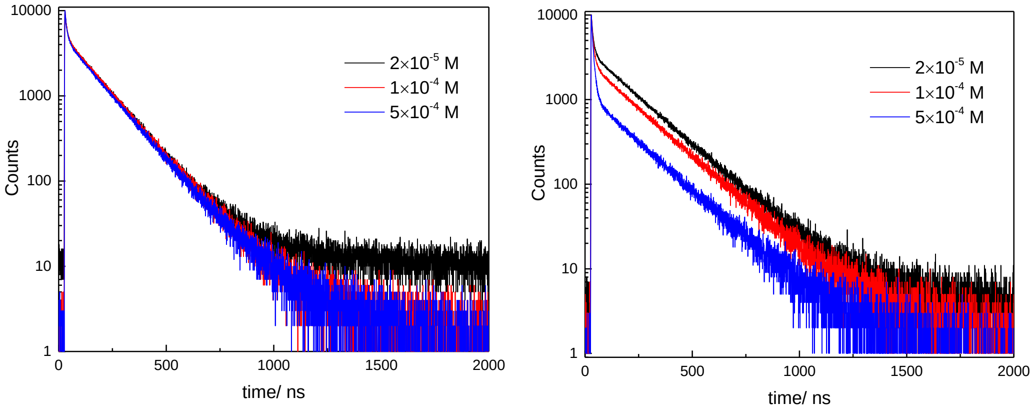

Figure S30. Lifetime measurements of **TipPBB2** at different concentrations at 298 K (left) and 213 K (right), measured in CH_2Cl_2.

Table S3. Lifetimes (ns) and corresponding relative percentages (in brackets) of **TipPBB2** at different concentrations at 298 K and 213 K, measured in CH_2Cl_2. τ_{LT} and τ_{ST} are the long and the short lifetimes, respectively.

Concentration	298 K		213 K	
(M)	τ_{LT} (rel.%)	τ_{ST} (rel.%)	τ_{LT} (rel.%)	τ_{LT} (rel.%)
2×10^{-5}	145 (93.4%)	7.3 (6.6%)	191 (91.0%)	8.4 (9.0%)
1×10^{-4}	146 (93.4%)	7.5 (6.6%)	188 (86.7%)	8.4 (13.3%)
5×10^{-4}	144 (93.0%)	7.3 (7.0%)	183 (69.3%)	8.5 (30.7%)

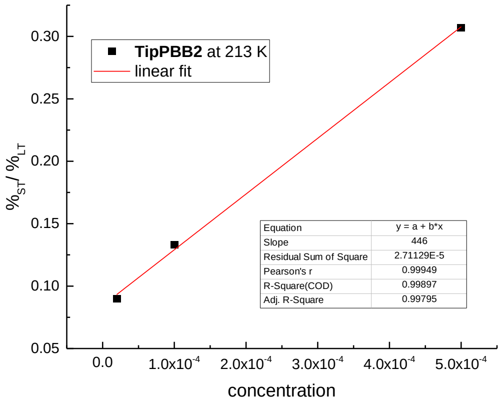

Figure S31. Plot of $\%_{ST}/\%_{LT}$ ($\%_{ST}$ and $\%_{LT}$ are the relative percentages of the long lifetime and the short lifetime components, respectively) vs. concentration (M) of **TipPBB2** in CH_2Cl_2 at 213 K.

Table S4. Lifetimes of **TipPBB2** at different temperatures and pre-exponential fitting parameters of τ_{LT} and τ_{ST}, measured in 2-MeTHF at λ_{em} 520 nm.

T. (K)	B_{ST}	τ_{ST} (ns)	B_{LT}	τ_{LT} (ns)
288	0.16	6.852	0.113	154.8
268	0.178	6.918	0.097	166.3
248	0.206	7.067	0.071	175.5
228	0.226	7.351	0.047	181.9
208	0.246	7.857	0.025	184.8
198	0.262	8.483	0.005	168.7
188	0.262	8.483	0.003	160.1
178	0.262	8.651	0.001	112.1
168	0.0022	5.275	0.076	14.35

The $\%_{LT}/\%_{ST}$ can be calculated by equation (1) at different temperatures from Table S4.[14]

$$\frac{\%_{LT}}{\%_{ST}} = \frac{\tau_{LT} * B_{LT}}{\tau_{ST} * B_{ST}} \quad (1)$$

$\%_{LT}$ is the relative value of the long lifetime component and $\%_{ST}$ is the relative value of the short lifetime component.

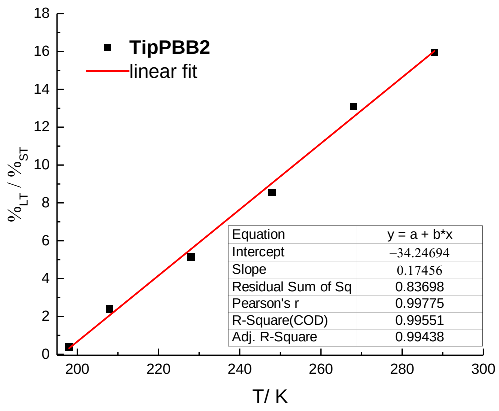

Figure S32. A plot of $\%_{LT}/\%_{ST}$ ($\%_{LT}$ and $\%_{ST}$ are the relative percentages of the long lifetime and the short lifetime components, respectively) vs. T (K) of **TipPBB2** in 2-MeTHF.

Cyclic voltammetry

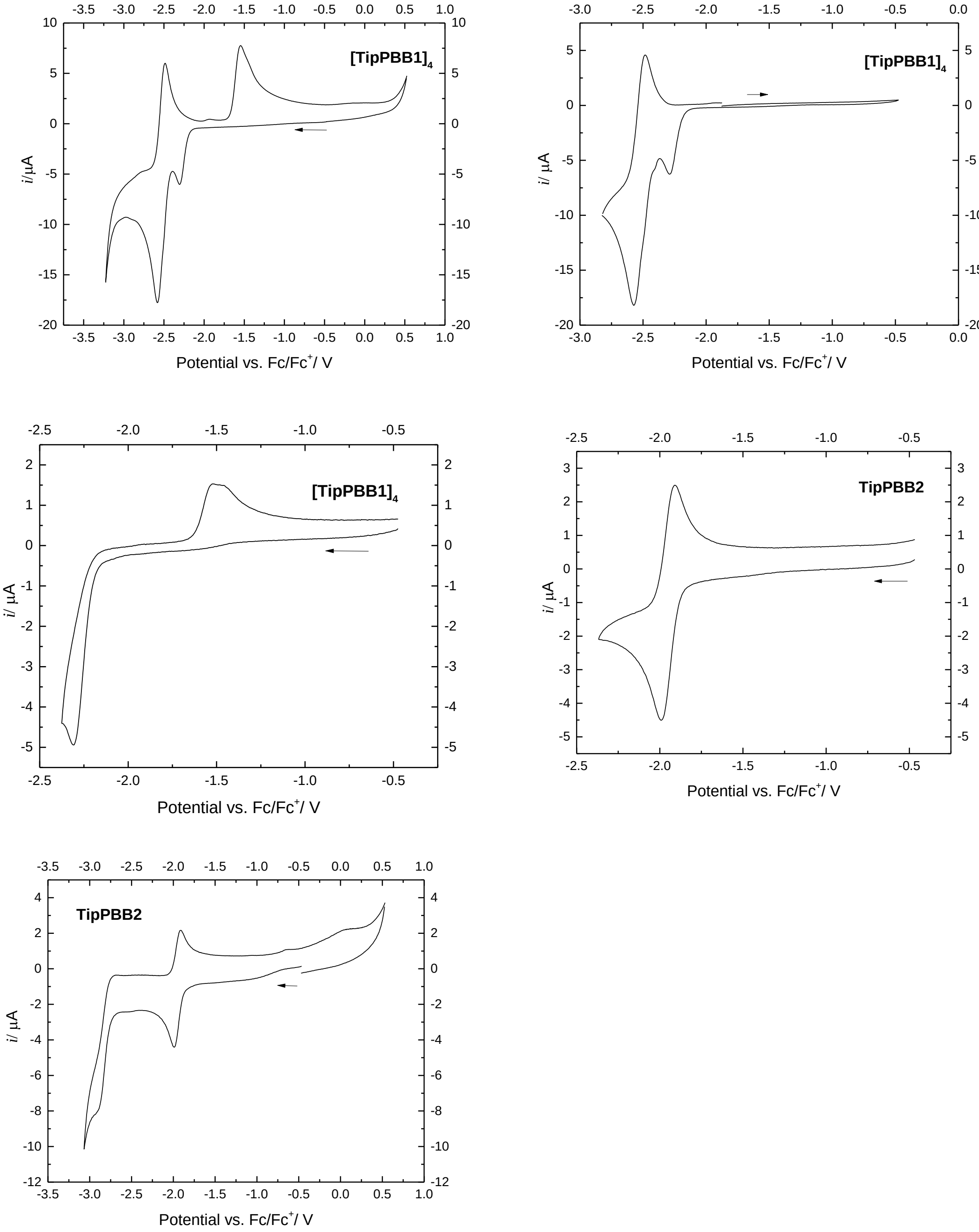

Figure S33. Cyclic voltammograms measured in THF in the presence of $TBAPF_6$ (0.1 M) as the electrolyte, scan rates of 250 mV s^{-1} with potentials given vs. the ferrocene/ferrocenium (Fc/Fc+) couple.

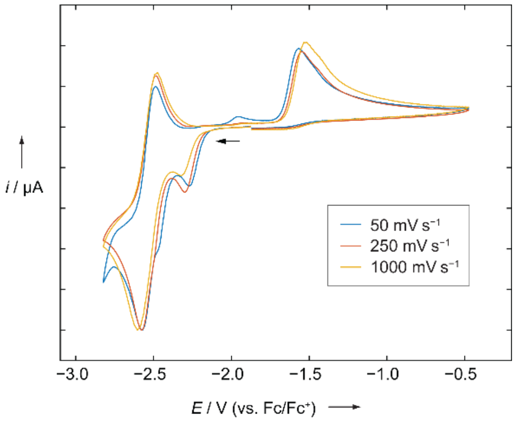

Figure S34. Overlay of cyclic voltammograms of **[TipPBB1]$_4$** taken at various scan rates. The currents are normalized.

TD-DFT Calculations

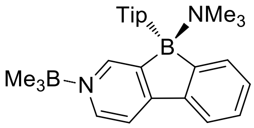

((BMe_3)TipPBB1(NMe_3))

Calculated absorption spectrum	Orbital	Energy [eV]
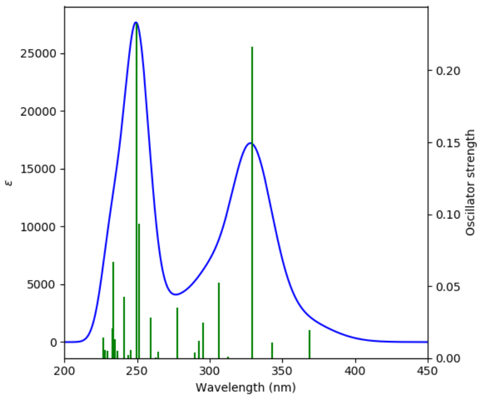	L+4	–0.34
	L+3	–0.46
	L+2	–0.58
	L+1	–1.37
	LUMO	–2.07
	HOMO	–6.05
	H-1	–622
	H-2	–6.35
	H-3	–6.70
TD-DFT B3LYP/6-31+G(d), gas phase	H-4	–6.85

Orbitals relevant to the $S_1 \leftarrow S_0$ transition	Other relevant orbitals	
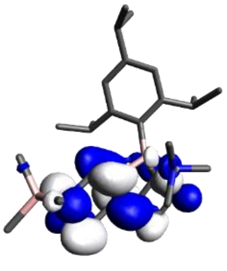 **LUMO**	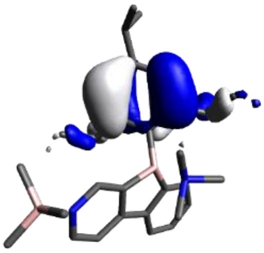 **HOMO-1**	
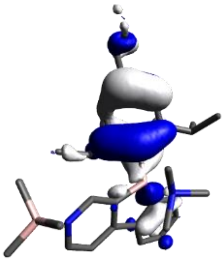 **HOMO**	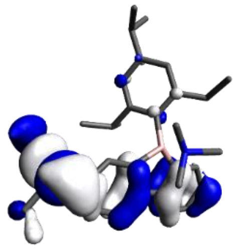 **HOMO-2**	

Table S4: Lowest energy singlet electronic transitions of **(BMe$_3$)TipPBB1(NMe$_3$)** (TD-DFT B3LYP/6-31+G(d), gas phase).

State	E [eV]	λ [nm]	*f*	Symmetry	Major contributions	Λ
**1**	3.36	368.51	0.0195	A	HOMO->LUMO (99%)	0.31
**2**	3.62	342.76	0.0108	A	H-1->LUMO (99%)	0.20
**3**	3.77	329.13	0.2167	A	H-2->LUMO (96%)	0.63
**4**	3.96	312.75	0.0012	A	H-3->LUMO (99%)	0.15
**5**	4.04	306.62	0.0522	A	HOMO->L+1 (95%)	0.37
**6**	4.19	295.71	0.0244	A	H-4->LUMO (65%), H-2->L+1 (17%)	0.60
**7**	4.24	292.48	0.0121	A	H-4->LUMO (27%), H-2->L+1 (33%), H-1->L+1 (26%)	0.47
**8**	4.28	289.48	0.0039	A	H-2->L+1 (23%), H-1->L+1 (67%)	0.31
**9**	4.47	277.50	0.0353	A	H-5->LUMO (64%), H-2->L+1 (10%)	0.53
**10**	4.69	264.40	0.0043	A	H-1->L+2 (41%), H-1->L+4 (10%), HOMO->L+5 (29%)	0.60
**11**	4.72	262.86	0.0002	A	H-3->L+1 (96%)	0.11
**12**	4.78	259.32	0.0281	A	H-6->LUMO (47%), H-4->L+1 (34%), H-2->L+1 (11%)	0.62
**13**	4.93	251.26	0.0934	A	H-6->LUMO (11%), H-4->L+1 (17%), HOMO->L+2 (48%)	0.65
**14**	4.96	249.78	0.2328	A	H-6->LUMO (11%), H-5->LUMO (10%), H-4->L+1 (33%), HOMO->L+2 (29%)	0.61
**15**	5.05	245.47	0.0057	A	H-7->LUMO (80%)	0.47
**16**	5.08	243.85	0.0022	A	HOMO->L+3 (95%)	0.23
**17**	5.14	241.02	0.0427	A	HOMO->L+4 (67%)	0.63
**18**	5.24	236.64	0.0053	A	H-1->L+2 (10%), H-1->L+3 (74%)	0.26
**19**	5.24	236.45	0.0019	A	H-8->LUMO (40%), H-2->L+2 (38%)	0.52
**20**	5.28	234.95	0.0133	A	H-1->L+2 (20%), H-1->L+3 (17%), H-1->L+4 (50%)	0.44
**21**	5.30	233.86	0.0666	A	H-5->L+1 (74%)	0.63
**22**	5.32	232.96	0.0209	A	H-8->LUMO (31%), H-2->L+2 (42%)	0.50
**23**	5.40	229.42	0.0053	A	H-2->L+3 (74%), HOMO->L+6 (14%)	0.20
**24**	5.43	228.13	0.0057	A	H-6->L+1 (14%), H-2->L+4 (11%), HOMO->L+5 (12%), HOMO->L+8 (23%), HOMO->L+9 (14%)	0.49
**25**	5.47	226.82	0.0144	A	H-2->L+3 (12%), HOMO->L+6 (45%), HOMO->L+8 (25%)	0.27

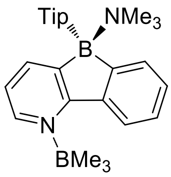

((BMe$_3$)TipPBB2(NMe$_3$))

Calculated absorption spectrum

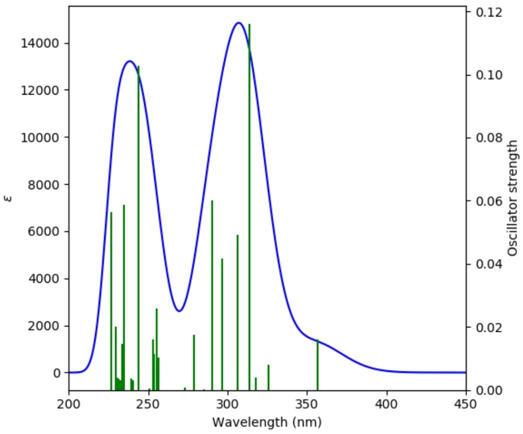

TD-DFT B3LYP/6-31+G(d), gas phase

Orbital	Energy [eV]
L+4	–0.21
L+3	–0.38
L+2	–0.59
L+1	–1.32
LUMO	–1.91
HOMO	–6.02
H-1	–6.25
H-2	–6.39
H-3	–6.55
H-4	–6.77

Orbitals relevant to the $S_1 \leftarrow S_0$ transition

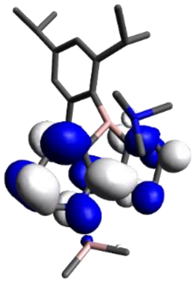

LUMO

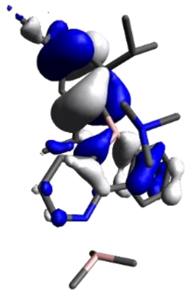

HOMO

Other relevant orbitals

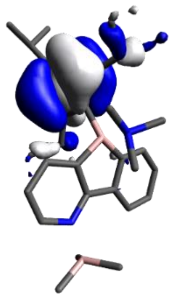

HOMO-1

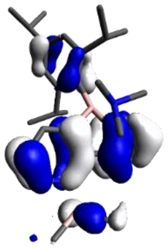

HOMO-2

Table S5: Lowest energy singlet electronic transition of **(BMe_3)TipPBB2(NMe_3)** (TD-DFT B3LYP/6-31+G(d), gas phase).

State	E [eV]	λ [nm]	*f*	Symmetry	Major contributions	Λ
**1**	3.47	356.87	0.016	A	HOMO->LUMO (98%)	0.36
**2**	3.81	325.70	0.008	A	H-1->LUMO (99%)	0.20
**3**	3.90	317.99	0.0039	A	H-3->LUMO (97%)	0.18
**4**	3.95	313.59	0.1161	A	H-2->LUMO (74%), HOMO->L+1 (16%)	0.64
**5**	4.05	306.45	0.0492	A	H-2->LUMO (14%), HOMO->L+1 (80%)	0.43
**6**	4.18	296.70	0.0418	A	H-4->LUMO (75%), H-2->L+1 (11%)	0.45
**7**	4.27	290.48	0.0602	A	H-5->LUMO (72%), H-4->LUMO (10%)	0.50
**8**	4.35	284.92	0.0003	A	H-1->L+1 (97%)	0.20
**9**	4.44	279.05	0.0174	A	H-2->L+1 (80%)	0.60
**10**	4.54	273.31	0.0008	A	H-3->L+1 (97%)	0.17
**11**	4.83	256.64	0.0102	A	H-1->L+4 (18%), HOMO->L+2 (16%), HOMO->L+3 (19%), HOMO->L+7 (12%)	0.53
**12**	4.86	255.18	0.0257	A	H-6->LUMO (18%), H-4->L+1 (68%)	0.48
**13**	4.88	253.91	0.0113	A	H-7->LUMO (25%), H-6->LUMO (47%), H-4->L+1 (23%)	0.56
**14**	4.89	253.40	0.0161	A	H-5->L+1 (75%)	0.47
**15**	4.94	250.77	0.0006	A	HOMO->L+2 (81%)	0.26
**16**	5.08	244.26	0.1027	A	HOMO->L+3 (62%)	0.55
**17**	5.15	240.58	0.003	A	H-1->L+2 (92%)	0.20
**18**	5.17	239.65	0.0038	A	H-7->LUMO (48%), H-6->LUMO (24%)	0.57
**19**	5.28	234.76	0.0587	A	H-2->L+2 (33%), HOMO->L+4 (24%), HOMO->L+5 (11%)	0.49
**20**	5.30	233.75	0.0147	A	H-2->L+2 (45%), H-1->L+3 (15%), HOMO->L+4 (14%), HOMO->L+6 (13%)	0.40
**21**	5.33	232.55	0.0031	A	H-8->LUMO (57%)	0.51
**22**	5.35	231.58	0.0037	A	H-1->L+3 (30%), HOMO->L+4 (15%), HOMO->L+5 (18%), HOMO->L+7 (15%)	0.53
**23**	5.39	230.09	0.004	A	H-2->L+2 (12%), HOMO->L+5 (14%), HOMO->L+6 (64%)	0.32
**24**	5.40	229.81	0.0201	A	H-1->L+3 (29%), HOMO->L+5 (23%), HOMO->L+7 (13%)	0.51
**25**	5.46	226.90	0.0563	A	H-6->L+1 (40%), H-2->L+3 (36%)	0.64

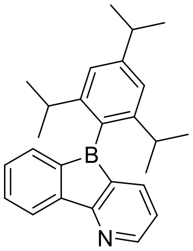

(**TipPBB2**)

Calculated absorption spectrum	Orbital	Energy [eV]
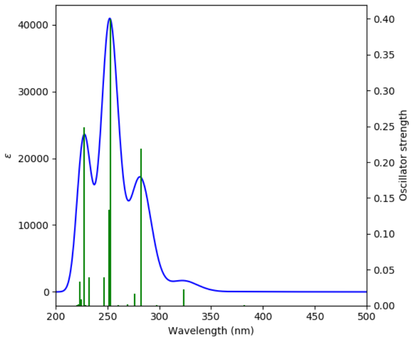 TD-DFT B3LYP/6-31+G(d), gas phase	L+4	–0.21
	L+3	–0.32
	L+2	–0.38
	L+1	–1.46
	LUMO	–2.53
	HOMO	–6.28
	H-1	–6.30
	H-2	–6.44
	H-3	–7.07
	H-4	–7.13

Orbitals relevant to the $S_1 \leftarrow S_0$ transition	Other relevant orbitals	
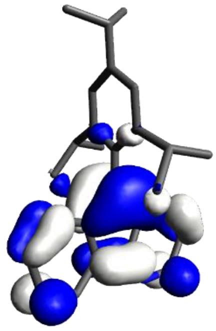 **LUMO**	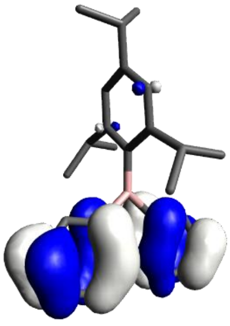 **HOMO-1**	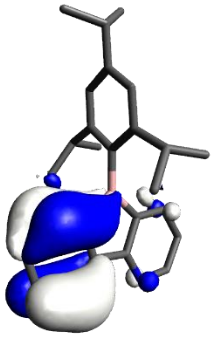 **HOMO-3**
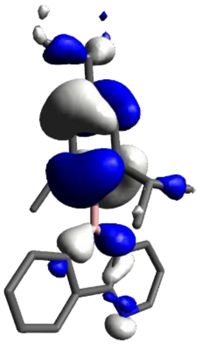 **HOMO**	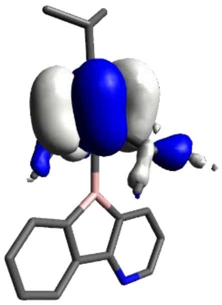 **HOMO-2**	

Table S6: Lowest energy singlet electronic transitions of **TipPBB2** (TD-DFT B3LYP/6-31+G(d), gas phase).

State	E [eV]	λ [nm]	*f*	Major contributions	Λ
**1**	2.92	423.94	0.0	HOMO->LUMO (98%)	0.27
**2**	2.95	419.96	0.0002	H-1->LUMO (98%)	0.65
**3**	3.25	381.98	0.0004	H-2->LUMO (99%)	0.21
**4**	3.59	345.24	0.0001	H-4->LUMO (97%)	0.33
**5**	3.84	323.30	0.0229	H-3->LUMO (74%), H-1->L+1 (22%)	0.61
**6**	4.17	297.62	0.0005	H-4->L+1 (10%), HOMO->L+1 (88%)	0.21
**7**	4.40	282.08	0.2188	H-5->LUMO (20%), H-3->LUMO (17%), H-1->L+1 (47%)	0.66
**8**	4.48	276.46	0.0166	H-2->L+1 (94%)	0.18
**9**	4.60	269.62	0.0019	H-4->L+1 (86%), HOMO->L+1 (10%)	0.42
**10**	4.76	260.55	0.0008	H-7->LUMO (87%)	0.37
**11**	4.90	252.93	0.3998	H-5->LUMO (65%), H-1->L+1 (18%)	0.69
**12**	4.93	251.26	0.1333	H-6->LUMO (44%), H-3->L+1 (35%)	0.63
**13**	4.98	249.12	0.0002	H-2->L+3 (45%), HOMO->L+4 (46%)	0.66
**14**	5.03	246.40	0.0393	H-6->LUMO (35%), H-3->L+1 (43%), H-1->L+2 (13%)	0.63
**15**	5.22	237.50	0.0002	H-9->LUMO (59%), HOMO->L+2 (15%)	0.36
**16**	5.27	235.32	0.0002	H-9->LUMO (12%), HOMO->L+2 (78%)	0.29
**17**	5.34	232.09	0.0392	H-8->LUMO (73%), H-1->L+2 (11%)	0.37
**18**	5.42	228.92	0.0008	H-1->L+3 (94%)	0.27
**19**	5.45	227.57	0.2487	H-2->L+4 (14%), H-1->L+2 (15%), HOMO->L+3 (51%)	0.79
**20**	5.52	224.77	0.0066	HOMO->L+5 (82%)	0.26
**21**	5.52	224.56	0.0083	H-2->L+2 (56%), H-1->L+2 (12%), HOMO->L+5 (12%)	0.33
**22**	5.56	223.18	0.0334	H-2->L+2 (34%), H-1->L+2 (11%), H-1->L+4 (17%)	0.41
**23**	5.59	221.98	0.0015	H-1->L+4 (75%)	0.34
**24**	5.61	220.87	0.0001	H-10->LUMO (34%), H-1->L+5 (40%)	0.28
**25**	5.62	220.71	0.0012	H-10->LUMO (45%), H-1->L+5 (34%)	0.30

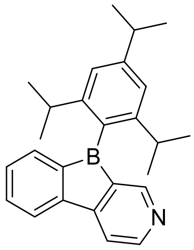

(TipPBB1)

Calculated absorption spectrum	Orbital	Energy [eV]
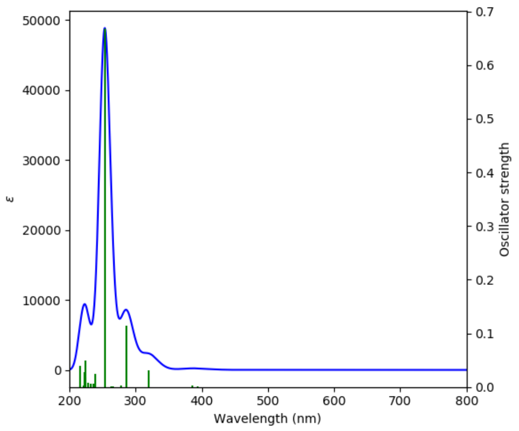	L+4	–0.17
	L+3	–0.27
	L+2	–0.34
	L+1	–1.46
	LUMO	–2.58
	HOMO	–6.29
	H-1	–6.43
	H-2	–6.59
	H-3	–7.15
TD-DFT B3LYP/6-31+G(d), gas phase	H-4	–7.17

Orbitals relevant to the $S_1 \leftarrow S_0$ transition	Other relevant orbitals	
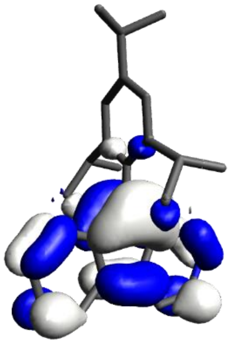 **LUMO**	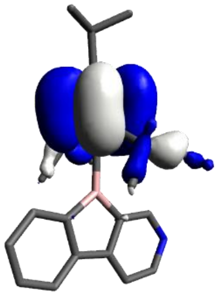 **HOMO-1**	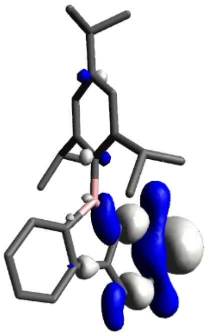 **HOMO-3**
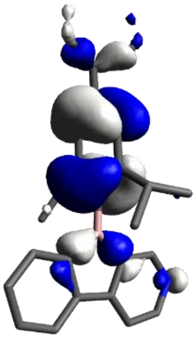 **HOMO**	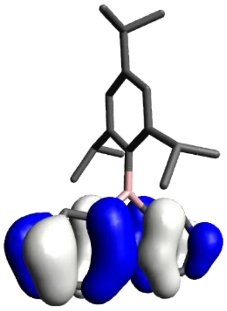 **HOMO-2**	

Table S7: Lowest energy singlet electronic transitions of **TipPBB1** (TD-DFT B3LYP/6-31+G(d), gas phase).

State	E [eV]	λ [nm]	*f*	Symmetry	Major contributions	Λ
**1**	2.90	427.05	0.0001	A	HOMO->LUMO (98%)	0.25
**2**	3.15	393.85	0.0008	A	H-2->LUMO (60%), H-1->LUMO (39%)	0.49
**3**	3.21	386.27	0.0024	A	H-2->LUMO (39%), H-1->LUMO (60%)	0.39
**4**	3.63	341.57	0.0003	A	H-3->LUMO (97%)	0.33
**5**	3.88	319.74	0.0308	A	H-4->LUMO (80%), H-2->L+1 (18%)	0.65
**6**	4.20	295.23	0.0001	A	HOMO->L+1 (95%)	0.16
**7**	4.34	285.92	0.1143	A	H-5->LUMO (89%)	0.71
**8**	4.45	278.32	0.0029	A	H-1->L+1 (97%)	0.15
**9**	4.67	265.59	0.0014	A	H-3->L+1 (89%)	0.38
**10**	4.70	263.55	0.0007	A	H-6->LUMO (89%)	0.36
**11**	4.89	253.40	0.6673	A	H-4->LUMO (18%), H-2->L+1 (75%)	0.68
**12**	5.02	247.19	0.0002	A	H-1->L+3 (46%), HOMO->L+4 (44%)	0.65
**13**	5.12	242.08	0.0001	A	H-10->LUMO (13%), H-9->LUMO (72%)	0.40
**14**	5.18	239.43	0.0252	A	H-7->LUMO (44%), H-5->L+1 (23%), H-4->L+1 (19%)	0.63
**15**	5.26	235.84	0.0063	A	H-7->LUMO (19%), H-4->L+1 (71%)	0.71
**16**	5.33	232.48	0.0	A	HOMO->L+2 (90%)	0.34
**17**	5.34	232.03	0.0056	A	H-8->LUMO (73%), H-7->LUMO (15%)	0.61
**18**	5.44	227.89	0.0078	A	H-7->LUMO (12%), H-5->L+1 (33%), H-2->L+2 (27%)	0.70
**19**	5.52	224.54	0.0502	A	H-1->L+2 (10%), H-1->L+4 (16%), HOMO->L+3 (42%), HOMO->L+5 (17%)	0.64
**20**	5.54	223.74	0.0181	A	HOMO->L+5 (68%)	0.30
**21**	5.58	222.36	0.0284	A	H-1->L+2 (78%)	0.41
**22**	5.60	221.51	0.0023	A	H-11->LUMO (16%), H-10->LUMO (60%), H-9->LUMO (20%)	0.41
**23**	5.65	219.38	0.0001	A	H-1->L+5 (82%), H-1->L+6 (14%)	0.23
**24**	5.73	216.40	0.003	A	H-2->L+3 (85%)	0.26
**25**	5.74	215.98	0.0391	A	H-11->LUMO (51%), H-10->LUMO (14%)	0.47

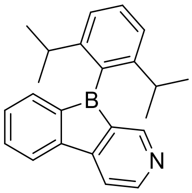

(**DipPBB1**)

Calculated absorption spectrum	Orbital	Energy [eV]
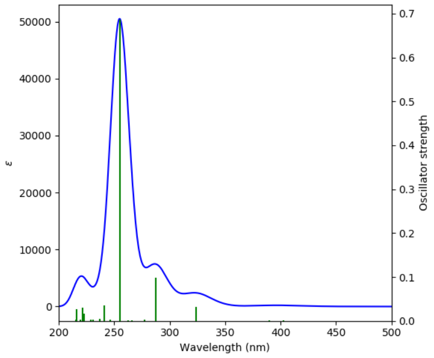	L+4	–0.25
	L+3	–0.38
	L+2	–0.41
	L+1	–1.51
	LUMO	–2.66
TD-DFT B3LYP/6-31+G(d), gas phase	HOMO	–6.48
	H-1	–6.50
	H-2	–6.59
	H-3	–7.16
	H-4	–7.20

Orbitals relevant to the $S_1 \leftarrow S_0$ transition	Other relevant orbitals	
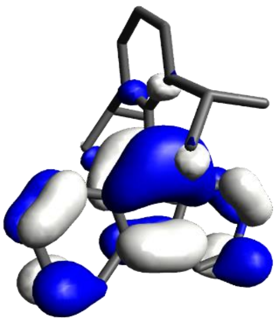 **LUMO**	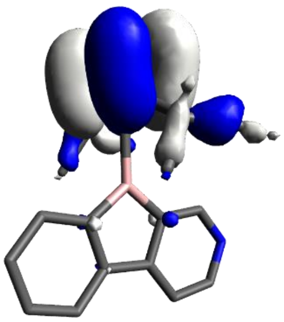 **HOMO**	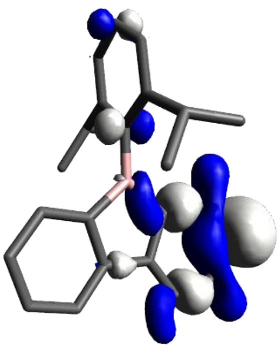 **HOMO-3**
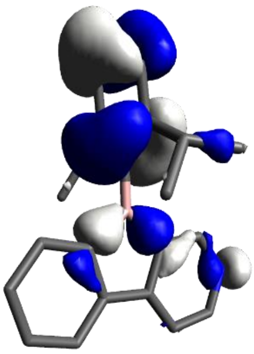 **HOMO-1**	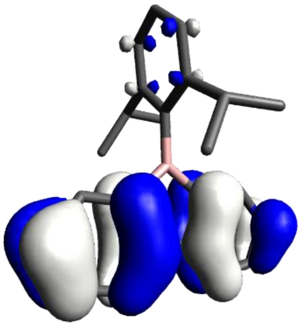 **HOMO-2**	

Table S8: Lowest energy singlet electronic transitions of **DipPBB1** (TD-DFT B3LYP/6-31+G(d), gas phase).

State	E [eV]	λ [nm]	*f*	Symmetry	Major contributions	Λ
**1**	3.01	412.55	0.0001	A	H-1->LUMO (98%)	0.28
**2**	3.08	402.27	0.0016	A	H-2->LUMO (78%), HOMO->LUMO (21%)	0.57
**3**	3.18	389.54	0.0015	A	H-2->LUMO (21%), HOMO->LUMO (79%)	0.33
**4**	3.58	346.69	0.0003	A	H-3->LUMO (97%)	0.33
**5**	3.83	323.57	0.0322	A	H-4->LUMO (80%), H-2->L+1 (18%)	0.65
**6**	4.31	287.77	0.0003	A	H-3->L+1 (11%), H-1->L+1 (87%)	0.20
**7**	4.31	287.40	0.0989	A	H-5->LUMO (89%)	0.72
**8**	4.47	277.54	0.0027	A	HOMO->L+1 (97%)	0.20
**9**	4.67	265.67	0.0011	A	H-3->L+1 (82%), H-1->L+1 (12%)	0.36
**10**	4.72	262.70	0.0007	A	H-6->LUMO (93%)	0.39
**11**	4.86	254.99	0.6867	A	H-4->LUMO (18%), H-2->L+1 (74%)	0.68
**12**	5.03	246.44	0.0023	A	H-1->L+4 (37%), HOMO->L+2 (55%)	0.64
**13**	5.14	241.12	0.0001	A	H-10->LUMO (11%), H-9->LUMO (83%)	0.45
**14**	5.15	240.92	0.0355	A	H-7->LUMO (60%), H-5->L+1 (20%), H-4->L+1 (12%)	0.70
**15**	5.23	237.05	0.0044	A	H-7->LUMO (13%), H-4->L+1 (77%)	0.75
**16**	5.37	231.00	0.0037	A	H-8->LUMO (75%), H-2->L+3 (13%)	0.47
**17**	5.42	228.86	0.0033	A	H-8->LUMO (15%), H-5->L+1 (40%), H-2->L+3 (19%)	0.68
**18**	5.46	226.96	0.0	A	H-1->L+3 (87%)	0.36
**19**	5.57	222.68	0.017	A	H-1->L+2 (35%), HOMO->L+3 (44%), HOMO->L+4 (12%)	0.61
**20**	5.59	221.67	0.0002	A	H-2->L+2 (94%)	0.26
**21**	5.60	221.41	0.0299	A	H-1->L+2 (21%), HOMO->L+3 (50%), HOMO->L+4 (24%)	0.60
**22**	5.65	219.31	0.0026	A	H-10->LUMO (85%)	0.35
**23**	5.70	217.36	0.0	A	HOMO->L+5 (75%), HOMO->L+6 (21%)	0.22
**24**	5.73	216.32	0.0265	A	H-11->LUMO (80%)	0.40
**25**	5.75	215.64	0.0021	A	H-1->L+5 (82%), H-1->L+6 (16%)	0.20

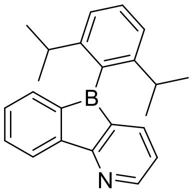

(**DipPBB2**)

Calculated absorption spectrum	Orbital	Energy [eV]
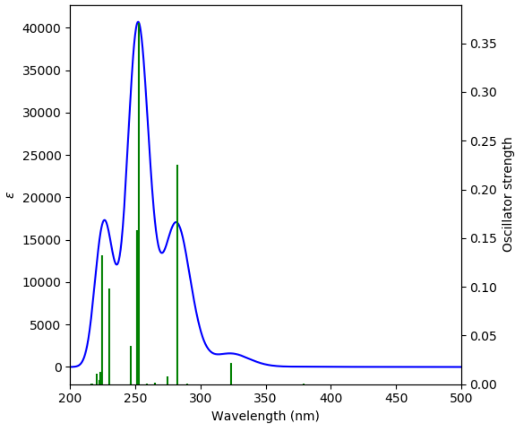	L+4	–0.26
	L+3	–0.41
	L+2	–0.41
	L+1	–1.48
	LUMO	–2.57
	HOMO	–6.33
	H-1	–6.49
	H-2	–6.50
	H-3	–7.10
TD-DFT B3LYP/6-31+G(d), gas phase	H-4	–7.18

Orbitals relevant to the $S_1 \leftarrow S_0$ transition	Other relevant orbitals	
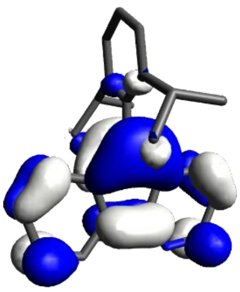 **LUMO**	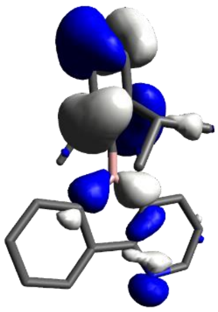 **HOMO-1**	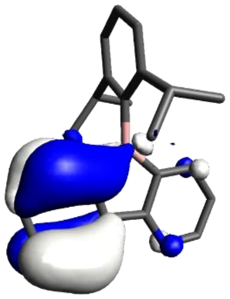 **HOMO-3**
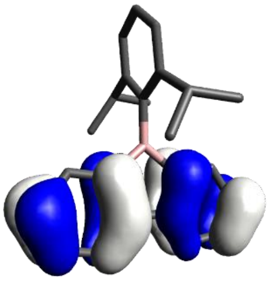 **HOMO**	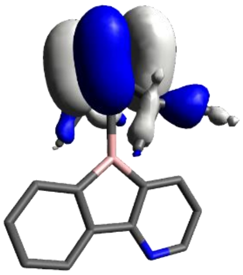 **HOMO-2**	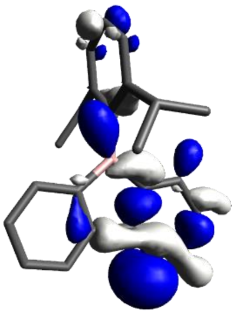 **HOMO-4**

Table S9: Lowest energy singlet electronic transitions of **DipPBB2** (TD-DFT B3LYP/6-31+G(d), gas phase).

State	E [eV]	λ [nm]	*f*	Symmetry	Major contributions	Λ
**1**	2.95	420.91	0.0001	A	HOMO->LUMO (99%)	0.65
**2**	3.07	404.46	0.0	A	H-1->LUMO (97%)	0.28
**3**	3.27	379.02	0.0004	A	H-2->LUMO (99%)	0.19
**4**	3.61	343.27	0.0	A	H-4->LUMO (97%)	0.33
**5**	3.83	323.63	0.0217	A	H-3->LUMO (74%), HOMO->L+1 (22%)	0.61
**6**	4.27	290.04	0.0009	A	H-4->L+1 (20%), H-1->L+1 (78%)	0.25
**7**	4.40	281.99	0.225	A	H-5->LUMO (21%), H-3->LUMO (17%), HOMO->L+1 (48%)	0.68
**8**	4.52	274.51	0.0084	A	H-2->L+1 (97%)	0.15
**9**	4.67	265.41	0.0014	A	H-4->L+1 (77%), H-1->L+1 (21%)	0.39
**10**	4.80	258.57	0.0009	A	H-7->LUMO (92%)	0.41
**11**	4.90	253.01	0.3712	A	H-5->LUMO (64%), HOMO->L+1 (17%)	0.69
**12**	4.93	251.38	0.158	A	H-6->LUMO (45%), H-3->L+1 (32%)	0.63
**13**	5.03	246.51	0.0389	A	H-6->LUMO (32%), H-3->L+1 (45%), HOMO->L+3 (14%)	0.63
**14**	5.03	246.39	0.002	A	H-2->L+2 (53%), H-1->L+4 (41%)	0.64
**15**	5.32	233.08	0.0001	A	H-9->LUMO (78%)	0.44
**16**	5.35	231.96	0.0	A	HOMO->L+2 (94%)	0.21
**17**	5.40	229.72	0.0981	A	H-8->LUMO (55%), HOMO->L+3 (26%)	0.49
**18**	5.41	229.22	0.0	A	H-1->L+3 (83%)	0.29
**19**	5.52	224.71	0.1327	A	H-8->LUMO (15%), H-2->L+4 (14%), H-1->L+2 (39%), HOMO->L+3 (14%)	0.69
**20**	5.55	223.34	0.0125	A	H-2->L+3 (16%), HOMO->L+4 (68%)	0.28
**21**	5.56	222.82	0.0046	A	H-2->L+3 (77%), HOMO->L+4 (20%)	0.23
**22**	5.63	220.35	0.0111	A	H-8->LUMO (21%), H-2->L+4 (21%), H-1->L+2 (22%), HOMO->L+3 (10%)	0.62
**23**	5.64	219.88	0.0001	A	HOMO->L+5 (87%)	0.20
**24**	5.71	217.22	0.0007	A	H-2->L+5 (85%)	0.24
**25**	5.73	216.30	0.0004	A	H-1->L+5 (92%)	0.24

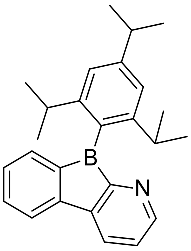

(**TipPBB3**)

Calculated absorption spectrum	Orbital	Energy [eV]
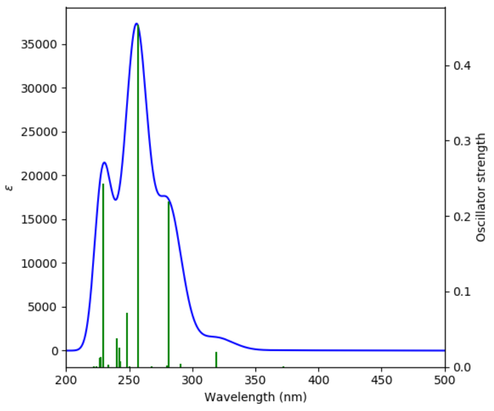	L+4	–0.10
	L+3	–0.18
	L+2	–0.41
	L+1	–1.50
	LUMO	–2.58
	HOMO	–6.10
TD-DFT B3LYP/6-31+G(d), gas phase	H-1	–6.25
	H-2	–6.32
	H-3	–6.95
	H-4	–7.18

Orbitals relevant to the $S_1 \leftarrow S_0$ transition	Other relevant orbitals	
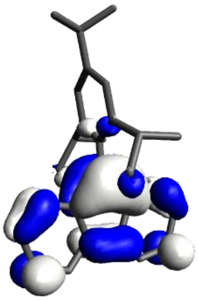 **LUMO**	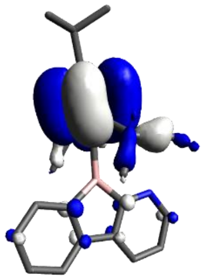 **HOMO-1**	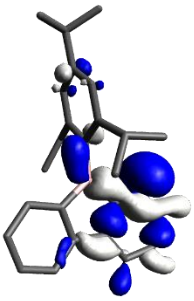 **HOMO-3**
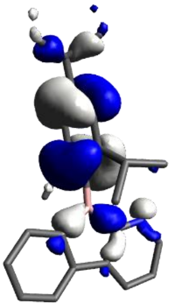 **HOMO**	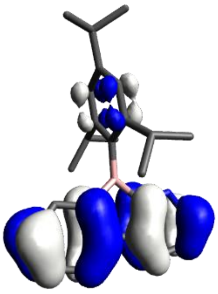 **HOMO-2**	

Table S10: Lowest energy singlet electronic transitions of **TipPBB3** (TD-DFT B3LYP/6-31+G(d), gas phase).

State	E [eV]	λ [nm]	*f*	Major contributions	Λ
**1**	2.68	461.78	0.0001	HOMO->LUMO (97%)	0.27
**2**	2.91	426.40	0.0001	H-2->LUMO (79%), H-1->LUMO (20%)	0.57
**3**	3.01	411.28	0.0002	H-2->LUMO (21%), H-1->LUMO (79%)	0.36
**4**	3.33	372.10	0.0005	H-3->LUMO (97%)	0.41
**5**	3.88	319.29	0.02	H-4->LUMO (72%), H-2->L+1	0.60
**6**	3.97	312.46	0.0002	HOMO->L+1 (91%)	0.18
**7**	4.26	290.85	0.0046	H-1->L+1 (94%)	0.25
**8**	4.41	281.23	0.2193	H-5->LUMO (28%), H-4->LUMO (17%), H-2->L+1 (43%)	0.68
**9**	4.42	280.29	0.0024	H-3->L+1 (88%)	0.39
**10**	4.63	267.99	0.0005	H-6->LUMO (89%)	0.35
**11**	4.83	256.88	0.4541	H-5->LUMO (61%), H-2->L+1 (24%)	0.68
**12**	4.96	250.21	0.0004	H-1->L+3 (43%), HOMO->L+5 (43%)	0.62
**13**	4.99	248.24	0.0717	H-7->LUMO (13%), H-4->L+1 (49%), H-2->L+2 (12%)	0.59
**14**	5.04	245.84	0.0	HOMO->L+2 (84%)	0.25
**15**	5.10	242.92	0.0072	H-9->LUMO (43%), H-7->LUMO (25%)	0.43
**16**	5.11	242.48	0.0251	H-9->LUMO (24%), H-7->LUMO (47%)	0.55
**17**	5.17	240.02	0.0376	H-8->LUMO (71%), H-4->L+1 (17%)	0.47
**18**	5.31	233.38	0.0029	H-1->L+2 (94%)	0.30
**19**	5.40	229.43	0.2431	H-4->L+1 (13%), H-2->L+2 (23%), HOMO->L+3 (38%)	0.74
**20**	5.45	227.70	0.0028	H-11->LUMO (59%), H-9->LUMO (17%)	0.39
**21**	5.45	227.65	0.0136	H-11->LUMO (11%), HOMO->L+4 (43%), HOMO->L+6 (15%)	0.35
**22**	5.46	227.01	0.0122	H-10->LUMO (19%), H-1->L+5 (11%), HOMO->L+3 (24%), HOMO->L+4 (21%)	0.53
**23**	5.54	224.00	0.0005	H-2->L+3 (23%), H-2->L+4 (53%), H-1->L+4 (14%)	0.28
**24**	5.58	222.39	0.0002	H-2->L+3 (67%), H-2->L+4 (13%), H-1->L+4 (11%)	0.30
**25**	5.59	221.89	0.0003	H-2->L+4 (21%), H-1->L+4 (39%), H-1->L+6 (31%)	0.25

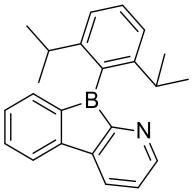

(**DipPBB3**)

<table>
<tr><th>Calculated absorption spectrum</th><th>Orbital</th><th>Energy [eV]</th></tr>
<tr><td rowspan="10">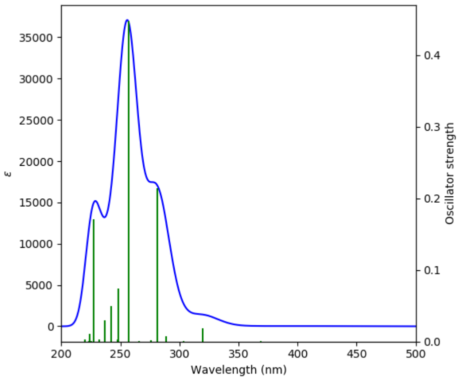
TD-DFT B3LYP/6-31+G(d), gas phase</td><td>L+4</td><td>–0.12</td></tr>
<tr><td>L+3</td><td>–0.26</td></tr>
<tr><td>L+2</td><td>–0.44</td></tr>
<tr><td>L+1</td><td>–1.52</td></tr>
<tr><td>LUMO</td><td>–2.62</td></tr>
<tr><td>HOMO</td><td>–6.30</td></tr>
<tr><td>H-1</td><td>–6.30</td></tr>
<tr><td>H-2</td><td>–6.35</td></tr>
<tr><td>H-3</td><td>–7.00</td></tr>
<tr><td>H-4</td><td>–7.21</td></tr>
<tr><td>Orbitals relevant to the $S_1 \leftarrow S_0$ transition</td><td colspan="2">Other relevant orbitals</td></tr>
<tr><td>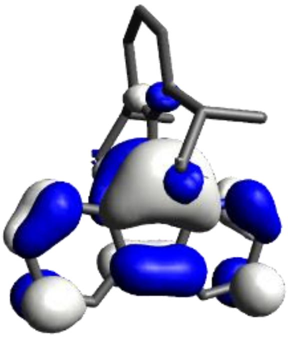
LUMO</td><td>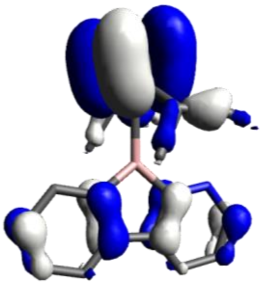
HOMO-1</td><td>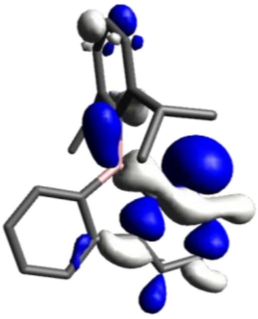
HOMO-3</td></tr>
<tr><td>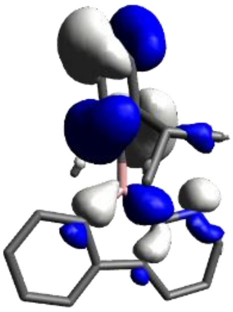
HOMO</td><td>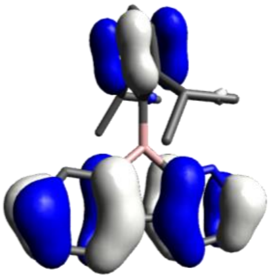
HOMO-2</td><td></td></tr>
</table>

Table S11: Lowest energy singlet electronic transitions of **DipPBB3** (TD-DFT B3LYP/6-31+G(d), gas phase).

State	E [eV]	λ [nm]	*f*	Symmetry	Major contributions	Λ
**1**	2.82	440.24	0.0002	A	HOMO->LUMO (97%)	0.30
**2**	2.90	427.28	0.0001	A	H-2->LUMO (66%), H-1->LUMO (33%)	0.54
**3**	3.04	408.27	0.0002	A	H-2->LUMO (33%), H-1->LUMO (67%)	0.46
**4**	3.36	369.00	0.0004	A	H-3->LUMO (96%)	0.40
**5**	3.88	319.58	0.0185	A	H-4->LUMO (72%), H-2->L+1 (18%)	0.59
**6**	4.08	303.70	0.0005	A	H-3->L+1 (16%), HOMO->L+1 (83%)	0.22
**7**	4.29	288.79	0.0075	A	H-2->L+1 (11%), H-1->L+1 (86%)	0.39
**8**	4.41	281.19	0.2145	A	H-5->LUMO (28%), H-4->LUMO (17%), H-2->L+1 (43%)	0.66
**9**	4.49	276.15	0.0015	A	H-3->L+1 (81%), HOMO->L+1 (16%)	0.36
**10**	4.66	265.79	0.0005	A	H-6->LUMO (93%)	0.39
**11**	4.83	256.94	0.4481	A	H-5->LUMO (61%), H-2->L+1 (21%)	0.66
**12**	5.00	248.20	0.0743	A	H-7->LUMO (21%), H-4->L+1 (48%), H-2->L+2 (10%)	0.59
**13**	5.01	247.48	0.0025	A	H-1->L+3 (47%), HOMO->L+5 (40%)	0.61
**14**	5.11	242.40	0.0498	A	H-7->LUMO (61%), H-4->L+1 (15%), H-2->L+2 (10%)	0.62
**15**	5.17	239.61	0.0	A	HOMO->L+2 (75%)	0.27
**16**	5.23	237.17	0.0297	A	H-8->LUMO (79%), H-4->L+1 (13%)	0.41
**17**	5.24	236.66	0.0002	A	H-12->LUMO (10%), H-11->LUMO (16%), H-9->LUMO (49%), HOMO->L+2 (18%)	0.34
**18**	5.34	232.06	0.003	A	H-2->L+2 (16%), H-1->L+2 (83%)	0.44
**19**	5.45	227.64	0.1712	A	H-4->L+1 (15%), H-2->L+2 (30%), HOMO->L+3 (16%)	0.63
**20**	5.50	225.47	0.0003	A	H-2->L+3 (77%), H-1->L+3 (18%)	0.43
**21**	5.53	224.17	0.0108	A	H-10->LUMO (22%), H-1->L+5 (19%), HOMO->L+3 (47%)	0.69
**22**	5.53	224.09	0.0044	A	H-11->LUMO (61%), H-9->LUMO (32%)	0.39
**23**	5.55	223.42	0.0003	A	H-2->L+4 (64%), H-1->L+4 (28%)	0.26
**24**	5.64	219.99	0.0	A	H-2->L+4 (19%), H-1->L+4 (39%), H-1->L+6 (29%)	0.24
**25**	5.64	219.78	0.0031	A	HOMO->L+4 (75%), HOMO->L+6 (20%)	0.19

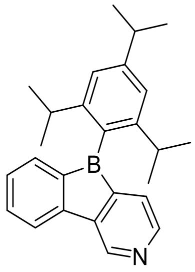

(**TipPBB4**)

<table>
<tr><td>Calculated absorption spectrum</td><td>Orbital</td><td>Energy [eV]</td></tr>
<tr><td rowspan="10">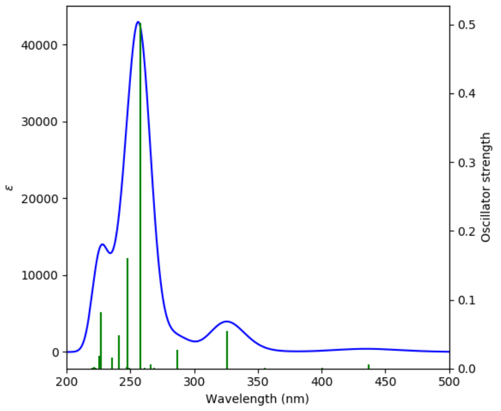

TD-DFT B3LYP/6-31+G(d), gas phase</td><td>L+4</td><td>–0.26</td></tr>
<tr><td>L+3</td><td>–0.38</td></tr>
<tr><td>L+2</td><td>–0.49</td></tr>
<tr><td>L+1</td><td>–1.32</td></tr>
<tr><td>LUMO</td><td>–2.73</td></tr>
<tr><td>HOMO</td><td>–6.35</td></tr>
<tr><td>H-1</td><td>–6.37</td></tr>
<tr><td>H-2</td><td>–6.48</td></tr>
<tr><td>H-3</td><td>–7.13</td></tr>
<tr><td>H-4</td><td>–7.19</td></tr>
<tr><td>Orbitals relevant to the $S_1 \leftarrow S_0$ transition</td><td colspan="2">Other relevant orbitals</td></tr>
<tr><td>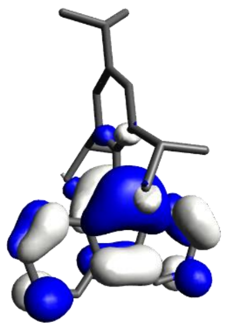
LUMO</td><td>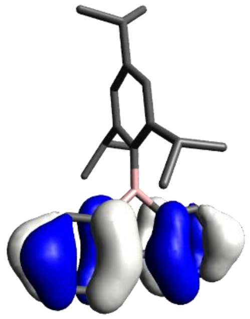
HOMO-1</td><td>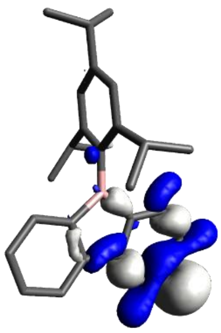
HOMO-3</td></tr>
<tr><td>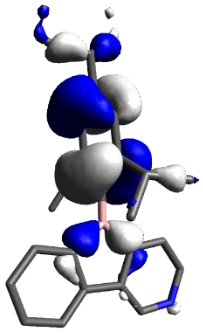
HOMO</td><td>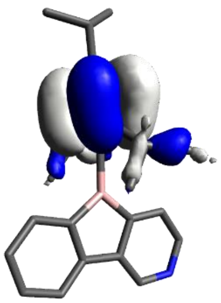
HOMO-2</td><td></td></tr>
</table>

Table S12: Lowest energy singlet electronic transitions of **TipPBB4** (TD-DFT B3LYP/6-31+G(d), gas phase).

State	E [eV]	λ [nm]	*f*	Major contributions	Λ
**1**	2.82	439.93	0.0001	HOMO->LUMO (98%)	0.26
**2**	2.84	436.78	0.0054	H-1->LUMO (99%)	0.64
**3**	3.10	400.36	0.0008	H-2->LUMO (99%)	0.17
**4**	3.49	355.69	0.0011	H-3->LUMO (98%)	0.39
**5**	3.81	325.61	0.0545	H-4->LUMO (84%), H-1->L+1 (13%)	0.63
**6**	4.33	286.42	0.0266	H-5->LUMO (86%)	0.70
**7**	4.42	280.22	0.0	HOMO->L+1 (92%)	0.15
**8**	4.62	268.52	0.0006	H-6->LUMO (90%)	0.36
**9**	4.66	265.89	0.0055	H-2->L+1 (96%)	0.14
**10**	4.75	261.24	0.0004	H-3->L+1 (87%)	0.32
**11**	4.81	257.96	0.5025	H-7->LUMO (13%), H-4->LUMO (11%), H-1->L+1 (62%)	0.72
**12**	4.98	249.18	0.0004	H-2->L+3 (47%), HOMO->L+4 (44%)	0.65
**13**	5.01	247.39	0.1602	H-7->LUMO (68%), H-1->L+2 (16%)	0.73
**14**	5.02	246.82	0.002	H-10->LUMO (11%), H-9->LUMO (70%)	0.39
**15**	5.15	240.91	0.0477	H-8->LUMO (20%), H-4->L+1 (25%), H-1->L+2 (36%)	0.64
**16**	5.25	236.25	0.0	HOMO->L+2 (92%)	0.30
**17**	5.26	235.76	0.0156	H-8->LUMO (67%), H-4->L+1 (21%)	0.42
**18**	5.44	227.92	0.0001	H-1->L+3 (96%)	0.18
**19**	5.46	227.01	0.0823	H-4->L+1 (11%), H-2->L+2 (47%), HOMO->L+3 (26%)	0.51
**20**	5.47	226.70	0.0695	H-4->L+1 (25%), H-2->L+2 (32%), H-1->L+2 (15%)	0.50
**21**	5.49	225.88	0.0034	H-11->LUMO (14%), H-10->LUMO (63%), H-9->LUMO (18%)	0.41
**22**	5.51	225.17	0.0181	H-2->L+2 (18%), H-2->L+4 (20%), HOMO->L+3 (36%)	0.66
**23**	5.56	223.08	0.0004	HOMO->L+5 (93%)	0.23
**24**	5.60	221.21	0.0027	H-1->L+4 (95%)	0.22
**25**	5.63	220.40	0.0015	H-1->L+5 (72%), H-1->L+6 (24%)	0.19

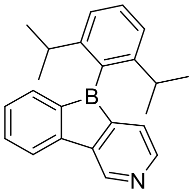

(**DipPBB4**)

<table>
<tr><td>Calculated absorption spectrum</td><td>Orbital</td><td>Energy [eV]</td></tr>
<tr><td rowspan="10">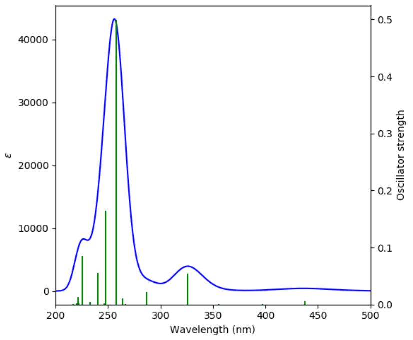

TD-DFT B3LYP/6-31+G(d), gas phase</td><td>L+4</td><td>–0.31</td></tr>
<tr><td>L+3</td><td>–0.47</td></tr>
<tr><td>L+2</td><td>–0.52</td></tr>
<tr><td>L+1</td><td>–1.35</td></tr>
<tr><td>LUMO</td><td>–2.76</td></tr>
<tr><td>HOMO</td><td>–6.40</td></tr>
<tr><td>H-1</td><td>–6.54</td></tr>
<tr><td>H-2</td><td>–6.57</td></tr>
<tr><td>H-3</td><td>–7.16</td></tr>
<tr><td>H-4</td><td>–7.22</td></tr>
<tr><td>Orbitals relevant to the $S_1 \leftarrow S_0$ transition</td><td colspan="2">Other relevant orbitals</td></tr>
<tr><td>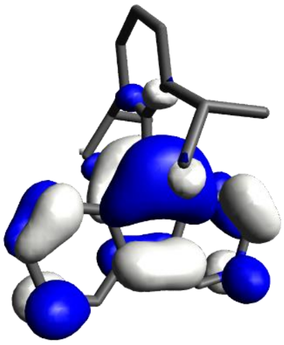
LUMO</td><td>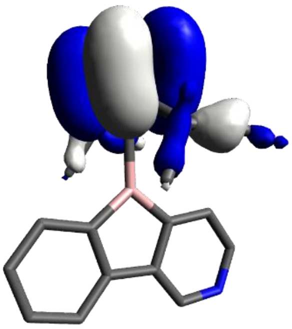
HOMO-1</td><td>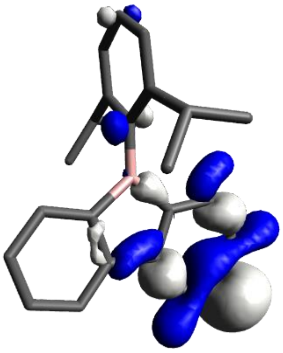
HOMO-3</td></tr>
<tr><td>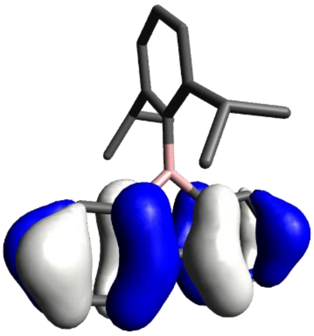
HOMO</td><td>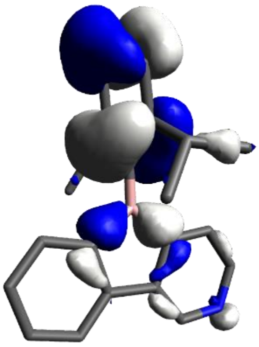
HOMO-2</td><td></td></tr>
</table>

Table S13: Lowest energy singlet electronic transitions of **DipPBB4** (TD-DFT B3LYP/6-31+G(d), gas phase).

State	E [eV]	λ [nm]	*f*	Symmetry	Major contributions	Λ
**1**	2.83	437.89	0.0055	A	HOMO->LUMO (99%)	0.64
**2**	2.97	416.85	0.0001	A	H-2->LUMO (98%)	0.28
**3**	3.12	397.02	0.0007	A	H-1->LUMO (100%)	0.16
**4**	3.49	355.36	0.001	A	H-3->LUMO (98%)	0.38
**5**	3.80	326.00	0.0541	A	H-4->LUMO (84%), HOMO->L+1 (13%)	0.63
**6**	4.33	286.60	0.0216	A	H-5->LUMO (86%)	0.70
**7**	4.56	272.15	0.0001	A	H-3->L+1 (22%), H-2->L+1 (75%)	0.19
**8**	4.65	266.39	0.0007	A	H-7->LUMO (94%)	0.40
**9**	4.70	263.94	0.0102	A	H-1->L+1 (96%)	0.13
**10**	4.80	258.52	0.0002	A	H-3->L+1 (72%), H-2->L+1 (24%)	0.28
**11**	4.81	257.91	0.4997	A	H-6->LUMO (13%), H-4->LUMO (11%), HOMO->L+1 (61%)	0.72
**12**	5.01	247.48	0.1644	A	H-6->LUMO (68%), HOMO->L+1 (10%), HOMO->L+2 (18%)	0.74
**13**	5.03	246.41	0.0024	A	H-2->L+4 (39%), H-1->L+3 (56%)	0.65
**14**	5.11	242.82	0.0002	A	H-10->LUMO (10%), H-9->LUMO (81%)	0.45
**15**	5.16	240.33	0.0552	A	H-4->L+1 (32%), HOMO->L+2 (36%)	0.69
**16**	5.33	232.59	0.0042	A	H-8->LUMO (73%), H-4->L+1 (21%)	0.45
**17**	5.37	231.05	0.0	A	HOMO->L+3 (96%)	0.15
**18**	5.41	229.29	0.0	A	H-2->L+2 (88%)	0.31
**19**	5.49	225.74	0.0849	A	H-8->LUMO (11%), H-5->L+1 (13%), H-4->L+1 (34%), HOMO->L+2 (19%)	0.66
**20**	5.50	225.30	0.001	A	H-1->L+2 (96%)	0.24
**21**	5.58	222.32	0.0024	A	H-2->L+3 (14%), HOMO->L+4 (73%)	0.36
**22**	5.60	221.55	0.0139	A	H-2->L+3 (36%), H-1->L+4 (28%), HOMO->L+4 (24%)	0.65
**23**	5.60	221.52	0.0039	A	H-10->LUMO (85%)	0.35
**24**	5.64	219.82	0.0017	A	HOMO->L+5 (81%), HOMO->L+6 (15%)	0.20
**25**	5.72	216.82	0.0008	A	H-1->L+5 (82%)	0.23

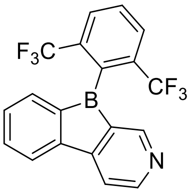

(**^{F}XylPBB1**)

Calculated absorption spectrum	Orbital	Energy [eV]
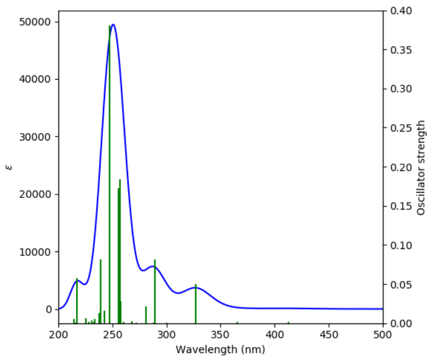 TD-DFT B3LYP/6-31+G(d), gas phase	L+4	–0.38
	L+3	–1.50
	L+2	–1.72
	L+1	–1.98
	LUMO	–2.79
	HOMO	–6.63
	H-1	–7.11
	H-2	–7.26
	H-3	–7.71
	H-4	–7.96
Orbitals relevant to the $S_1 \leftarrow S_0$ transition	Other relevant orbitals	
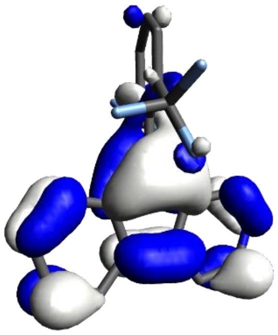 **LUMO**	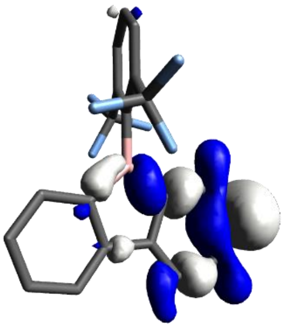 **HOMO-1**	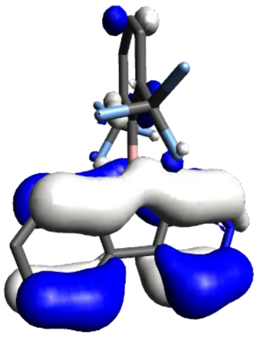 **HOMO-3**
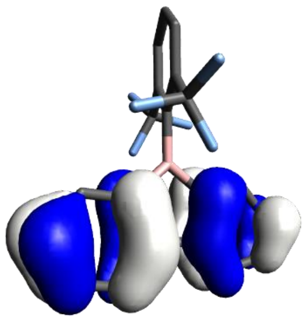 **HOMO**	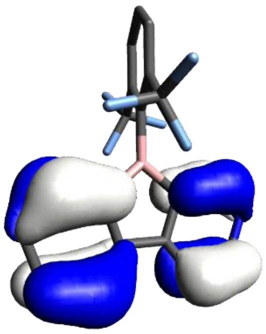 **HOMO-2**	

Table S14 Lowest energy singlet electronic transitions of **^{F}XylPBB1** (TD-DFT B3LYP/6-31+G(d), gas phase).

State	E [eV]	λ [nm]	*f*	Symmetry	Major contributions	Λ
**1**	3.00	413.14	0.0015	A	HOMO->LUMO (99%)	0.64
**2**	3.39	365.43	0.0014	A	H-1->LUMO (98%)	0.35
**3**	3.79	326.97	0.0502	A	H-2->LUMO (82%), HOMO->L+3 (17%)	0.65
**4**	4.13	300.03	0.0007	A	HOMO->L+1 (100%)	0.06
**5**	4.29	289.26	0.0814	A	H-4->LUMO (37%), H-3->LUMO (58%)	0.65
**6**	4.31	287.55	0.0008	A	HOMO->L+2 (98%)	0.24
**7**	4.41	281.07	0.0218	A	H-4->LUMO (58%), H-3->LUMO (33%)	0.59
**8**	4.56	271.81	0.001	A	H-1->L+3 (91%)	0.38
**9**	4.62	268.08	0.0028	A	H-1->L+1 (91%)	0.14
**10**	4.76	260.48	0.0019	A	H-2->L+1 (99%)	0.08
**11**	4.81	257.91	0.0285	A	H-5->LUMO (76%)	0.25
**12**	4.82	256.99	0.1837	A	H-2->L+2 (12%), H-1->L+2 (47%), HOMO->L+3 (23%)	0.38
**13**	4.86	255.28	0.1727	A	H-2->L+2 (26%), H-1->L+2 (45%), HOMO->L+3 (20%)	0.36
**14**	5.02	247.01	0.3809	A	H-2->L+2 (58%), HOMO->L+3 (29%)	0.40
**15**	5.12	242.27	0.0158	A	H-5->LUMO (10%), H-5->L+2 (20%), H-4->L+1 (25%), H-3->L+1 (42%)	0.41
**16**	5.19	238.73	0.0814	A	H-6->LUMO (79%)	0.73
**17**	5.22	237.56	0.0127	A	H-4->L+1 (36%), H-3->L+1 (52%)	0.41
**18**	5.30	233.79	0.0051	A	H-2->L+3 (88%)	0.77
**19**	5.33	232.54	0.0021	A	H-3->L+2 (73%)	0.47
**20**	5.37	230.82	0.004	A	H-8->LUMO (11%), H-7->LUMO (76%)	0.52
**21**	5.44	227.86	0.0017	A	H-8->LUMO (83%)	0.54
**22**	5.50	225.48	0.0067	A	H-3->L+2 (14%), H-3->L+3 (43%), HOMO->L+4 (24%)	0.69
**23**	5.70	217.33	0.0576	A	H-5->L+1 (27%), H-4->L+2 (63%)	0.81
**24**	5.74	215.85	0.0006	A	H-4->L+3 (49%), H-1->L+4 (37%)	0.35
**25**	5.79	214.04	0.0055	A	H-4->L+3 (37%), H-1->L+4 (49%)	0.35

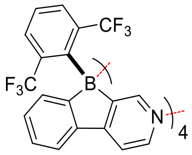

([^{F}XylPBB1]$_4$)

Calculated absorption spectrum	Orbital	Energy [eV]
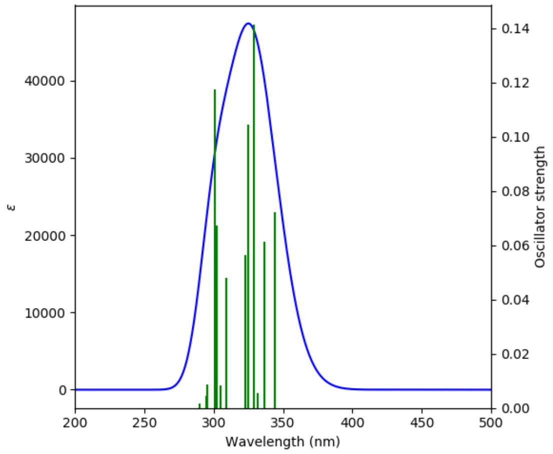 TD-DFT B3LYP/6-31+G(d), gas phase	L+4	–1.62
	L+3	–2.10
	L+2	–2.28
	L+1	–2.37
	LUMO	–2.37
	HOMO	–6.49
	H-1	–6.63
	H-2	–6.63
	H-3	–6.70
	H-4	–7.02

Orbitals relevant to the $S_1 \leftarrow S_0$ transition	Other relevant orbitals
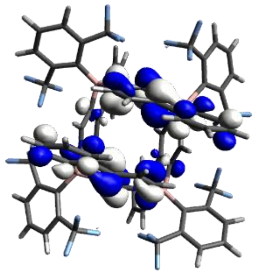 **LUMO**	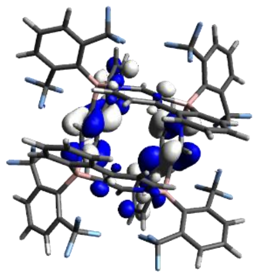 **LUMO+1**
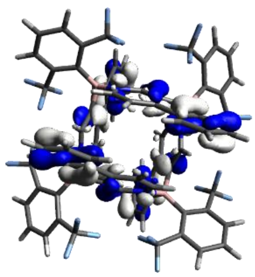 **HOMO**	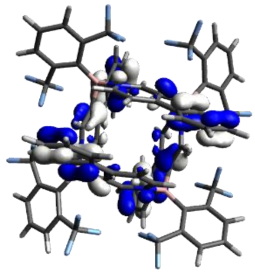 **HOMO-1**

Table S15: Lowest energy singlet electronic transitions of **[^{F}XylPBB1]$_4$** (TD-DFT B3LYP/6-31+G(d), gas phase).

State	E [eV]	λ [nm]	*f*	Major contributions	Λ
**1**	3.60	344.06	0.0721	HOMO->LUMO (96%)	0.69
**2**	3.60	344.06	0.0721	HOMO->L+1 (96%)	0.69
**3**	3.68	336.53	0.0612	H-2->L+1 (10%), H-1->LUMO (10%), HOMO->L+2 (60%)	0.72
**4**	3.72	333.57	0.0	H-2->L+1 (37%), H-1->LUMO (37%)	0.63
**5**	3.73	332.05	0.0056	H-2->L+1 (38%), H-1->LUMO (38%), HOMO->L+2 (23%)	0.65
**6**	3.74	331.15	0.0	H-2->LUMO (38%), H-2->L+1 (10%), H-1->L+1 (38%)	0.67
**7**	3.77	329.12	0.1414	H-2->LUMO (40%), H-1->L+1 (40%), HOMO->L+2 (15%)	0.70
**8**	3.81	325.14	0.1045	H-3->LUMO (57%), H-2->L+2 (14%), H-1->L+2 (22%)	0.66
**9**	3.81	325.13	0.1046	H-3->L+1 (57%), H-2->L+2 (22%), H-1->L+2 (14%)	0.66
**10**	3.84	322.86	0.0563	H-3->L+1 (27%), H-1->L+2 (56%)	0.68
**11**	3.84	322.86	0.0564	H-3->LUMO (27%), H-2->L+2 (56%)	0.68
**12**	3.88	319.48	0.0	HOMO->L+3 (81%)	0.73
**13**	3.91	317.18	0.0	H-3->L+2 (84%)	0.70
**14**	4.01	309.06	0.0479	H-2->L+3 (65%), H-1->L+3 (10%)	0.66
**15**	4.01	309.06	0.0479	H-2->L+3 (10%), H-1->L+3 (65%)	0.66
**16**	4.07	305.00	0.0084	H-6->LUMO (13%), H-5->L+1 (13%), H-4->L+2 (10%), H-3->L+3 (54%)	0.61
**17**	4.10	302.16	0.0672	H-4->LUMO (46%)	0.51
**18**	4.10	302.16	0.0672	H-4->L+1 (46%)	0.51
**19**	4.12	300.79	0.1174	H-6->LUMO (10%), H-5->L+1 (10%), H-4->L+2 (12%), H-3->L+3 (41%)	0.56
**20**	4.15	298.76	0.0	H-7->L+2 (10%), H-6->LUMO (28%), H-5->L+1 (28%)	0.50
**21**	4.20	295.38	0.0085	H-7->LUMO (57%), H-4->LUMO (10%), H-4->L+1 (14%)	0.46
**22**	4.20	295.38	0.0085	H-7->L+1 (57%), H-4->LUMO (14%), H-4->L+1 (10%)	0.46
**23**	4.20	294.91	0.0046	H-6->LUMO (15%), H-6->L+1 (25%), H-5->LUMO (25%), H-5->L+1 (15%), H-4->L+2 (14%)	0.39
**24**	4.21	294.74	0.0	H-6->LUMO (10%), H-6->L+1 (32%), H-5->LUMO (32%), H-5->L+1 (10%)	0.36
**25**	4.28	289.60	0.0018	H-6->L+1 (15%), H-5->LUMO (15%), H-4->L+2 (42%)	0.44

Theoretical calculations: Cartesian coordinates

(BMe_3)TipPBB1(NMe_3)

DFT B3LYP/6-31+G(d), gas phase, S_0

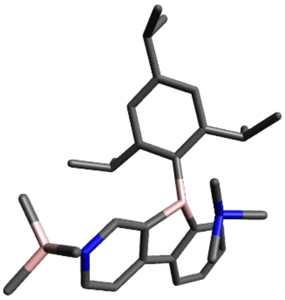

Point group: C_1

Total energy: –883,210.47 kcal mol^{-1}

Dipole moment: 7.97 D

Imaginary frequencies: 0

C	-0.24983000	3.63326100	-0.60960100
C	0.30680100	4.75151100	-1.25241300
C	1.62951100	4.72407100	-1.71177700
C	2.40564300	3.57254300	-1.53760200
C	1.84596700	2.46315500	-0.89348700
C	0.50509600	2.46937000	-0.40367800
C	2.48759200	1.16218500	-0.64032300
C	1.57433700	0.25840400	-0.02708000
B	0.15267600	1.01978600	0.24442700
C	3.79525500	0.75286800	-0.90860800
C	4.18827600	-0.52957900	-0.52227400
N	3.33913400	-1.39419800	0.08293600
C	2.04253100	-1.00768800	0.29521300
C	-1.21195500	0.18661400	-0.15279200
C	-2.51457900	0.36507100	0.42554200
C	-3.49070000	-0.64200900	0.33694800
C	-3.29009300	-1.81185300	-0.40209800
C	-2.12910800	-1.86744600	-1.17653200
C	-1.12627700	-0.88534200	-1.11161300
C	-3.00433800	1.71943400	0.95620600
C	-3.90435700	2.38709200	-0.11825000
C	-3.76456300	1.66229300	2.30167200
C	-0.11100200	-0.88807500	-2.26764200
C	0.51745200	-2.26023800	-2.58708600
C	-0.80475000	-0.31125600	-3.53179800
C	-4.32408200	-2.93229700	-0.44510600
C	-4.5221280	-3.57756900	0.94602400
C	-5.67542300	-2.45811700	-1.02849400
B	3.85882200	-2.88142600	0.69605400
C	5.41115400	-3.10848700	0.25991200
C	2.88388100	-4.03078500	0.06912500
C	3.70000200	-2.73055900	2.32017400
N	0.36010200	1.26970000	2.16755400
C	-0.33407400	2.48261900	2.73413200
C	1.82172000	1.46454300	2.51161200
C	-0.13309600	0.03706500	2.87541100
H	-1.28495800	3.68233300	-0.28826400
H	-0.29665500	5.64178700	-1.40313900
H	2.04939400	5.59109000	-2.21190200
H	3.42626800	3.54460300	-1.90895200
H	4.51662600	1.40488200	-1.38875400
H	5.19462700	-0.88652800	-0.67776500
H	1.41366800	-1.76497900	0.74130200
H	-4.4482410	-0.48658500	0.82521600
H	-2.01540300	-2.68901200	-1.87746700
H	-2.13974900	2.37231700	1.06763000
H	-4.21535200	3.38892900	0.20601000
H	-3.37182200	2.47901100	-1.07055200
H	-4.80620800	1.79042500	-0.29502800
H	-3.98225700	2.67523800	2.66324500
H	-3.20164400	1.13411300	3.08004000
H	-4.72483600	1.14707600	2.19216400
H	0.69984700	-0.20202900	-2.02766800
H	1.28445000	-2.14770700	-3.36292000
H	0.98909000	-2.71046000	-1.70963900
H	-0.22564400	-2.96956100	-2.97010500
H	-0.08794300	-0.24146400	-4.35975300
H	-1.20529000	0.68958300	-3.33714000
H	-1.63457100	-0.95294500	-3.85141100
H	-3.92993300	-3.70872000	-1.11667800
H	-5.22768400	-4.41529800	0.88744400
H	-3.57361400	-3.95501600	1.34366500
H	-4.92236300	-2.84976900	1.66269200
H	-6.37875300	-3.29646300	-1.10270300
H	-5.54418800	-2.02987600	-2.02832000
H	-6.13484900	-1.69224700	-0.39168200
H	6.11457100	-2.37176500	0.68233400
H	5.73105000	-4.08851600	0.64252700
H	5.56875200	-3.13820400	-0.83090200
H	1.81810000	-3.92212300	0.32852400
H	3.19479000	-5.01040500	0.46205800
H	2.95192400	-4.09251900	-1.02781800
H	4.04499400	-3.65511500	2.80651600
H	4.30723400	-1.90783800	2.73197000
H	2.65949000	-2.57392000	2.65234400
H	-1.40207000	2.31726300	2.79001400
H	0.03677500	2.67122100	3.74845400
H	-0.11880800	3.34517200	2.10353400
H	2.39288100	0.57060300	2.28304300
H	1.90721800	1.67673200	3.58341100
H	2.21308000	2.30814700	1.94238600
H	-0.02275300	0.15954700	3.95986700
H	-1.17723900	-0.13532600	2.62037900
H	0.45668300	-0.82078300	2.55365700

(BMe_3)TipPBB2(NMe_3)

DFT B3LYP/6-31+G(d), gas phase, S_0

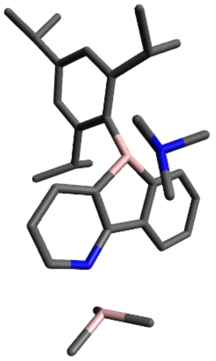

Point group: C_1

Total energy: –883,193.65 kcal mol^{-1}

Dipole moment: 8.27 D

Imaginary frequencies: 0

C	-0.62337100	1.59923100	2.39563400
C	-1.56224400	1.67590300	3.43492100
C	-2.84311300	1.14852500	3.24823400
C	-3.19763200	0.53238400	2.04065400
C	-2.26180100	0.44840100	0.99675700
C	-0.95386500	1.00426900	1.17409900
C	-2.33796500	-0.23514800	-0.33493500
C	-1.06228900	-0.16908400	-0.97916000
B	-0.05172500	0.79096000	-0.14690900
N	-3.39607100	-0.88360200	-0.92407300
C	-3.16046600	-1.54074300	-2.10163600
C	-1.92929800	-1.56546800	-2.73697900
C	-0.86269100	-0.85162000	-2.17444300
C	1.50073600	0.18112400	0.02065600
C	2.79518400	0.76702200	-0.22820600
C	3.96334100	-0.01978700	-0.23333100
C	3.96580500	-1.38400200	0.04660300
C	2.74236600	-1.93709600	0.41683400
C	1.54535100	-1.20430000	0.43688900
C	3.09773900	2.27397800	-0.34967800
C	3.61867200	2.81997300	1.00415600
C	4.07927700	2.65080500	-1.48707300
C	0.36097800	-1.98006300	1.04496100
C	0.07510200	-3.32955600	0.34642300
C	0.59011600	-2.19520300	2.56307800
C	5.23883100	-2.22317200	0.00364400
C	5.84627300	-2.27111400	-1.41734400
C	6.28683500	-1.74097200	1.03324700
N	-0.29535000	2.35582400	-1.05685200
C	0.01716800	3.55402300	-0.17820900
C	-1.74267700	2.57216100	-1.47584200
C	0.49412900	2.33558700	-2.34639900
B	-5.03696200	-0.90866400	-0.38355200
C	-5.08164100	-1.89855400	0.92740600
C	-5.98109800	-1.56595400	-1.54916000
C	-5.49627500	0.66060200	-0.23632300
H	0.37980200	1.98818400	2.55162100
H	-1.29319400	2.13199200	4.38317900
H	-3.57463500	1.20109700	4.04836200
H	-4.18721400	0.12309800	1.93707800
H	-4.00874200	-2.05157900	-2.52276800
H	-1.81787400	-2.12819800	-3.65676000
H	0.11317300	-0.84993000	-2.65110500
H	4.91089000	0.46534200	-0.44195000
H	2.72002300	-2.97981300	0.72000000
H	2.18236400	2.80768800	-0.56384300
H	3.79765500	3.90179600	0.94825800
H	2.90168700	2.62668400	1.81084900
H	4.56041400	2.33244100	1.27988400
H	4.11126300	3.74099700	-1.60679500
H	3.78142700	2.20951200	-2.44554900
H	5.10174700	2.32073500	-1.27785100
H	-0.54133500	-1.38554700	0.96185700
H	-0.83026900	-3.78333300	0.76751900
H	-0.08021600	-3.19734000	-0.72908000
H	0.89421600	-4.04500800	0.48336300
H	-0.27649400	-2.69885300	3.00893700
H	0.72934900	-1.23757300	3.07576800
H	1.47616900	-2.81346400	2.75020000
H	4.95662900	-3.25036700	0.27740500
H	6.72962700	-2.92104100	-1.43973900
H	5.11996900	-2.65321600	-2.14326300
H	6.15603600	-1.27144200	-1.74607100
H	7.16805400	-2.39408200	1.02315300
H	5.87102300	-1.73988100	2.04680100
H	6.62318900	-0.72179000	0.80646600
H	0.86564400	3.36638600	0.46854700
H	0.20793500	4.43130000	-0.80553900
H	-0.84726900	3.73983100	0.45756100
H	-2.03666800	1.82782300	-2.21104500
H	-1.82560100	3.57194300	-1.91604900
H	-2.39132800	2.49989800	-0.60541800
H	0.47254300	3.32722000	-2.81190700
H	1.51660200	2.02363900	-2.17663100
H	0.02144900	1.61506300	-3.01409500
H	-4.22073400	-1.85909000	1.60675300
H	-5.97793300	-1.69038800	1.53292600
H	-5.16257600	-2.94665400	0.60115800
H	-5.96618900	-1.03015100	-2.51217900
H	-7.01548300	-1.49961700	-1.17896100
H	-5.80416100	-2.63567600	-1.74715800
H	-6.49547700	0.69313500	0.22502500
H	-4.85975200	1.33244100	0.35129300
H	-5.60119200	1.11583800	-1.23429700

TipPBB2

DFT B3LYP/6-31G, gas phase, S_0

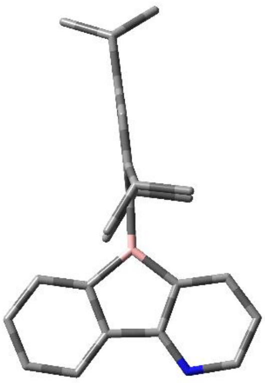

Point group: C_1
Total energy: –682,866.42 kcal mol^{-1}
Dipole moment: 1.49 D
Imaginary frequencies: 0

C	-1.75597300	-2.55849500	-0.11321600
C	-1.86223200	-1.16727000	-0.07157800
C	-3.17418100	-0.60956300	-0.11577500
H	-0.78494000	-3.04421200	-0.08296700
C	-1.76678300	1.30221400	0.01612400
C	-1.49812500	2.67201100	0.07522100
C	-2.56038600	3.59478800	0.05525300
C	-3.88495700	3.14749400	-0.02355600
C	-4.17786800	1.77248400	-0.08395600
C	-3.12045800	0.87068600	-0.06346600
H	-0.47280400	3.02621700	0.13640600
H	-2.35397500	4.65967200	0.10106700
H	-4.69584000	3.86944200	-0.03823900
H	-5.20079000	1.41670600	-0.14510700
B	-0.82836100	0.02651400	0.01833500
C	0.73953000	-0.00974700	0.09353800
C	1.53892200	0.04131400	-1.09283800
C	1.41834700	-0.04254300	1.35089300
C	2.93774700	0.11395000	-0.99185400
C	2.82168300	0.03225600	1.38876100
C	3.60536800	0.12294300	0.23687500
H	3.51558500	0.16280800	-1.91147600
H	3.31568500	0.01755000	2.35790300
C	0.74415400	-0.24785200	2.71937800
H	1.49543300	0.05880000	3.46087600
C	0.99821200	-0.06998300	-2.53004200
H	1.81568600	0.28273300	-3.17474000
C	0.46061000	-1.74936100	2.97884000
H	-0.30513800	-2.12923400	2.29434500
H	1.36766500	-2.34845500	2.84299000
H	0.09966100	-1.89884900	4.00414100
C	-0.51046400	0.59722500	3.01095000
H	-0.34869600	1.65660100	2.78946000
H	-1.38394100	0.26320000	2.44157400
H	-0.77255300	0.50549900	4.07219000
C	0.74475700	-1.54980000	-2.91475200
H	1.63665500	-2.15966200	-2.73392600
H	-0.08116800	-1.97195200	-2.33270600
H	0.48279200	-1.62935500	-3.97726200
C	-0.22517800	0.79725800	-2.88288100
H	-1.14832200	0.42877800	-2.42382900
H	-0.08856700	1.83873000	-2.57600700
H	-0.38396800	0.77813900	-3.96812600
C	-2.93316800	-3.32746700	-0.19550400
H	-2.89076800	-4.41052500	-0.22981000
C	-4.16842700	-2.67754100	-0.23293300
H	-5.09208900	-3.24376700	-0.29623600
N	-4.30725700	-1.32338500	-0.19412600
C	5.12572700	0.22017400	0.32615500
H	5.38764200	0.20324600	1.39418200
C	5.65222800	1.55044100	-0.26018000
C	5.82118000	-0.98877300	-0.34135200
H	5.43059500	1.62351100	-1.33181700
H	5.19091900	2.41051800	0.23752700
H	6.73959200	1.62346800	-0.13644100
H	5.47877400	-1.93236000	0.09764600
H	5.60889500	-1.02157500	-1.41685500
H	6.90897900	-0.92561200	-0.21618600

TipPBB1

DFT B3LYP/6-31G, gas phase, S_0

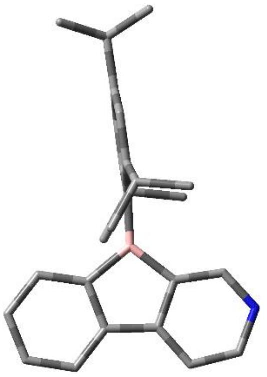

Point group: C1
Total energy: –683,136.40 kcal mol^{-1}
Dipole moment: 2.83 D
Imaginary frequencies: 0

C	4.03262989	-2.79875575	-0.22492747
C	1.76049707	-2.52932920	-0.10530449
C	1.86880150	-1.14286707	-0.06837388
C	3.16994412	-0.57700028	-0.11491162
C	4.27386132	-1.41480300	-0.19455101
H	4.87059692	-3.49109534	-0.28707986
H	0.78526341	-3.01159892	-0.07225277
H	5.29235792	-1.03776586	-0.23293453
C	1.73841260	1.31382059	0.01298641
C	1.43856995	2.67142854	0.06924961
C	2.47526357	3.61713083	0.04750540
C	3.80643445	3.19871652	-0.03037620
C	4.12724495	1.83288439	-0.08792479
C	3.09327789	0.90381555	-0.06584963
H	0.40238949	2.99716807	0.12988156
H	2.24593183	4.67854550	0.09121342
H	4.60330299	3.93761977	-0.04667246
H	5.16666264	1.51950656	-0.14827086
B	0.82533332	0.02738312	0.01881239
C	-0.73392966	-0.01519463	0.09500247
C	-1.40272705	-0.04242653	1.34672512
C	-1.52482675	0.03435390	-1.08588485
C	-2.80142354	0.03851264	1.38914464
C	-2.91839045	0.11360673	-0.98767947
C	-3.58290069	0.13006564	0.23960470
H	-3.29481744	0.02565880	2.35988294
H	-3.49699716	0.15897954	-1.90800964
C	-0.96795956	-0.09851912	-2.50395419
H	-1.76212830	0.26343748	-3.17003186
C	-0.71149152	-0.25965129	2.69320081
H	-1.43566253	0.05959882	3.45412145
C	-0.73457206	-1.58217953	-2.85254668
H	0.06765493	-2.00646791	-2.24064349
H	-1.64055446	-2.17348645	-2.68055086
H	-0.44640555	-1.68980845	-3.90583575
C	0.28034485	0.73040585	-2.83077829
H	0.17213872	1.77537672	-2.52500860
H	1.18297542	0.33174464	-2.35588378
H	0.46243351	0.70458456	-3.91210307
C	-0.45050083	-1.76118463	2.92695581
H	-1.37096893	-2.34330868	2.80902878
H	0.28653265	-2.14546019	2.21479450
H	-0.06113425	-1.93173301	3.93856727
C	0.56601528	0.55058606	2.94560746
H	1.41579142	0.18391778	2.36014740
H	0.43201972	1.61211067	2.71676233
H	0.85399954	0.45872490	3.99999415
N	2.81889374	-3.36061937	-0.18255810
C	-5.09556048	0.23349282	0.32577514
H	-5.36057622	0.21950915	1.39195041
C	-5.78667191	-0.96865161	-0.34165658
H	-5.43969081	-1.91347990	0.09136098
H	-6.87456456	-0.90938525	-0.21424256
H	-5.57696733	-0.99667390	-1.41771434
C	-5.60741873	1.55923609	-0.26462127
H	-5.13471474	2.41781438	0.22537933
H	-5.38753849	1.62313846	-1.33714642
H	-6.69379840	1.64528699	-0.13936190

DipPBB1

DFT B3LYP/6-31G, gas phase, S_0

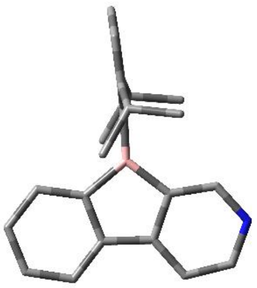

Point group: C_1
Total energy: –608,870.85 kcal mol^{-1}
Dipole moment: 3.14 D
Imaginary frequencies: 0

C	3.01845500	3.07678400	0.00007300
C	0.75124200	2.58608000	0.00000400
C	0.99496200	1.21306500	0.00000800
C	2.35449800	0.77652800	0.00003200
C	3.38000700	1.71715200	0.00006800
H	3.78418500	3.84642300	0.00009100
H	-0.26002200	2.98068500	-0.00002000
H	4.42750100	1.43380200	0.00007900
C	1.10274200	-1.25147500	-0.00013900
C	0.93998200	-2.63832200	-0.00026500
C	2.06875500	-3.47820800	-0.00028500
C	3.35538600	-2.92866300	-0.00018300
C	3.54118200	-1.53384200	-0.00006600
C	2.42026000	-0.70792900	-0.00004300
H	-0.05709600	-3.06965400	-0.00033700
H	1.94351900	-4.55644300	-0.00038000
H	4.22106200	-3.58379400	-0.00020600
H	4.54570700	-1.12095300	-0.00000400
B	0.06050700	-0.05918600	-0.00007100
C	-1.50614100	-0.16649300	0.00001500
C	-2.23695300	-0.24672600	-1.22812600
C	-2.23682800	-0.24681500	1.22825400
C	-3.62677500	-0.45866900	-1.19919600
C	-3.62664300	-0.45875600	1.19944500
C	-4.32325900	-0.57731700	0.00015200
H	-4.16458300	-0.52791700	-2.14084500
H	-4.16436900	-0.52804900	2.14113800
C	-1.64379100	-0.02387000	2.63102700
H	-2.38779100	-0.43532100	3.32783200
C	-1.64407100	-0.02367400	-2.63094800
H	-2.38807400	-0.43518900	-3.32771500
C	-1.53217600	1.48864700	2.95066800
H	-0.78664500	1.97304800	2.31154500
H	-2.49173300	1.99380500	2.79639800
H	-1.22883000	1.63755500	3.99447500
C	-0.31951000	-0.74275800	2.95116600
H	-0.35801500	-1.80496800	2.69075100
H	0.53559800	-0.29699700	2.43232300
H	-0.11225600	-0.66106600	4.02522900
C	-1.53268200	1.48890300	-2.95043200
H	-2.49229600	1.99392100	-2.79606200
H	-0.78719100	1.97331000	-2.31125400
H	-1.22938300	1.63798400	-3.99422700
C	-0.31970800	-0.74230700	-2.95125900
H	0.53535700	-0.29648600	-2.43239300
H	-0.35802500	-1.80456700	-2.69103400
H	-0.11251700	-0.66039100	-4.02531700
N	1.74349900	3.51880500	0.00003600
H	-5.39473800	-0.75244500	0.00021200

DipPBB2

DFT B3LYP/6-31G, gas phase, S_0

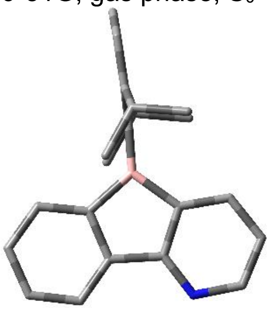

Point group: C_1
Total energy: –608,873.07 kcal mol^{-1}
Dipole moment: 1.16 D
Imaginary frequencies: 0

C	0.75090300	2.60022100	0.00011500
C	0.97767200	1.22282900	0.00003400
C	2.33445600	0.78283300	-0.00003400
H	-0.25950700	2.99871500	0.00017200
C	1.09690400	-1.24738900	-0.00007700
C	0.94777800	-2.63655800	-0.00014300
C	2.08727200	-3.46234400	-0.00022200
C	3.36934500	-2.89928100	-0.00023400
C	3.54211000	-1.50274400	-0.00017300
C	2.40938600	-0.69741100	-0.00010000
H	-0.04387200	-3.08055100	-0.00014000
H	1.97384600	-4.54205000	-0.00027200
H	4.24064300	-3.54716800	-0.00029100
H	4.53131300	-1.05761000	-0.00018400
B	0.05121200	-0.05865200	0.00001500
C	-1.51624300	-0.16281500	0.00003500
C	-2.24772500	-0.23830300	-1.22807200
C	-2.24767600	-0.23845100	1.22815600
C	-3.63921400	-0.43947300	-1.19920600
C	-3.63917000	-0.43962100	1.19931600
C	-4.33682100	-0.55234900	0.00006200
H	-4.17735800	-0.50609400	-2.14088600
H	-4.17727700	-0.50635900	2.14100800
C	-1.65246300	-0.02483000	2.63163900
H	-2.40330300	-0.42567300	3.32725700
C	-1.65256300	-0.02452300	-2.63154900
H	-2.40343700	-0.42526200	-3.32719200
C	-1.51941300	1.48536200	2.95404500
H	-0.76369400	1.95843200	2.31821500
H	-2.47108600	2.00493200	2.79769000
H	-1.21703500	1.62789900	3.99904800
C	-0.33928100	-0.76365500	2.95260900
H	-0.39269200	-1.82397800	2.68750100
H	0.52391000	-0.32999000	2.43738000
H	-0.13396200	-0.68952700	4.02759800
C	-1.51947400	1.48569800	-2.95377300
H	-2.47112100	2.00528100	-2.79730600
H	-0.76370500	1.95867900	-2.31793400
H	-1.21715000	1.62834300	-3.99877700
C	-0.33941300	-0.76334700	-2.95265400
H	0.52379700	-0.32979900	-2.43735900
H	-0.39286400	-1.82371500	-2.68772700
H	-0.13409800	-0.68903900	-4.02763100
H	-5.40956500	-0.71968600	0.00007000
C	1.85814800	3.47077300	0.00011900
H	1.72188900	4.54659300	0.00018000
C	3.14639500	2.93214200	0.00004600
H	4.01839200	3.57817900	0.00004400
N	3.40234400	1.59459200	-0.00002600

TipPBB3

DFT B3LYP/6-31G, gas phase, S_0

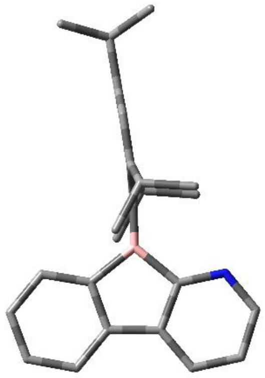

Point group: C_1

Total energy: –682, 862.83 kcal mol^{-1}

Dipole moment: 2.87 D

Imaginary frequencies: 0

C	4.15292100	-2.71866500	-0.23830500
C	1.92793900	-1.11460500	-0.07478100
C	3.22082000	-0.51667300	-0.11525400
C	4.35021700	-1.32846300	-0.19818200
H	4.99951300	-3.39400800	-0.30286500
H	5.35278700	-0.91280700	-0.23129700
C	1.72530300	1.33826900	0.02229700
C	1.39320900	2.69271500	0.08625600
C	2.40760300	3.66784600	0.07243200
C	3.75000100	3.28319700	-0.00523100
C	4.10672300	1.92302400	-0.07055300
C	3.09932900	0.96227300	-0.05669400
H	0.35107800	2.99353600	0.14667000
H	2.15018300	4.72117400	0.12208000
H	4.52782000	4.04068400	-0.01542600
H	5.15408100	1.64130000	-0.13051800
B	0.83279500	0.03296600	0.01847800
C	-0.72983400	-0.04129700	0.09166500
C	-1.40797300	-0.08180000	1.34711700
C	-1.52815500	0.00817800	-1.09333800
C	-2.80844900	0.02582200	1.38841300
C	-2.92435600	0.11357100	-0.99359800
C	-3.58950400	0.14639600	0.23676900
H	-3.30180200	0.00613700	2.35787000
H	-3.50139100	0.16138900	-1.91386800
C	-0.97920400	-0.18149200	-2.51604900
H	-1.77442400	0.16455100	-3.19187000
C	-0.72745900	-0.37044900	2.69403100
H	-1.45382600	-0.07598300	3.46507800
C	-0.76798200	-1.69119200	-2.80377500
H	-0.03523100	-2.12552300	-2.11491300
H	-1.70691600	-2.24209800	-2.68022900
H	-0.41415500	-1.84113900	-3.83192600
C	0.27885300	0.62563000	-2.88758200
H	0.17324000	1.68650800	-2.64001200
H	1.17965000	0.24830900	-2.39086500
H	0.46075800	0.54169000	-3.96611500
C	-0.49515100	-1.89611200	2.85209400
H	-1.44336100	-2.44011100	2.77908900
H	0.16758400	-2.27742400	2.06756500
H	-0.04607200	-2.11781200	3.82879600
C	0.56309600	0.41230800	3.00068800
H	1.41107800	0.07461000	2.39448300
H	0.43786600	1.48778800	2.84078400
H	0.84681300	0.25212800	4.04816500
C	2.85418400	-3.23581500	-0.19465200
H	2.68080300	-4.30606600	-0.22450400
N	1.74696400	-2.45299000	-0.11383000
C	-5.10660000	0.28461000	0.32767300
H	-5.36753400	0.27757500	1.39610000
C	-5.83565800	-0.90675400	-0.33558300
C	-5.59769000	1.62701100	-0.26169900
H	-5.62602700	-0.94797100	-1.41130400
H	-5.51712900	-1.85783900	0.10497800
H	-6.92128200	-0.81431900	-0.20864900
H	-5.11316300	2.47561300	0.23381800
H	-5.37398500	1.69125300	-1.33350600
H	-6.68277500	1.72975700	-0.13815200

DipPBB3

DFT B3LYP/6-31G, gas phase, S_0

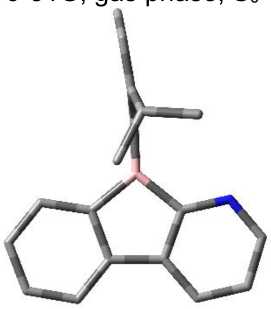

Point group: C_1
Total energy: –608,869.52 kcal mol^{-1}
Dipole moment: 3.13 D
Imaginary frequencies: 0

C	3.04602400	3.04985800	0.00003600
C	1.01832600	1.19577300	0.00002800
C	2.37256400	0.75219100	-0.00005600
C	3.40212100	1.69102500	-0.00005000
H	3.81013500	3.81997100	0.00004300
H	4.44678200	1.39494600	-0.00011000
C	1.09858300	-1.26616300	-0.00010100
C	0.92384600	-2.65122100	-0.00016000
C	2.04456200	-3.50217400	-0.00024600
C	3.33511800	-2.96327300	-0.00027400
C	3.53363100	-1.56961800	-0.00021600
C	2.42167000	-0.73208500	-0.00013100
H	-0.07771300	-3.07198000	-0.00014000
H	1.90957000	-4.57918900	-0.00029200
H	4.19551900	-3.62552100	-0.00034100
H	4.54269200	-1.16750800	-0.00023900
B	0.06200100	-0.07262900	-0.00000700
C	-1.50109000	-0.18316300	0.00004400
C	-2.22982200	-0.27541000	-1.22646700
C	-2.22973600	-0.27555500	1.22659600
C	-3.60806600	-0.55028600	-1.19988500
C	-3.60798300	-0.55042500	1.20007600
C	-4.29647300	-0.71009400	0.00011000
H	-4.14358100	-0.62992300	-2.14213200
H	-4.14343300	-0.63017300	2.14235100
C	-1.64319500	0.03728100	2.61185600
H	-2.36081600	-0.37037500	3.33827500
C	-1.64337200	0.03758800	-2.61172900
H	-2.36101900	-0.37002000	-3.33814800
C	-1.60036900	1.57216100	2.83291100
H	-0.95565100	2.05958400	2.09340300
H	-2.60258500	2.00390700	2.73545900
H	-1.22161300	1.80577700	3.83624400
C	-0.28297300	-0.60099000	2.95059000
H	-0.27291700	-1.67644300	2.74771200
H	0.54379400	-0.14235300	2.39692100
H	-0.06488500	-0.45083000	4.01510200
C	-1.60061300	1.57249200	-2.83263100
H	-2.60284000	2.00419100	-2.73508400
H	-0.95587600	2.05986500	-2.09310800
H	-1.22191700	1.80622300	-3.83596000
C	-0.28314300	-0.60059800	-2.95060000
H	0.54363500	-0.14199400	-2.39692000
H	-0.27303900	-1.67607300	-2.74784000
H	-0.06511400	-0.45031400	-4.01510700
H	-5.35832400	-0.93691100	0.00013400
C	1.69520800	3.41234800	0.00011200
H	1.39969700	4.45590800	0.00017900
N	0.68437100	2.50491500	0.00010800

TipPBB4

DFT B3LYP/6-31G, gas phase, S_0

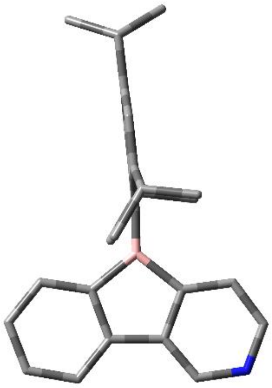

Point group: C_1
Total energy: –682,862.76 kcal mol^{-1}
Dipole moment: 2.72 D
Imaginary frequencies: 0

C	-1.75312800	-2.54565800	-0.11416900
C	-1.87740100	-1.15705600	-0.07279300
C	-3.18904000	-0.60019400	-0.11694600
C	-4.29210300	-1.44428500	-0.19890100
H	-0.78029100	-3.02637200	-0.08375100
H	-5.30991900	-1.06848800	-0.23509300
C	-1.76751700	1.31018300	0.01714600
C	-1.48448200	2.67690700	0.07785100
C	-2.53333600	3.61360500	0.05946700
C	-3.86081700	3.17879600	-0.01956900
C	-4.16816900	1.80679000	-0.08157300
C	-3.12603400	0.88335400	-0.06305600
H	-0.45434300	3.01681500	0.13921800
H	-2.31539000	4.67587500	0.10652600
H	-4.66584600	3.90719300	-0.03335700
H	-5.20394800	1.48669900	-0.14250200
B	-0.83309700	0.03830900	0.01856400
C	0.73337600	-0.00469100	0.09351300
C	1.53269700	0.04622600	-1.09294600
C	1.41200100	-0.03949600	1.35090600
C	2.93129800	0.12068800	-0.99174900
C	2.81513900	0.03718900	1.38892300
C	3.59869700	0.13044900	0.23713700
H	3.50912900	0.16984400	-1.91133300
H	3.30899800	0.02140000	2.35809800
C	0.73882400	-0.25311600	2.71844300
H	1.48060700	0.07322300	3.46110900
C	0.99297300	-0.07124600	-2.52987800
H	1.80187600	0.30029700	-3.17481200
C	0.48857700	-1.76030400	2.98056200
H	-0.26484600	-2.15941700	2.29316600
H	1.40955000	-2.33885400	2.85017000
H	0.12641600	-1.91571900	4.00449200
C	-0.53629200	0.56218600	3.00362300
H	-0.39952800	1.62523100	2.78332900
H	-1.39927300	0.20588700	2.43103300
H	-0.80151900	0.46360500	4.06341700
C	0.77016400	-1.55585000	-2.91598400
H	1.67515700	-2.14683600	-2.73811500
H	-0.04482300	-1.99643800	-2.33198400
H	0.50715200	-1.63983500	-3.97783600
C	-0.24965900	0.76871800	-2.87974200
H	-1.16347500	0.37896500	-2.41876000
H	-0.13593600	1.81341700	-2.57481100
H	-0.41133800	0.74437800	-3.96440400
C	-2.92184300	-3.32695900	-0.19712000
H	-2.86662900	-4.41015100	-0.23138800
N	-4.16062000	-2.79716900	-0.23873500
C	5.11884500	0.23019300	0.32658100

H	5.38070100	0.21301600	1.39458700
C	5.64296200	1.56176400	-0.25893700
C	5.81625400	-0.97718700	-0.34169700
H	5.42153000	1.63502500	-1.33060200
H	5.18015500	2.42080400	0.23919200
H	6.73013000	1.63654300	-0.13489000
H	5.47533600	-1.92165200	0.09650700
H	5.60440900	-1.00954900	-1.41729200
H	6.90386700	-0.91230400	-0.21615200

DipPBB4

DFT B3LYP/6-31G, gas phase, S_0

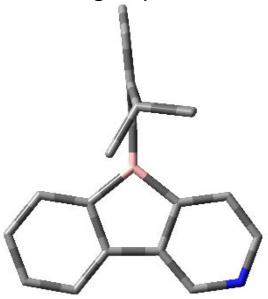

Point group: C_1
Total energy: –608,869.38 kcal mol^{-1}
Dipole moment: 2.48 D
Imaginary frequencies: 0

C	0.73583200	2.58957600	0.00007500
C	0.98801600	1.21777200	0.00000500
C	2.34681700	0.78615600	-0.00006800
C	3.36830600	1.73089200	-0.00006900
H	-0.27814400	2.97726700	0.00013400
H	4.41747900	1.45222700	-0.00013100
C	1.10620600	-1.25089100	-0.00009900
C	0.95006600	-2.63919000	-0.00015800
C	2.08198600	-3.47383700	-0.00024800
C	3.36500500	-2.91585500	-0.00027700
C	3.54481400	-1.51998000	-0.00022100
C	2.42094500	-0.69786600	-0.00013300
H	-0.04526700	-3.07485700	-0.00014300
H	1.96273600	-4.55266000	-0.00029400
H	4.23460800	-3.56591500	-0.00034200
H	4.54768900	-1.10377500	-0.00024500
B	0.05834700	-0.07136200	-0.00000400
C	-1.50778000	-0.17704600	0.00005700
C	-2.23882200	-0.25515700	-1.22813600
C	-2.23871200	-0.25530000	1.22830300
C	-3.62891900	-0.46518100	-1.19921200
C	-3.62881300	-0.46532000	1.19947900
C	-4.32550800	-0.58319100	0.00015800
H	-4.16674600	-0.53409900	-2.14087700
H	-4.16655400	-0.53435400	2.14118400
C	-1.64607500	-0.03142500	2.63111400
H	-2.38419400	-0.45372700	3.32764500
C	-1.64630700	-0.03111400	-2.63097000
H	-2.38447400	-0.45336000	-3.32748500
C	-1.55196000	1.48158200	2.95505300
H	-0.81183700	1.97541300	2.31648800
H	-2.51753000	1.97596600	2.80292300
H	-1.24934900	1.63105100	3.99895800
C	-0.31243800	-0.73393500	2.94732700
H	-0.33718800	-1.79583600	2.68464400
H	0.53659200	-0.27615900	2.42866000
H	-0.10443800	-0.65174500	4.02116400
C	-1.55225200	1.48192600	-2.95475400
H	-2.51781900	1.97627200	-2.80247900
H	-0.81207900	1.97571000	-2.31621200
H	-1.24974600	1.63151000	-3.99867300
C	-0.31268000	-0.73355900	-2.94737400
H	0.53638300	-0.27582400	-2.42872800
H	-0.33738300	-1.79549300	-2.68481100
H	-0.10477400	-0.65123800	-4.02121900
H	-5.39707800	-0.75766700	0.00019400
C	1.82863200	3.47784400	0.00007100
H	1.67361500	4.55183300	0.00012700
N	3.11222900	3.06640900	0.00000000

^{F}XylPBB1

DFT B3LYP/6-31G, gas phase, S_0

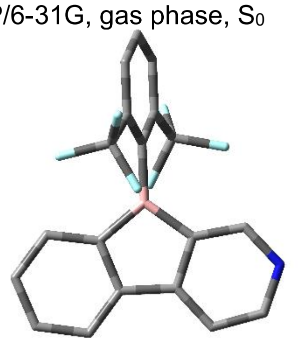

Point group: C_1

Total energy: –883,786.76 kcal mol^{-1}

Dipole moment: 3.51 D

Imaginary frequencies: 0

C	3.29642000	-2.18270700	-2.11069600
C	1.01380100	-1.89824600	-1.81032400
C	1.21576400	-0.90354000	-0.85654600
C	2.55783300	-0.54875200	-0.52651900
C	3.61349000	-1.19540700	-1.15863200
H	4.08734300	-2.71229400	-2.63275100
H	0.01827900	-2.22149300	-2.09500200
H	4.65080700	-0.96229600	-0.94232300
C	1.23248200	0.89262000	0.83907900
C	1.01938400	1.89105400	1.79163400
C	2.11828800	2.51740300	2.40764600
C	3.42204200	2.14405600	2.06826100
C	3.65835700	1.14124800	1.10855200
C	2.56975100	0.52423700	0.50226300
H	0.01128900	2.19665000	2.05268100
H	1.95468400	3.29442500	3.14727100
H	4.26410300	2.63264900	2.54858200
H	4.67675000	0.86339800	0.85442600
B	0.26274100	-0.00139600	-0.00470700
C	-1.31819900	0.00262100	0.00344200
C	-2.06758200	-1.03430600	0.60788900
C	-2.07115500	1.04098900	-0.59361300
C	-3.46796400	-1.03645700	0.61752100
C	-3.47183300	1.04488900	-0.59152300
C	-4.17342700	0.00466200	0.01578000
H	-3.99699200	-1.84860000	1.10142000
H	-4.00394200	1.85696300	-1.07220500
N	2.03767700	-2.53758500	-2.44014500
C	-1.37531200	2.21399700	-1.20370300
F	-2.13327700	2.82780100	-2.19447800
F	-0.15578800	1.85911700	-1.78748000
F	-1.08314000	3.20921800	-0.26652100
C	-1.36688700	-2.20818500	1.21150100
F	-2.12021300	-2.82526300	2.20407000
F	-0.14663000	-1.84979000	1.79370400
F	-1.07365000	-3.19832900	0.27190600
H	-5.25699700	0.00484600	0.01956500

$[^{F}$XylPBB1$]_4$

DFT B3LYP/6-31G, gas phase, S_0

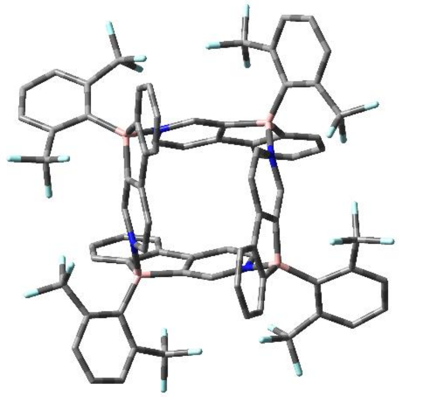

Point group: C_2

Total energy: –3, 535,213.90 kcal mol^{-1}

Dipole moment: 0.00 D

Imaginary frequencies: 0

C	2.82239900	-0.72083500	0.08056100
H	3.15814500	-0.95082400	-0.91889400
C	2.60437700	0.97827900	1.69289500
H	2.75833800	2.01950500	1.93826200
C	-2.82239400	0.72088600	0.08060300
H	-3.15816300	0.95092700	-0.91883300
C	2.28067400	-1.67336000	0.93267200
N	2.96078200	0.59584000	0.43481600
C	-2.28069400	1.67338600	0.93275600
N	-0.59578600	2.96075600	-0.43478400
C	0.72090600	2.82245200	-0.08057200
H	0.95091500	3.15817000	0.91888800
N	-2.96071800	-0.59581000	0.43479000
C	1.67345300	2.28077400	-0.93270300
N	0.59576000	-2.96073400	-0.43491500
C	2.32836400	1.24206400	-2.97708500
C	1.86864400	-1.24115300	2.22058400
C	-1.86865200	1.24113500	2.22064500
C	-0.09210400	2.06291900	-2.60789600
H	-0.45594900	1.77723300	-3.58739800
C	-1.14354700	3.50685800	2.18413300
C	-2.60428000	-0.97829900	1.69284600
H	-2.75817000	-2.01954600	1.93816200
C	1.24195700	-2.32830300	2.97703700
C	4.61378800	0.16005100	-4.12202400
H	5.51097800	-0.25331900	-4.57321100
C	-0.72093900	-2.82244300	-0.08071100
H	-0.95094200	-3.15816500	0.91874800
C	1.14345300	-3.50684800	2.18396600
C	-1.67346500	-2.28074800	-0.93283500
C	-1.24123700	-1.86867300	-2.22072400
C	1.24121800	1.86869000	-2.22058100
C	-0.97824700	2.60429100	-1.69284800
H	-2.01949500	2.75815300	-1.93817500
C	3.50695400	1.14363300	-2.18406900
C	-3.50692200	-1.14345200	-2.18410800
C	4.64990200	0.60293300	-2.78946900
H	5.57549600	0.51947800	-2.23514100
C	2.06297100	0.09213900	2.60794100
H	1.77734300	0.45597500	3.58746200
C	-0.25445800	3.43822400	4.87869100
H	0.08372700	3.42585600	5.91006900
C	0.97821300	-2.60430600	-1.69298400
H	2.01945900	-2.75817400	-1.93832000
C	-2.06291100	-0.09218400	2.60793200
H	-1.77726900	-0.45605600	3.58743600
C	0.60262700	-4.64978500	2.78926700
H	0.51912300	-5.57533400	2.23486800
C	-1.24202600	2.32827600	2.97715500
C	2.84909300	-4.38497200	0.15162000
C	0.09207000	-2.06294500	-2.60805000

H	0.45592300	-1.77728000	-3.58755400
C	-2.32837300	-1.24196200	-2.97717300
C	0.15970300	-4.61370700	4.12181000
H	-0.25375600	-5.51088700	4.57293600
C	4.38526400	2.84902300	-0.15166000
C	0.80276300	-2.28586300	4.30626100
H	0.90496600	-1.38016800	4.89796300
C	-0.60281200	4.64979500	2.78952400
H	-0.51933700	5.57538000	2.23518800
C	2.28588500	0.80291900	-4.30632200
H	1.38015400	0.90508700	-4.89797500
C	-2.84915500	4.38495400	0.15164000
C	-0.80289600	2.28579700	4.30639700
H	-0.90509700	1.38007400	4.89805700
C	2.48741800	-5.49003300	-0.68441200
C	5.49046400	2.48705000	0.68408600
C	3.43832300	0.25451100	-4.87862000
H	3.42595500	-0.08369200	-5.90999200
C	4.24147400	-4.35860700	0.47903300
C	6.17249800	4.79242600	1.04342900
C	-5.49015700	-2.48725300	0.68432500
C	-0.15994600	4.61367800	4.12208500
H	0.25344700	5.51085800	4.57327000
C	-4.24158100	4.35851500	0.47889100
C	0.25424300	-3.43829100	4.87847400
H	-0.08398700	-3.42595700	5.90983800
C	4.35906100	4.24148300	-0.47885200
C	-4.38509500	-2.84903700	-0.15167700
C	-2.28591500	-0.80275100	-4.30639000
H	-1.38021900	-0.90496400	-4.89808800
C	-4.64984900	-0.60261200	-2.78942000
H	-5.57540500	-0.51910800	-2.23503800
C	-2.48746000	5.49002100	-0.68438500
C	6.33507600	3.43633900	1.28679800
H	7.13901200	3.09723700	1.92735600
C	3.43690900	-6.33428600	-1.28726500
H	3.09802600	-7.13815900	-1.92802200
C	-4.35875200	-4.24146300	-0.47892200
C	5.20925900	5.18365600	0.12273900
H	5.12059000	6.22618800	-0.15263800
B	3.23812000	1.77136400	-0.69508900
C	-5.18398900	5.20826500	-0.12297200
H	-6.22648600	5.11945800	0.15250400
B	1.77127500	-3.23801300	0.69506100
B	-3.23809900	-1.77129300	-0.69519900
C	-5.20858900	-5.18380600	0.12293000
H	-5.11984500	-6.22631200	-0.15253100
B	-1.77131100	3.23804200	0.69517900
C	-3.43833000	-0.25421200	-4.87860800
H	-3.42598000	0.08402900	-5.90996900
C	-6.33441800	-3.43667500	1.28727900
H	-7.13827600	-3.09771800	1.92801700
C	-4.61375300	-0.15967200	-4.12195700
H	-5.51092100	0.25380500	-4.57308900
C	-6.17160900	-4.79275000	1.04391400
C	5.18390100	-5.20841900	-0.12273500
H	6.22637800	-5.11965600	0.15282300
C	-3.43695000	6.33420500	-1.28735200
H	-3.09805700	7.13808000	-1.92810000
C	4.79295600	-6.17144700	-1.04376200
C	-4.79300900	6.17129700	-1.04397900
H	5.52431900	-6.81271700	-1.52129700
H	-6.81289100	-5.52405300	1.52152300
H	6.81408600	5.52361200	1.52080600
H	-5.52436400	6.81251300	-1.52159900
C	4.84687700	-3.43191500	1.49776800
F	6.03604400	-3.95514700	2.02112400
F	5.20126400	-2.18772600	0.97514900
F	4.03773400	-3.20022600	2.60114600
C	5.89895300	1.07161300	0.98995800
F	7.27705500	0.97835800	1.21504400
F	5.31421700	0.54201800	2.14427400
F	5.64107900	0.18181500	-0.04726500
C	3.43202500	4.84725300	-1.49706600
F	3.95510600	6.03647700	-2.02039500
F	3.19980900	4.03839000	-2.60059000
F	2.18809700	5.20161700	-0.97387200
C	1.07207200	-5.89873900	-0.99032600
F	0.97905500	-7.27688800	-1.21521700
F	0.18219100	-5.64078800	0.04681200
F	0.54250200	-5.31422400	-2.14476400
C	-5.89886300	-1.07188700	0.99018100
F	-7.27697000	-0.97891000	1.21532500
F	-5.31416200	-0.54218200	2.14446400
F	-5.64115300	-0.18206200	-0.04706800
C	-3.43205900	-4.84701000	-1.49756500
F	-3.95541500	-6.03612200	-2.02092600
F	-3.20017200	-4.03793700	-2.60094700
F	-2.18796100	-5.20155600	-0.97483400
C	-4.84705000	3.43182300	1.49757800
F	-6.03613700	3.95518200	2.02099400
F	-5.20161600	2.18771900	0.97489200
F	-4.03791300	3.19997500	2.60093300
C	-1.07212800	5.89884200	-0.99021400
F	-0.97918400	7.27702400	-1.21494200
F	-0.18225800	5.64082300	0.04691300
F	-0.54248000	5.31449400	-2.14470300

References

[1] S.-F. Liu, Q. Wu, H. L. Schmider, H. Aziz, N.-X. Hu, Z. Popović, S. Wang, *J. Am. Chem. Soc.* **2000**, *122*, 3671–3678.

[2] G. M. Sheldrick, *Acta Crystallogr*. *Sect. A: Found. Crystallogr.* **2015**, *71*, 3–8.

[3] G. M. Sheldrick, *Acta Crystallogr. Sect. A: Found. Crystallogr.* **2008**, *64*, 112–122.

[4] C. B. Hübschle, G. M. Sheldrick, B. Dittrich, *J. Appl. Crystallogr.* **2011**, *44*, 1281–1284.

[5] K. Brandenburg, C. Diamond, M. S. Visualization, *See also: http://www. Crystalimpact. Com/diamond* **2016**.

[6] C. F. Macrae, I. J. Bruno, J. A. Chisholm, P. R. Edgington, P. McCabe, E. Pidcock, L. Rodriguez-Monge, R. Taylor, J. Streek, P. A. Wood, *J. Appl. Crystallogr.* **2008**, *41*, 466–470.

[7] M. J. Frisch, G. W. Trucks, H. B. Schlegel, G. E. Scuseria, M. A. Robb, J. R. Cheeseman, G. Scalmani, V. Barone, G. A. Petersson, H. Nakatsuji, X. Li, M. Caricato, A. V. Marenich, J. Bloino, B. G. Janesko, R. Gomperts, B. Mennucci, H. P. Hratchian, J. V. Ortiz, A. F. Izmaylov, J. L. Sonnenberg, Williams, F. Ding, F. Lipparini, F. Egidi, J. Goings, B. Peng, A. Petrone, T. Henderson, D. Ranasinghe, V. G. Zakrzewski, J. Gao, N. Rega, G. Zheng, W. Liang, M. Hada, M. Ehara, K. Toyota, R. Fukuda, J. Hasegawa, M. Ishida, T. Nakajima, Y. Honda, O. Kitao, H. Nakai, T. Vreven, K. Throssell, J. A. Montgomery Jr., J. E. Peralta, F. Ogliaro, M. J. Bearpark, J. J. Heyd, E. N. Brothers, K. N. Kudin, V. N. Staroverov, T. A. Keith, R. Kobayashi, J. Normand, K. Raghavachari, A. P. Rendell, J. C. Burant, S. S. Iyengar, J. Tomasi, M. Cossi, J. M. Millam, M. Klene, C. Adamo, R. Cammi, J. W. Ochterski, R. L. Martin, K. Morokuma, O. Farkas, J. B. Foresman, D. J. Fox, Wallingford, CT, **2016**.

[8] M. D. Hanwell, D. E. Curtis, D. C. Lonie, T. Vandermeersch, E. Zurek, G. R. Hutchison, *J. Cheminformatics* **2012**, *4*, 17.

[9] T. Lu, F. Chen, *J. Comput. Chem.* **2012**, *33*, 580–592.

[10] C. Lee, W. Yang, R. G. Parr, *Phys*. *Rev*. *B* **1988**, *37*, 785–789.

[11] a) G. Petersson, M. A. Al-Laham, *J. Chem. Phys.* **1991**, *94*, 60813–6090; b) G. Petersson, A. Bennett, T. G. Tensfeldt, M. A. Al-Laham, W. A. Shirley, J. Mantzaris, *J. Chem. Phys.* **1988**, *89*, 2193–2218.

[12] S. Grimme, *Chem. Eur. J.* **2004**, *10*, 3423–3429.

[13] S. Durben, T. Baumgartner, *Inorg. Chem.* **2011**, *50*, 6823–6836.

[14] F. B. Dias, T. J. Penfold, A. P. Monkman, *Methods Appl. Fluoresc.* **2017**, *5*, 012001.
